# Optimal Weighted Tests for Replication Studies and the ‘Two‐Trials Rule’ With Multiple Hypotheses

**DOI:** 10.1002/sim.70638

**Published:** 2026-07-01

**Authors:** David S. Robertson, Thomas Jaki

**Affiliations:** ^1^ MRC Biostatistics Unit University of Cambridge Cambridge UK; ^2^ University of Regensburg Regensburg Germany

**Keywords:** familywise error rate, power, reproducibility, type I error rate control

## Abstract

Replication studies for scientific research are an important part of ensuring the reliability and integrity of experimental findings. In the context of clinical trials, the concept of replication has been formalized by the “two‐trials” rule, where two pivotal studies are required to show positive results before a drug can be approved. In experiments testing multiple hypotheses simultaneously, control of the overall familywise error rate (FWER) is additionally required in many contexts. The well‐known Bonferroni procedure controls the FWER, and a natural extension is to introduce weights into this procedure to reflect the a‐priori importance of hypotheses or to maximize some measure of the overall power of the experiment. In this article, we consider analyzing a replication study using an optimal weighted Bonferroni procedure, with the weights based on the results of the original study that is being replicated and the optimality criterion being to maximize the disjunctive power of the trial (the power to reject at least one non‐null hypothesis). We show that using the proposed procedure can lead to a substantial increase in the probability of success (PoS), is robust to changes in the effect sizes between the two studies, and recovers the submission wise type I error rate $\alpha^{2}$ of the two‐trials rule.

## 1 Introduction

Driven by long‐standing concerns of a reproducibility crisis in science [1], the importance of replicating scientific studies is well‐recognized [2]. Such replication studies are now commonplace in all areas of scientific research. In the context of clinical trials, for example, this has been formalized by the US Food and Drug Administration (FDA) in what is known as the *two‐trials rule*. This states that there should be “at least two pivotal studies, each convincing on its own” [3] before a drug is approved and “reflects the need for substantiation of experimental results, which has often been referred to as the need for replication of the finding” [4]. For trials with a single hypothesis, the two‐trials rule is usually satisfied by running two independent trials and requiring the results to be statistically significant at the standard (one‐sided) 2.5% level [5, 6]. Although very recently the FDA has signaled a shift away from the two‐trials rule as the default, two trials will still be required in some contexts [7].

In trials (and scientific experiments more generally) that test multiple hypotheses simultaneously, there is the added complication of how to handle this multiplicity. In confirmatory (or “pivotal”) trials, where the aim is to provide definitive results, the *familywise error rate* (FWER) is the typical error rate to be controlled [8, 9]. The FWER is defined as the (maximum) probability of falsely rejecting at least one null hypothesis, under any configuration of which hypotheses are null and non‐null. Control of the FWER at some small level α is seen as particularly important when the hypotheses are related in some way, such as when testing multiple doses of the same drug or multiple endpoints on the same group of patients.

Many multiple testing procedures have been proposed to control the FWER in clinical trials and other scientific contexts more generally [10]. A popular choice (and perhaps the most common by far) remains the well‐known Bonferroni procedure. When testing m hypotheses of interest $H_{1},\ldots ,H_{m}$ with corresponding p‐values denoted $p_{1},\ldots ,p_{m}$, the Bonferroni procedure rejects hypothesis $H_{i}$ if $p_{i}<\alpha /m$. A natural extension is to consider a *weighted* Bonferroni procedure, where instead of “splitting” the allowed type I error (α) equally between the hypotheses, predetermined weights $w_{i}$ are assigned to hypothesis $H_{i}$ (where these weights are non‐negative and sum up to 1). Hypothesis $H_{i}$ is then rejected if $p_{i}<w_{i}\alpha$. Note that setting $w_{i}\equiv 1/m$ recovers the standard (unweighted) Bonferroni procedure. Intuitively, weighting increases the power to reject hypothesis $H_{i}$ when $w_{i}>1/m$, and decreases the power when $w_{i}<1/m$, compared with using the standard Bonferroni procedure. These weights could be chosen based on the a‐priori relative importance of the hypotheses, or could be chosen to *optimize* different definitions of the power of the experiment. The latter is the approach we consider in this article.

The idea of choosing the weights to optimize power for Bonferroni procedures has received limited attention in the literature. Almost all of this has focused on maximizing either the mean power, the weighted mean power (where the weights reflect their a‐priori importance), or the expected number of rejections. In the context of fixed‐sequence tests for clinical trials, optimal weights have been provided numerically to maximize the weighted power [11] and the expected number of rejections [12]. In three related articles [13, 14, 15] in the context of replication studies for genomics, analytical formulae have been derived for weighted Bonferroni tests to obtain the optimal mean power.

As discussed in [11, 16], while the mean (weighted or unweighted) power is easier to optimize numerically, it has issues with interpretability and is not the most common definition of overall power used. For this reason, Xi and Chen [16] considered choosing the weights to maximize either the *disjunctive* or *conjunctive* power of a single trial with multiple hypotheses. The disjunctive power is the probability of rejecting at least one non‐null hypothesis, whereas the conjunctive power is the probability of rejecting all non‐null hypotheses [17]. The motivation for using the disjunctive power in the context of a clinical trial would be the desire to find at least one truly effective treatment, or demonstrate the effectiveness of a treatment on at least one of the (co‐)primary endpoints of the trial. Xi and Chen [16] showed how to maximize either the disjunctive or conjunctive power for both independent and correlated test statistics, with the optimal weights found numerically.

A key challenge in all of the above literature is that the true optimal weights depend on the true effect sizes (i.e., treatment effects in the clinical trial setting) associated with each hypothesis, which are unknown. In [11, 12, 16] this problem is handled by using a‐priori effect sizes for each hypothesis, corresponding to the effect sizes required for the desired marginal power and sample size calculations (for example). Meanwhile, in [13, 14, 15] a sample‐splitting approach was proposed, where part of the data from the experiment is used to estimate the true effect size, and the other part of the data is used for hypothesis testing. This latter approach can suffer from a substantial loss in power; however, due to the reduced sample size.

In this article, we take a different approach for the context of replication studies and the two trial rule. Given that an experiment (labelled “experiment 2”) is used as a replication study for another experiment (labelled “experiment 1”), we can use the data from experiment 1 to estimate the true effect sizes and hence calculate the optimal weights for testing the hypotheses in experiment 2. Note that we do not need to assume that these experiments are fully sequential (i.e., that experiment 2 starts after experiment 1 is completed). What is required is that the results of experiment 1 are available before the statistical analysis plan (SAP) for experiment 2 is finalized and the data are analyzed. This is particularly relevant for pharmaceutical drug development, where it is more common that the two trials are run essentially in parallel. We return to this issue in the Discussion.

Like in Xi and Chen [16], we consider maximizing the disjunctive power as our optimality criteria, but propose an alternative numerical solution to the optimization problem. We also consider the robustness of our proposal, in terms of what happens when the effect sizes in experiments 1 and 2 differ. The rest of the article proceeds as follows. Section 2 develops the theoretical framework for the optimal weighted Bonferroni procedure to maximize disjunctive power. In Section 3, we present a real‐life case study of a “two‐trials” submission to the FDA with multiple treatment groups and endpoints, followed by a case study from a preclinical cancer biology replication project. We present a comprehensive simulation study in Section 4 and conclude with a Discussion in Section 5.

## 2 Methods

Suppose we have m>1 hypotheses of interest denoted $H_{1},\ldots ,H_{m}$, where (without loss of generality) these hypotheses are of the form $H_{i}:\theta_{i}=0$ versus the alternatives $H_{i}^{\prime }:\theta_{i}>0$ for i=1,…,m. In the clinical trial context, $\theta_{i}$ could correspond to the treatment effect for experimental treatment i, or alternatively the treatment effect for a single experimental treatment as measured by endpoint i (in a trial with multiple endpoints). We let $\theta =(\theta_{1},\ldots ,\theta_{m})$ denote the vector of true parameter values and $𝒜=\{i:\theta_{i}>0\}$ denote the index set of alternative (i.e., non‐null) hypotheses.

Two independent trials/experiments are conducted to test this set of multiple hypotheses, resulting in two independent sets of standardized test statistics $T^{\left(j\right)}=(T_{1}^{\left(j\right)},\ldots ,T_{m}^{\left(j\right)})$ for j=1,2, where $T_{i}^{\left(j\right)}∼N(\theta_{i},1)$, at least asymptotically. The labelling j=1 corresponds to the experiment that is used to estimate the true effect sizes for experiment j=2. The resulting p‐values from the two experiments are denoted $p_{1}^{\left(j\right)},\ldots ,p_{m}^{\left(j\right)}$ for j=1,2, where $p_{i}^{\left(j\right)}=\bar{\Phi }(T_{i}^{\left(j\right)})$, $\bar{\Phi }(\cdot )=1-\Phi (\cdot )$ and Φ(·) denotes the standard normal cdf (cumulative distribution function).

### 2.1 Weighted Bonferroni Procedure

In order to control the FWER for each experiment, we use the (standard) Bonferroni procedure for experiment 1 and a *weighted* Bonferroni procedure for experiment 2, where these weights depend on the data from experiment 1. Hence, for experiment 1, hypothesis $H_{i}$ is rejected if $p_{i}^{\left(1\right)}<\alpha /m$. For experiment 2, hypothesis $H_{i}$ is rejected if $p_{i}^{\left(2\right)}<w_{i}\alpha$, where $w_{i}$ is the predetermined weight assigned to hypothesis $H_{i}$. Note that a weighted Bonferroni procedure could also be used for experiment 1 given some a‐priori weights, reflecting the relative importance of the hypotheses for example, but for simplicity we only consider using the usual unweighted procedure. More generally, *any* multiple hypothesis testing approach that controls the FWER could be used for experiment 1.

Given this formulation, we wish to use the observed test statistics from experiment 1 to choose *optimal* weights to use for experiment 2. The optimality criterion we consider in this article is to maximize the *disjunctive power*, that is, the probability to reject at least one non‐null hypothesis. Other definitions of power could be used, such as the average power or the conjunctive power, which we return to in the Discussion.

In what follows, we make the simplifying assumption that the test statistics in experiment 2 are all independent of one another. This can happen, for example, if each test statistic corresponds to data collected from distinct groups of subjects/patients. We return to the issue of correlated test statistics in the Discussion. The *marginal* probability to reject hypothesis $H_{i}$ using the weighted Bonferroni procedure in experiment 2 is

$$P(p_{i}^{\left(2\right)}<w_{i}\alpha )=P(T_{i}^{\left(2\right)}>{\bar{\Phi }}^{-1}(w_{i}\alpha ))=\bar{\Phi }\;\left({\bar{\Phi }}^{-1}(w_{i}\alpha )-\theta_{i}\right)$$

where ${\bar{\Phi }}^{-1}(x)=\Phi^{-1}(1-x)$. Assuming independence between the p‐values (or, equivalently, the test statistics), then the disjunctive power in experiment 2 is given by the following expression

$$1-\underset{i\in 𝒜}{\prod }P(p_{i}^{\left(2\right)}>w_{i}\alpha )=1-\underset{i\in 𝒜}{\prod }\Phi \;\left({\bar{\Phi }}^{-1}(w_{i}\alpha )-\theta_{i}\right).$$

We can then formulate the optimization problem (as in [16]) as follows:

(1) $$\begin{array}{ll} & \underset{w_{i}}{\max}\;\;1-\underset{i\in 𝒜}{\prod }\Phi \left({\bar{\Phi }}^{-1}(w_{i}\alpha )-\theta_{i}\right)\\ & \text{subject to}\;\overset{m}{\underset{i=1}{\sum }}w_{i}=1\\ & \;\;w_{i}\geq 0\;\text{for}\;i=1,\ldots ,m \end{array}$$

Given values for $\theta_{1},\ldots ,\theta_{m}$ (we return to this point later), this optimization problem can be solved using numerical non‐linear optimization algorithms to find the set of optimal Bonferroni weights, as detailed in [16]. However, particularly as the number of hypotheses m increases, common implementations of such algorithms, such as those provided by the nloptr R package can often run into computational issues (i.e., converging to a local rather than a global maximum). Xi and Chen [16] propose resolving these through the use of an exponentially increasing (in |𝒜|) number of starting points for the numerical optimization algorithm.

In this article, we propose an alternative solution in order to remove the need to use multiple starting points. It can be shown (see the Appendix, Section A for a proof) that given values for $\theta_{1},\ldots ,\theta_{m}$, the optimal Bonferroni weights can be written in the following form:

(2) $$\begin{array}{ll} w_{i} & =1(i\in 𝒜)\;\frac{1}{\alpha }\;\bar{\Phi }\left(\frac{\theta_{i}}{2}+\frac{1}{\theta_{i}}[\log(c)-\;\right.\\ & \;\left.\left.\sum_{j\in 𝒜,\;j\neq i}\log\left(\Phi ({\bar{\Phi }}^{-1}(w_{j}\alpha )-\theta_{j})\right)\right]\right) \end{array}$$

where 1(·) is the indicator function and the constant c>0 is chosen to ensure that $\sum_{i=1}^{m}w_{i}=1$. This system of nonlinear simultaneous equations can be solved numerically, for example by using the nelqslv package in R. In Appendix B we compare the performance of the different computational methods. These comparisons show that the use of the nelqslv approach is substantially quicker than using nloptr with multiple starting points, and also gives higher disjunctive power (i.e., the nloptr approach can still result in suboptimal weights). For example, for m=5 treatments, nelqslv is on average 28 times faster than nloptr and always has greater or equal disjunctive power, with a strictly greater disjunctive power in almost 10% of cases.

As an alternative to numerical solutions to the optimization problem (either via Equations 1 or 2), for small m (e.g., m=2,3) an exhaustive grid search can be performed. We evaluate the disjunctive power (given in Equation 1) over the space $(w_{1},\ldots ,w_{m})\in {\left[0,1\right]}^{m}$ subject to the constraint $\sum_{i=1}^{m}w_{i}=1$ to find the values of $\left(w_{1},\ldots ,w_{m}\right)$ that maximize the disjunctive power. As shown in Appendix B, the nelqslv approach is substantially faster for m>2 while never giving suboptimal weights in terms of disjunctive power.

### 2.2 Estimating Optimal Weights in Practice

In order to use equations (1) or (2), we need to provide values of $\theta_{1},\ldots ,\theta_{m}$ as well as the set of alternative hypotheses 𝒜. This is where the fact that experiment 2 is designed as a replication study for some earlier experiment 1 is key. Given that experiments 1 and 2 have the same underlying true parameters $\theta_{1},\ldots ,\theta_{m}$, we can use a consistent estimator ${\hat{\theta }}_{i}$ for $\theta_{i}$ based on the completed data from experiment 1 as our assumed value of $\theta_{i}$ used in the optimization problem and hence equations (1) or (2). In what follows, for simplicity we only consider the usual maximum likelihood estimator (MLE).

As for the set of alternative hypotheses 𝒜, given that trial 1 uses the usual (unweighted) Bonferroni procedure to perform the hypothesis tests, we can define $𝒜=\{i:p_{i}^{\left(1\right)}<\alpha /m\}$, that is, 𝒜 is the set of rejected hypotheses in experiment 1. In the remainder of this article, we focus on the two‐trials setting and use this to define 𝒜, although the same pattern of results can be seen with other choices of definition of 𝒜. For example, another option is to let $𝒜=\{i:{\hat{\theta }}_{i}>0\}$. One subtlety is what to do if 𝒜 is empty, that is, no hypotheses are rejected in experiment 1. A natural choice in this setting is to simply set all the weights equal to 1/m to recover the usual (unweighted) Bonferroni procedure, which we follow in this article.

## 3 Illustrative Examples

### 3.1 Bepreve Clinical Trials

The first example is the application to the US FDA Center for Drug Evaluation and Research seeking approval for the use of Bepotastine Besilate Ophthalmic Solution (Bepreve) as an eye drop treatment for ocular itching associated with allergic conjunctivitus [18]. In keeping with the FDA “two‐trials rule”, the applicant submitted two phase III Conjunctival Allergen Challenge (CAC) studies: ISTA‐BEPO‐CS01 (hereafter denoted as “Trial 1”) and CL‐S&E‐0409071‐P (hereafter denoted as “Trial 2”). Note that these trials were run sequentially, with trial 1 completed in July 2007 and trial 2 started in October 2007.

Both studies were identical in design (except that trial 1 was single center and trial 2 was multicenter), and evaluated the onset and duration of action of Bepreve 1.5% and Beptreve 1.0% in patients with acute allergic conjunctivitis. Trial subjects were randomized in a 1:1:1 ratio between the control, Beperve 1.0% and Bepreve 1.5%. There were 5 visits in a period of approximately 7 weeks, with visits 1 and 2 being for screening of eligible patients and the following 3 visits evaluating the efficacy and safety of Bepreve compared with control in alleviating the signs and symptoms of CAC‐induced allergic conjunctivitis.

The primary efficacy variables were subject‐evaluated ocular itching at 3 time points (3, 5, and 7 min post CAC) and investigator‐evaluated conjunctival redness also at 3 time points (7, 15, and 20 min post CAC). The criteria for overall statistical significance for Bepreve 1.0% or Bepreve 1.5% were for statistical significance for the primary efficacy variables to be achieved at a majority (2/3) of the time points, either visit 3 or 4 and additionally at visit 5. To adjust for multiplicity, the trials both used a standard (unweighted) Bonferroni procedure with overall (one‐sided) level α=0.05. A longitudinal analysis would also be possible, but this was not considered in the trials.

For simplicity, and in order to illustrate a wider set of results, we show how the weighted Bonferroni procedure could have been used to analyse the following subsets of possible analyses of the efficacy results:

- Visit 3, 7 min post‐CAC (4 comparisons: 2 endpoints, 2 drug concentrations)
- Visit 4, 7 min post‐CAC (4 comparisons: 2 endpoints, 2 drug concentrations)
- Visit 5, 7 min post‐CAC (4 comparisons: 2 endpoints, 2 drug concentrations)
- Conjunctival redness, Visit 3 (6 comparisons: 3 time points, 2 drug concentrations)
- Conjunctival redness, Visit 4 (6 comparisons: 3 time points, 2 drug concentrations)
- Conjunctival redness, Visit 5 (6 comparisons: 3 time points, 2 drug concentrations)

Table 1 shows the observed trial 1 means and resulting optimal Bonferroni weights, as well as the “original” and “new” adjusted p‐values (i.e., the adjusted p‐values associated with the unweighted and weighted Bonferroni procedures, respectively). Note that we used the p‐values reported for the t‐test. Given the relatively large degrees of freedom, the t‐distribution is a good approximation to the normal distribution in this case.

**TABLE 1:** Analyses of the efficacy results from the two pivotal trials of Bepreve for treatment of ocular itching associated with allergic conjunctivitis, where CAC = Conjunctival Allergen Challenge and CR = Conjunctival Redness. adj. = adjusted.

Trial 1		
Analysis	Means	Optimal weights
post‐CAC Visit 3	(3.93, 3.72, 2.22, 0.37)	(0.53, 0.47, 0, 0)
post‐CAC Visit 4	(4.99, 6.73, 2.50, 1.84)	(0.34, 0.60, 0.06, 0)
post‐CAC Visit 5	(6.48, 6.23, 4.19, 3.18)	(0.39, 0.36, 0.16, 0.08)
CR Visit 3	(2.22, 1.62, 1.24, 0.37, 0.21, −0.65)	(1/6, 1/6, 1/6, 1/6, 1/6, 1/6)
CR Visit 4	(2.50, 1.86, 1.62, 1.84, 1.55, 1.32)	(1, 0, 0, 0, 0, 0)
CR Visit 5	(4.19, 3.93, 3.25, 3.18, 2.57, 1.69)	(0.31, 0.27, 0.17, 0.16, 0.08, 0)

Trial 2		
Analysis	Original adj. *p*	New adj. *p*
post‐CAC Visit 3	(<0.001, 0.001, 0.021, 1)	(<0.001, 0.001, 1, 1)
post‐CAC Visit 4	(<0.001, <0.001, 0.002, 0.427)	(<0.001, <0.001, 0.011, 1)
post‐CAC Visit 5	(<0.001, <0.001, <0.001, 0.012)	(<0.001, <0.001, 0.001, 0.038)
CR Visit 3	(0.032, 0.101, 0.244, 1, 1, 1)	(0.032, 0.101, 0.244, 1, 1, 1)
CR Visit 4	(0.004, 0.214, 0.616, 0.640, 1, 1)	(0.001, 1, 1, 1, 1, 1)
CR Visit 5	(0.001, 0.012, 0.891, 0.019, 0.068, 1)	(0.000, 0.007, 0.874, 0.019, 0.135, 1)

The set of nonzero optimal weights corresponds to the set of hypotheses which are rejected in trial 1 (except for Conjunctival redness, Visit 3, where no hypotheses are rejected and hence equal weights are used). The hypotheses with the largest trial 1 means get the largest weights as expected, meaning that it is easier to reject the hypothesis in trial 2 (as seen by the smaller adjusted p‐values). In addition, hypotheses with trial 1 means close together will have weights that are almost equal, for example, $H_{1}$ and $H_{2}$ for Visit 3, 7 min post‐CAC. Another noticeable feature is that even if a hypothesis is rejected in trial 1, it may have a very low optimal weight if it has a substantially lower trial 1 mean than the other hypotheses, for example, $H_{3}$ for Visit 4, 7 min post‐CAC. In such cases, it becomes harder to subsequently reject this hypothesis in trial 2. However, overall—in terms of disjunctive power—one would still expect a gain given that the procedure is optimal for this criterion.

In all of the analyses presented in Table 1, there are no changes in the overall decision (i.e., which hypotheses are rejected in both trial 1 and trial 2). As an explicit (hypothetical) example of the overall decision changing, consider the Conjunctival Redness, Visit 3 analysis. Suppose the trial 1 mean for $H_{1}$ changed from 2.22 to 2.73 with corresponding p‐value going from 0.0148 to 0.004 and we use a different overall α=0.025, so that $H_{1}$ (and only $H_{1}$) is rejected in trial 1 when using the usual (unweighted) Bonferroni procedure. Then the optimal weights are (1,0,0,0,0) with trial 2 original adjusted p‐values of (0.032,0.101,0.244,1,1,1) and trial 2 new adjusted p‐values of (0.005,1,1,1,1,1). Hence, overall $H_{1}$ can be rejected when using the weighted Bonferroni procedure, but not the usual unweighted Bonferroni.

### 3.2 Cancer Biology Replication Study

The second example is taken from the Reproducibility Project:Cancer Biology (RP:CP) [19], which seeks to address concerns about reproducibility in scientific research by conducting replications of selected experiments from high‐profile articles in the field of cancer biology [20]. One such article is by Sirota et al. [21], which was replicated as described by Kandela et al. [22]. The experiments investigated the effect of cimetidine and doxorubicin on A549 lung adenocarcinoma cell lines, as well as the effect of cimetidine on ACHN renal cell carcinoma cell lines, all compared to vehicle control. The outcome of interest is tumor volume at day 11. To address multiplicity, the replication experiment used a standard (unweighted) Bonferroni procedure with overall (one‐sided) level α=0.05. We show how the weighted Bonferroni procedure could have been used in the analysis, assuming that the standard (unweighted) Bonferroni procedure was also used in the original experiment.

Table 2 shows the observed standardized difference in tumor volume compared with control at 11 days (as expressed by the observed t‐statistic) in the original experiment and the resulting optimal Bonferroni weights, as well as the “original” and “new” adjusted p‐values from the replication experiment. The first, second, and third entry in each vector corresponds to the cimetidine vs control comparison on the A549 cell lines, the cimetidine vs control comparison on the ACHN cell lines, and the doxorubicin vs control comparison on the A549 cell lines, respectively. As a sensitivity analysis, we also show what would have happened in the hypothetical scenario where the observed effect size of the cimetidine vs control comparison on the A549 cell lines had been slightly larger in the original experiment.

**TABLE 2:** Analysis of the results from the original and replication experiments.

Original means	Optimal weights	Rep. adj. p	Rep. new adj. p
(2.35, 1.05, 5.20)	(0, 0, 1)	(0.034, 1, 0.049)	(1, 1, 0.016)
(2.58, 1.05, 5.20)	(0.16, 0, 0.84)	(0.034, 1, 0.049)	(0.071, 1, 0.019)

*Note:* The first, second, and third entry in each vector corresponds to the cimetidine vs control comparison on the A549 cell lines, the cimetidine vs control comparison on the ACHN cell lines, and the doxorubicin vs control comparison on the A549 cell lines, respectively. The first row of the table corresponds to the observed data, while the second row corresponds to the hypothetical scenario where the effect size of the cimetidine vs control comparison on the A549 cell lines is slightly larger.

Abbreviations: adj. = adjusted; Rep. = Replication.

With the originally observed data, only the doxorubicin vs control comparison can be rejected in the original experiment. Hence, the optimal weights are simply 1 for this comparison and 0 for the others. In the hypothetical scenario where both the cimetidine vs control and doxorubicin vs control comparisons on the A549 cell lines can be rejected in the original experiment, we see that optimal weights become (0.16,0,0.84), and hence the adjusted p‐value for the cimetidine vs control experiments becomes larger than α and can no longer be rejected. This is an example of where using the weighted Bonferroni procedure leads to fewer rejections than the usual unweighted Bonferroni, which we explore further in the simulation studies in the next section.

## 4 Simulation Studies

In order to investigate the potential power gains of the proposed testing framework that uses a weighted Bonferroni test for experiment 2 compared with using an unweighted Bonferroni test, we conduct a simulation study. In what follows, for simplicity we use the setting and terminology of the “two‐trials rule” for confirmatory clinical trials. We now describe the simulation set‐up using the framework of Morris et al. [23].

### 4.1 Set‐Up

*Aim*: to compare the weighted and unweighted Bonferroni procedures in the context of the “two‐trials” rule.

*Data generating mechanisms*: We test m>1 treatment options in the first pivotal trial (trial 1), with parameter values $\theta =(\theta_{1},\ldots ,\theta_{m})$ corresponding to the true treatment means. The first pivotal trial yields standardized test statistics $T^{\left(1\right)}=(T_{1}^{\left(1\right)},\ldots ,T_{m}^{\left(1\right)})$ where $T_{i}^{\left(1\right)}∼N(\theta_{i},1)$. The second pivotal trial (trial 2) tests the same m treatment options, resulting in standardized test statistics $T^{\left(2\right)}=(T_{1}^{\left(2\right)},\ldots ,T_{m}^{\left(2\right)})$ where this time $T_{i}^{\left(2\right)}∼N(\theta_{i}^{\prime },1)$, that is, the second pivotal trial has true treatment means given by $\theta^{\prime }=(\theta_{1}^{\prime },\ldots ,\theta_{m}^{\prime })$. We first consider the setting where $\theta^{\prime }=\theta$ in Section 4.2 and investigate the robustness when $\theta^{\prime }\neq \theta$ in Section 4.4.

Table 3 shows the different simulation scenarios considered for trials testing m treatment options where m∈{2,3,5}. Note that for the scenario of two treatments, we start with a more comprehensive exploration of the parameter space in Section 4.2.1, that is, $\theta =(\theta_{1},\theta_{2})$ where $0<\theta_{1},\theta_{2}\leq 6$. In all simulation scenarios considered, we set α=0.05.

**TABLE 3:** Summary of simulation scenarios.

Scenario	Treatment means
Two treatments	θ=(0,θ)
	θ=(θ/2,θ)
	θ=(θ,θ)
Three treatments	θ=(0,0,θ)
	θ=(θ/2,θ,2θ)
Five treatments	θ=(0,0,0,0,θ)
	θ=(0,0,θ,θ,θ)
	$\theta =(\theta_{1},\theta_{2},\theta_{3},\theta_{4},\theta )$ where $\theta_{i}∼U[0,\theta ]$ independently

The scenarios where $\theta_{m}=\theta$ and $\theta_{i}=0$ for i≠m correspond to where only one treatment is effective and all others have null effect. The two scenarios θ=(θ/2,θ) and θ=(θ/2,θ,2θ) correspond to a “staircase” increasing pattern in the treatment means. The scenario $\theta =(\theta_{1},\theta_{2},\theta_{3},\theta_{4},\theta )$ where $\theta_{i}∼U[0,\theta ]$ independently corresponds to a setting where all treatments are effective but with a random true treatment effect between zero and θ.

*Estimands or other targets*: Rather than looking explicitly at the disjunctive (or indeed marginal) power in trial 2, we instead look at the *probability of success* (PoS) to reject a non‐null hypothesis in both trials, reflecting the decision‐making process used (this is also known as *submission‐level power*). In the more general experimental replication setting, this would correspond to replicating the same result of rejecting a null hypothesis in both experiments. Note that we do not show results for the FWER, since, as expected from theory, this is always controlled for each trial below the specified α level. We instead show results for the submission wise (also known as project wise) type I error rate.

*Methods*: We use the weighted and unweighted Bonferroni procedures as described in Section 2. In terms of the implementation of the weighted Bonferroni procedure, the numerical procedures used to find the optimal weights that maximize the disjunctive power are as follows:

- For m=2,3 treatments we use an exhaustive grid search over $(w_{1},\ldots ,w_{m})\in {\left[0,1\right]}^{m}$, subject to the constraint $\sum_{i=1}^{m}w_{i}=1$ with a grid size of 0.005. A grid search was used as it was computationally feasible and ruled out any numerical issues that can occur when using a nonlinear optimization or simultaneous equation solver.
- For m>3, an exhaustive grid search was no longer computationally feasible. Hence, we used the R package nelqslv to solve the set of simultaneous equations given in Equation (2) when there were more than 3 treatments from trial 1 with $p_{i}^{\left(1\right)}<\alpha /m$ (and used an exhaustive grid search otherwise).

*Performance measures*: The *marginal* PoS (mPoS) for a single non‐null hypothesis $H_{i}$ is the probability of rejecting $H_{i}$ in both trial 1 and trial 2. The *disjunctive* PoS (dPoS) is then the probability of rejecting at least one non‐null hypothesis in both trial 1 and trial 2. The submission‐wise type I error rate is the probability of making a false claim of success for any null hypothesis (while taking into account that a significant result on this endpoint has to be obtained in both trials). For the simulation scenarios, we use $10^{4}$ or $10^{5}$ simulation replicates (depending on the computational burden), which gives a Monte Carlo standard error of less than 0.50% and 0.16%, respectively, in absolute terms.

### 4.2 Consistent Treatment Effects

#### 4.2.1 Two Treatments

**FIGURE 1:**
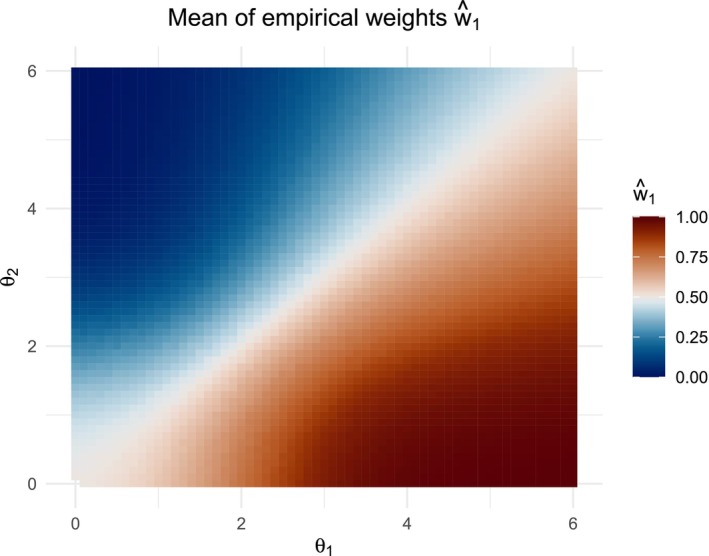
Heatmap showing the mean of the empirical optimal Bonferroni weights $\left[{ŵ}_{1}\right]$ across the range of parameter space of $\theta =(\theta_{1},\theta_{2})$ where $0\leq \theta_{1},\theta_{2}\leq 6$. Results are from $10^{4}$ simulations for each pair of values of $\left(\theta_{1},\theta_{2}\right)$.

We first explore the properties of the weighted Bonferroni approach across the parameter space of $\theta =(\theta_{1},\theta_{2})$ where $0\leq \theta_{1},\theta_{2}\leq 6$. Figure 1 shows the mean of the empirical weights given to hypothesis $H_{1}$, denoted ${ŵ}_{1}$, observed from $10^{4}$ simulation replicates for each pair of values of $\left(\theta_{1},\theta_{2}\right)$. Note that the empirical weight given to hypothesis $H_{2}$, ${ŵ}_{2}$, is given by ${ŵ}_{2}=1-{ŵ}_{1}$ by definition.

**FIGURE 2:**
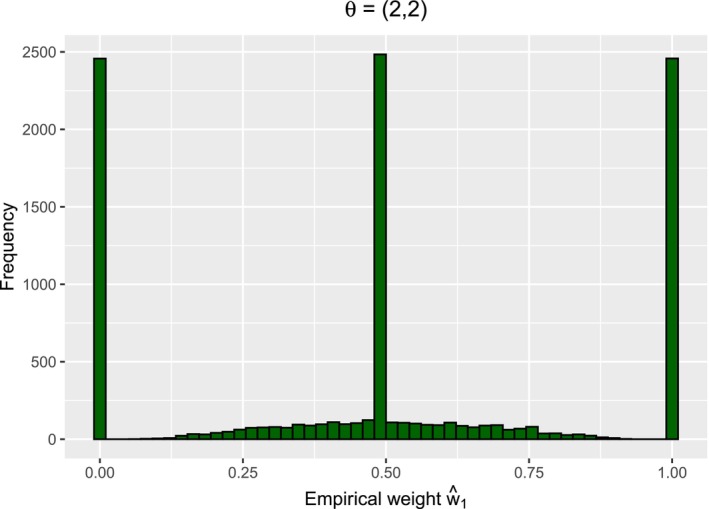
Histogram of the empirical optimal Bonferroni weights ${ŵ}_{1}$ when θ=(2,2). Results are from $10^{4}$ simulation replicates.

The mean values of ${ŵ}_{1}$ vary smoothly across the parameter space. As expected, when $\theta_{1}=\theta_{2}$ then ${ŵ}_{1}={ŵ}_{2}=0.5$, that is, the usual equal weights are recovered. We also see that ${ŵ}_{1}$ is an increasing function of the difference $\theta_{1}-\theta_{2}$. When $0<\theta_{2}<1$ then ${ŵ}_{1}\approx 1$ for $\theta_{1}\geq 3$, and conversely when $0<\theta_{1}<1$ then ${ŵ}_{1}\approx 0$ for $\theta_{2}\geq 3$. It is important to note, however, that taking the mean of ${ŵ}_{1}$ hides considerable stochasticity induced by the fact that the weights are based on values of $T_{i}^{\left(1\right)}∼N(\theta_{i},1)$, with the PoS additionally based on the values of $T_{i}^{\left(2\right)}∼N(\theta_{i},1)$. Figure 2 shows the histogram of the optimal Bonferroni weights observed from $10^{4}$ simulation replicates when θ=(2,2).

On average, the value of ${ŵ}_{1}$ (and hence ${ŵ}_{2}$) is indeed equal to 0.5. However, the realized values of ${ŵ}_{1}$ (and hence ${ŵ}_{2}$) have three large (and approximately equal) “spikes” at 0, 0.5, and 1. These spikes at 0 and 1 correspond to trial replicates where $T_{1}^{\left(1\right)}$ is substantially smaller and larger than $T_{2}^{\left(1\right)}$, respectively.

**FIGURE 3:**
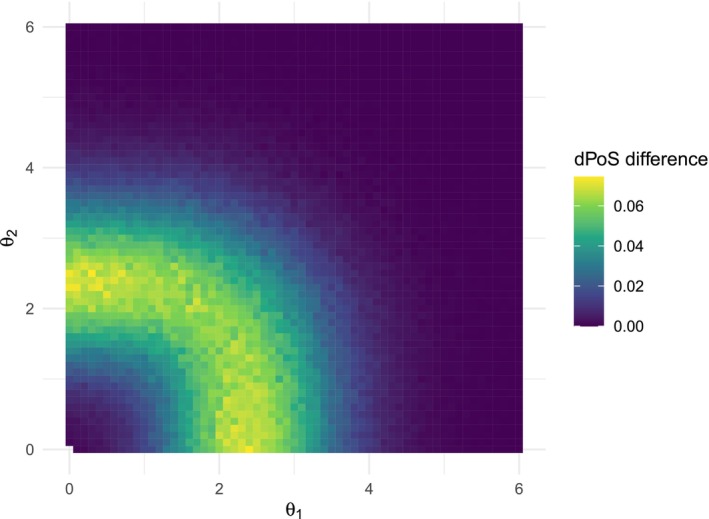
Heatmap showing the difference in the disjunctive Probability of Success (dPoS) across the range $\theta =(\theta_{1},\theta_{2})$ where $0\leq \theta_{1},\theta_{2},\leq 6$. Results are from $10^{4}$ simulations for each pair of values of $\left(\theta_{1},\theta_{2}\right)$. Positive values indicate where the dPoS of the weighted Bonferroni approach is higher than the dPoS of the unweighted Bonferroni approach.

We next evaluate how these weights translate into differences in the dPoS between the weighted and unweighted Bonferroni approaches across the same parameter space. Figure 3 shows a heatmap of the difference in dPoS (again based on $10^{4}$ simulation replicates), where a positive difference indicates where the dPoS of weighted Bonferroni is higher than the dPoS of unweighted Bonferroni.

For all regions of the parameter space, weighted Bonferroni has higher dPoS than unweighted Bonferroni, with observed increases of up to 0.075. As expected, the difference in dPoS is symmetric about the line $\theta_{1}=\theta_{2}$. The largest differences in dPoS occur in the region $\left\{0.5<\theta_{1},\theta_{2}<4\right\}$. When $\theta_{1}$ and $\theta_{2}$ are both small (<0.5), the dPoS is so low that weighting makes almost no difference. Conversely, when $\theta_{1}$ and $\theta_{2}$ are both large (>4) the dPoS is essentially equal to 1 when using the unweighted Bonferroni procedure, and so again weighting makes almost no difference.

Starting with a fixed small value of $\theta_{2}<1$ and increasing the value of $\theta_{1}$, the difference in dPoS increases steadily from 0 to a maximum of approximately 0.07 when $\theta_{2}\approx 2.5$, reflecting how the weight ${ŵ}_{1}$ smoothly increases from 0.5 toward 1. However, the difference in dPoS then starts to decrease despite the weight ${ŵ}_{1}$ continuing to increase toward 1. This makes intuitive sense as when $\theta_{1}$ is very large (and $\theta_{2}$ is small), the weighted and unweighted Bonferroni procedures will both have a mPoS very close to 1 for $H_{1}$. Hence, there is a “sweet spot” around $\theta_{2}\approx 2.5$ where the weighted Bonferroni approach makes the most difference in terms of dPoS.

Interestingly, the difference in dPoS is not zero when $\theta_{1}=\theta_{2}$ even though on average the empirical weights ${ŵ}_{1},{ŵ}_{2}$ are both equal to 0.5 (i.e., the unweighted case). For example, when $\theta_{1}=\theta_{2}=1.7$ there is a difference in dPoS of 0.05. The reason for this is that the empirical weights ${ŵ}_{1},{ŵ}_{2}$ take a range of values in [0,1] (as shown in Figure 2), and despite this distribution being symmetric about 0.5, the implications of weights being less than 0.5 and greater than 0.5 are not symmetric. Focusing on $H_{1}$ (a similar argument holds for $H_{2}$), consider the case when $T_{1}^{\left(1\right)}<T_{2}^{\left(1\right)}$ and so ${ŵ}_{1}<0.5$. The relative effect on the mPoS will be mitigated; however, since if $T_{1}^{\left(1\right)}$ is small enough, then $H_{1}$ will fail to be rejected in trial 1 and hence will fail to be rejected overall regardless of whether a weighted or unweighted Bonferroni procedure is used in trial 2. In contrast, now consider the case when $T_{1}^{\left(1\right)}>T_{2}^{\left(1\right)}$ and so ${ŵ}_{1}>0.5$. The relative effect on the mPoS is more substantial in this case, since if $T_{1}^{\left(1\right)}$ is large enough, then $H_{1}$ will be rejected in trial 1, ${ŵ}_{1}$ will be close to (or equal to) 1 and the power to reject $H_{1}$ in trial 2 (and hence the mPoS) will be noticeably higher when using the weighted compared with the unweighted Bonferroni procedure.

**FIGURE 4:**
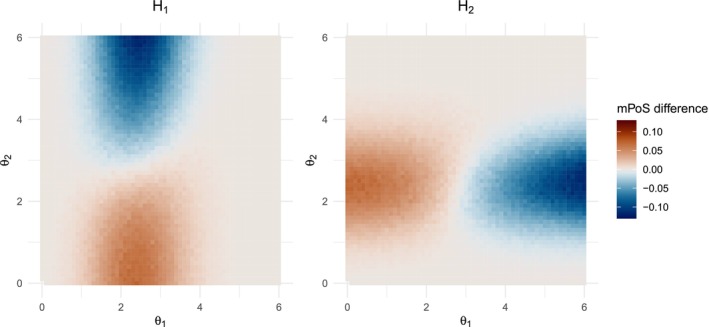
Heatmap showing difference in the marginal Probability of Success (mPoS) for $H_{1}$ and $H_{2}$ for $\theta =(\theta_{1},\theta_{2})$ where $0\leq \theta_{1},\theta_{2}\leq 6$. Results are from $10^{4}$ simulations for each pair of values of $\left(\theta_{1},\theta_{2}\right)$. Positive values indicate where the mPoS of the weighted Bonferroni approach is higher than the mPoS of the unweighted Bonferroni approach.

While there is a uniform improvement in the dPoS, the picture is not so clear cut when it comes to the mPoS for $H_{1}$ and $H_{2}$, as seen in the heatmap given in Figure 4.

In the region $\left\{0<\theta_{1},\theta_{2}<2.5\right\}$ the weighted Bonferroni procedure has a higher mPoS than the unweighted version for both $H_{1}$ and $H_{2}$, with essentially no difference in the mPoS in the regions $\left\{\theta_{1},\theta_{2}>4.5\right\}$, $\left\{\theta_{1}<0.5,\theta_{2}>2.5\right\}$ and $\left\{\theta_{1}>2.5,\theta_{2}<0.5\right\}$ for both $H_{1}$ and $H_{2}$. However, for the region $\left\{0.5<\theta_{1}<4.5,\theta_{2}>2.5\right\}$ the mPoS for $H_{1}$ is lower when using weighted Bonferroni compared with unweighted Bonferroni, and similarly for the region $\left\{0.5<\theta_{2}<4.5,\theta_{1}>2.5\right\}$ the mPoS for $H_{2}$ is lower. Looking at the heatmap of empirical mean weights ${ŵ}_{1}$ in Figure 1, the region where the mPoS for $H_{1}$ is lower corresponds to where $\theta_{1}$ is in the ‘interesting’ zone (i.e., not too small or too large) but $\theta_{2}$ is larger and so ${ŵ}_{1}<0.5$ (a similar argument holds for the region where the mPoS for $H_{2}$ is lower). This is the price that has to be paid to maximize the dPoS, although reassuringly the more substantial losses in mPoS only occur for very large values of $\theta_{1}$ or $\theta_{2}$ which coincide with very high values of dPoS anyway and (depending on the disease area) may be rather unlikely to be seen in practice.

We now take a closer look at the setting where θ=(θ/2,θ) with 0<θ<6. Figure C2 in the Appendix shows the dPoS as well as mPoS for $H_{1}$ and $H_{2}$ for the weighted and unweighted Bonferroni procedures (and their difference), averaged across $10^{5}$ simulation replicates. As already observed from Figure 1, the difference in the dPoS increases as θ increases, reaching a maximum of about 0.06 when θ≈2.3, which then starts to decrease. The weighted Bonferroni procedure has a noticeable gain in dPoS in regions that matter, that is, where the dPoS is relatively high but not quite reaching conventional levels of 80%.

The gains in dPoS are almost all driven by the gains in the mPoS for $H_{2}$, with the two plots closely matching. In contrast, the mPoS for $H_{1}$ is only very slightly higher for the weighted Bonferroni procedure for 0<θ<2.5, while for θ>2.5 the weighted Bonferroni procedure has an increasingly lower mPoS than the unweighted Bonferroni procedure, as already seen in Figure 4.

Table 4 shows the detailed results for rejection and non‐rejection for the two hypotheses for θ=3 based on 1000 simulations. The vast majority of the time, the weighted and unweighted Bonferroni procedures make the same overall rejections (973/1000 and 949/1000 for $H_{1}$ and $H_{2}$, respectively). There were 51/1000 cases of additional overall rejections of $H_{2}$ when using the weighted Bonferroni procedure. In contrast, there were 23/1000 additional overall rejections of $H_{1}$ when using the unweighted Bonferroni procedure.

**TABLE 4:** Overall rejections of $H_{1}$ and $H_{2}$ when using the weighted and unweighted Bonferroni procedures for θ=(3/2,3).

Test		Weighted	
		Rejection	No rejection
*H*1			
Unweighted	Rejection	101	4
	No rejection	23	872
*H*2			
Unweighted	Rejection	715	0
	No rejection	51	234

*Note:* Results are from 1000 simulation replicates.

Despite the guaranteed gains in dPoS, as we have seen, there can be losses in terms of mPoS. The scenario where θ=(θ/2,θ) gives large losses in the mPoS (for $H_{1}$) when θ≈5, but this is not the case in other scenarios, as we briefly describe below.

**FIGURE C1:**
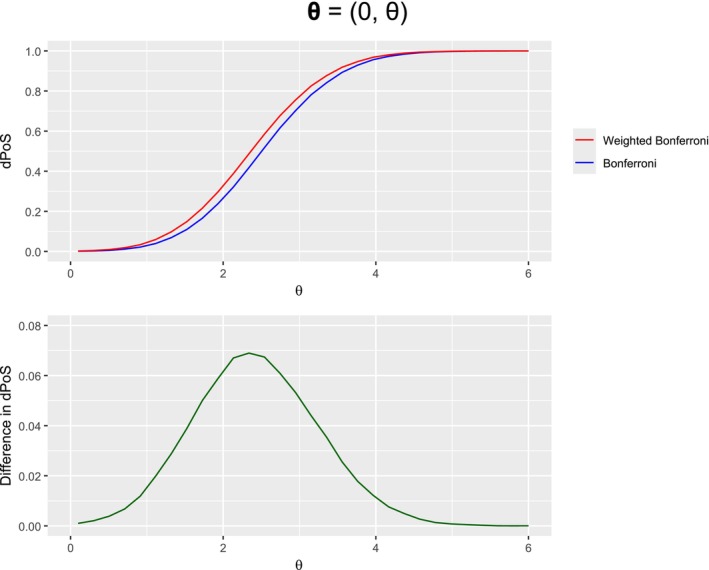
Disjunctive Probability of Success (dPoS) for $H_{2}$ for θ=(0,θ). Results are from $10^{5}$ simulation replicates.

**FIGURE C2:**
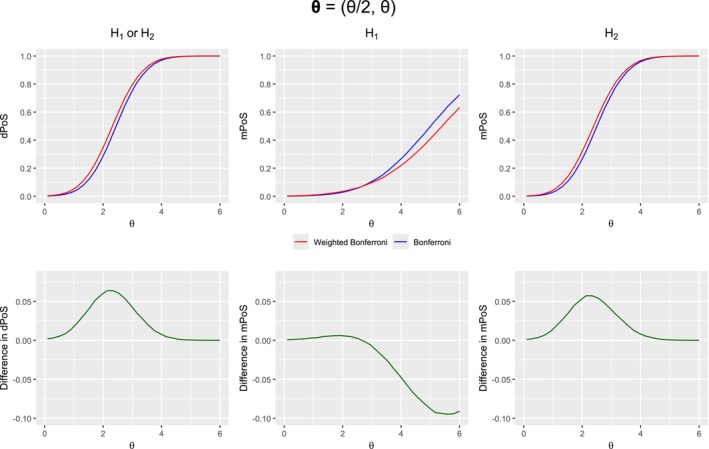
Disjunctive Probability of Success (dPoS) and marginal Probability of Success (mPoS) for $H_{1}$ and $H_{2}$, for θ=(θ/2,θ). Results are from $10^{5}$ simulation replicates.

One special case is the scenario where θ=(0,θ), where the mPoS is the same as the dPoS, since only $H_{2}$ is non‐null. Figure C1 in the Appendix shows the mPoS for $H_{2}$ using the unweighted and weighted Bonferroni procedures. The weighted Bonferroni procedure always has a higher mPoS than the unweighted procedure, with a maximum increase of almost 0.07 when θ≈2.3. Taking θ=3, we can also perform a comparison of the number of rejections of $H_{1}$ and $H_{2}$, with full results are shown in Table C1 in the Appendix, taken from 1000 simulation replicates. In 50/1000 cases, the weighted Bonferroni procedure rejected $H_{2}$ overall, whereas the unweighted Bonferroni procedure did not. There were no cases where the unweighted Bonferroni procedure rejected $H_{2}$ overall and the weighted Bonferroni procedure did not, illustrating the gain in mPoS.

**FIGURE C3:**
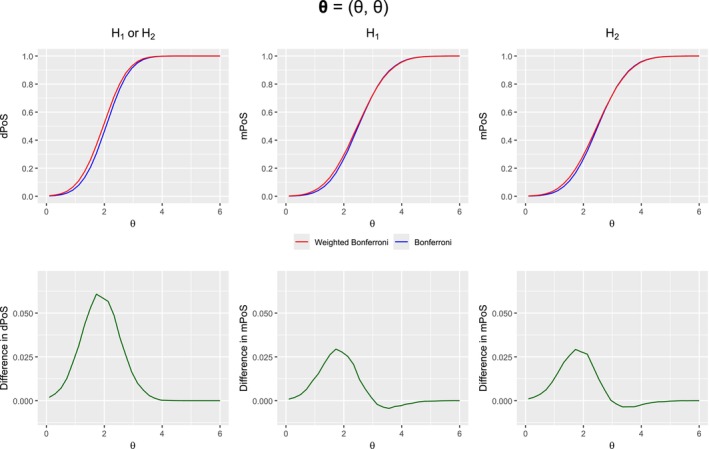
Disjunctive Probability of Success (dPoS) and marginal Probability of Success (mPoS) for $H_{1}$ and $H_{2}$, for θ=(θ,θ). Results are from $10^{5}$ simulation replicates.

Finally we consider the scenario with equal treatment means, that is, where θ=(θ,θ) with θ>0. Figure C3 in the Appendix shows the dPoS as well as the mPoS for $H_{1}$ and $H_{2}$ (which are identical up to simulation error). The gains in the mPoS are when θ<2.5, with the difference in mPoS again becoming negative for θ>2.5. However, this time the maximal loss is low, at less than 0.005. Taking θ=3, Table C2 in the Appendix shows the number of rejections of $H_{1}$ and $H_{2}$ from 1000 simulation replicates. There are substantially more cases where the weighted Bonferroni procedure rejects and the unweighted Bonferroni procedure does not reject compared with the other way round, illustrating the minimal loss in mPoS.

#### 4.2.2 m>2 Treatments

**FIGURE 5:**
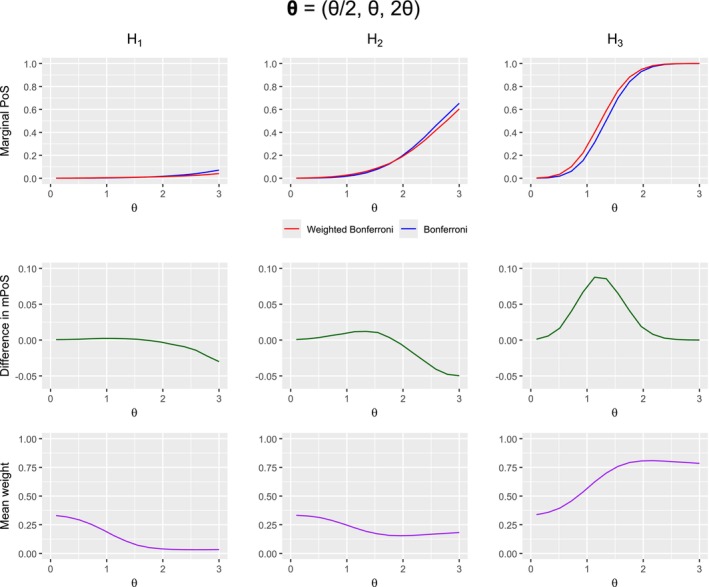
Marginal Probability of Success (mPoS) and mean empirical weights for $H_{1},H_{2},H_{3}$ for θ=(θ/2,θ,2θ). Results are from $10^{5}$ simulation replicates. Positive values of the difference in mPoS indicate where the mPoS of the weighted Bonferroni approach is higher than the unweighted Bonferroni approach.

**FIGURE C5:**
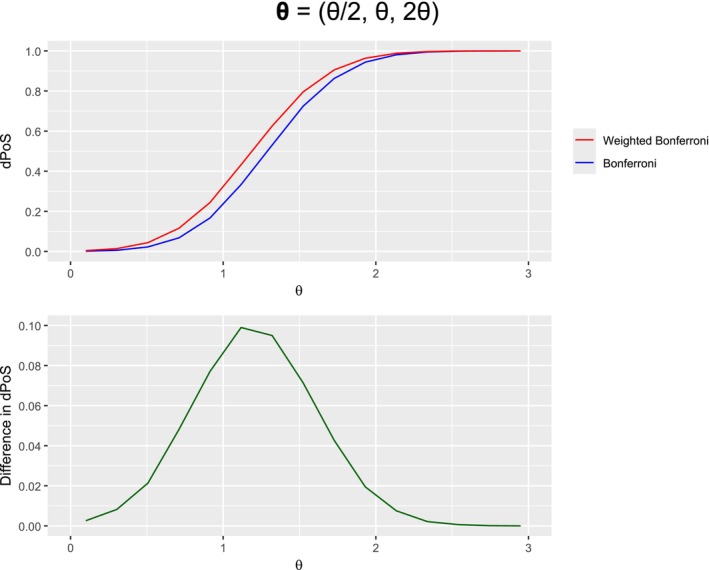
Disjunctive Probability of Success (dPoS) for θ=(θ/2,θ,2θ). Results are from $10^{5}$ simulation replicates. Positive values of the difference in dPoS indicate where the dPoS of the weighted Bonferroni approach is higher than the unweighted Bonferroni approach.

Moving to the setting with three treatments, we focus on the scenario θ=(θ/2,θ,2θ) with 0<θ<3 (we only go up to θ=3 since the dPoS is already equal to 1). Figure C5 in the Appendix shows the disjunctive PoS, while Figure 5 shows the mPoS for $H_{1},H_{2}$ and $H_{3}$ as well as the mean (empirical) weights ${ŵ}_{1},{ŵ}_{2},{ŵ}_{3}$.

In terms of the dPoS, the weighted Bonferroni procedure always has higher values than the unweighted Bonferroni procedure. There is a maximum gain of about 0.1 when θ≈1.1, with noticeable gains in the region where the dPoS is quite high but not quite at the conventional 0.8 or 0.9 level. This gain in dPoS is almost entirely driven by the large gains possible in mPoS for $H_{3}$. In contrast, for the mPoS for $H_{2}$ the weighted Bonferroni procedure performs worse than unweighted Bonferroni when θ>1.8, with a difference up to −0.05 for θ=3, reflecting how the weights ${ŵ}_{2}$ remain less than 1/3. Similarly, the difference in mPoS for $H_{1}$ is also negative when θ>1.8, with the weights becoming close to zero.

Like for the two treatment setting, this loss in mPoS (despite the guaranteed gain in the dPoS) is not seen in other scenarios. For example, consider the scenario where θ=(0,0,θ). The mPoS for $H_{3}$ (which is the same as the dPoS) is always greater when using the weighted Bonferroni procedure compared with the unweighted procedure, with a maximal gain in mPoS of 0.101.

**FIGURE 6:**
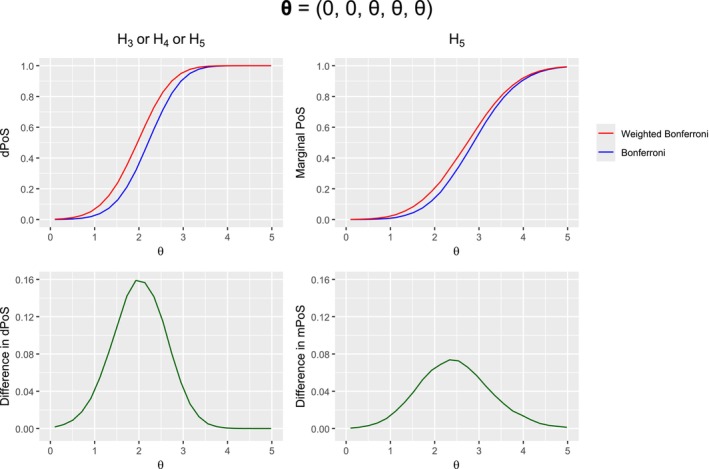
Disjunctive Probability of Success (dPoS) and marginal probability of success (mPoS) for $H_{5}$, for θ=(0,0,θ,θ,θ). Results are from $10^{5}$ simulation replicates.

Finally, we consider the setting with five treatments. We focus on the scenario, θ=(0,0,θ,θ,θ), where 0<θ<5. Figure 6 shows the dPoS as well as the mPoS for $H_{5}$, which will be the same plot as the mPoS for any of the non‐nulls $H_{3},H_{4},H_{5}$ (apart from simulation error). In terms of dPoS, large gains are seen when using the weighted Bonferroni procedure (up to 0.16). This includes interesting regions of the parameter space when using the weighted Bonferroni procedure. For example, when θ≈2.5 the mPoS for the weighted Bonferroni procedure is approximately 0.80, compared with around 0.70 when using unweighted Bonferroni. There are substantial gains in the marginal PoS for any of the non‐nulls (up to 0.074), with weighted Bonferroni always having a higher mPoS than unweighted Bonferroni.

**FIGURE C6:**
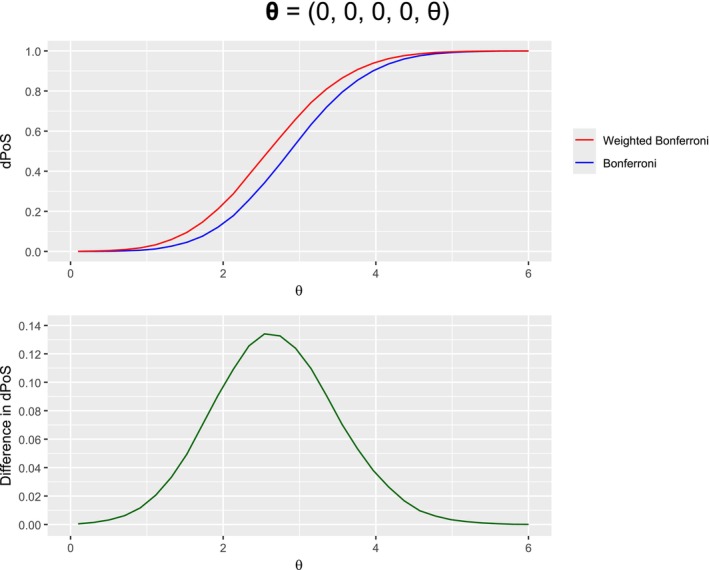
Probability of Success (PoS) for $H_{5}$ for θ=(0,0,0,0,θ). Results are from $10^{5}$ simulation replicates.

**FIGURE C7:**
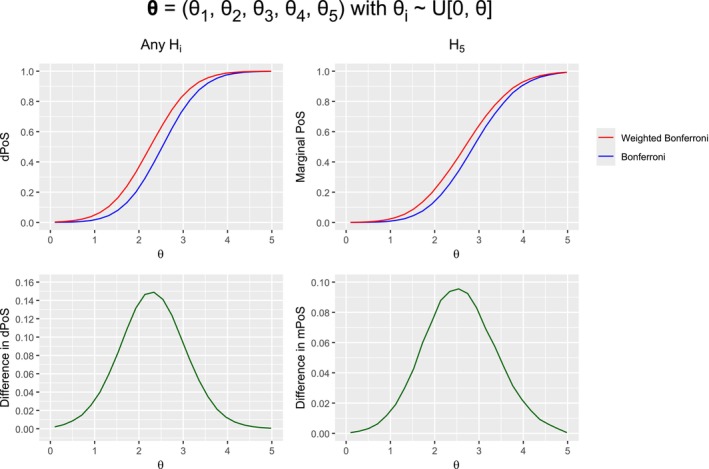
Disjunctive Probability of Success (dPoS) for $\theta =(\theta_{1},\theta_{2},\theta_{3},\theta_{4},\theta )$ and marginal Probability of Success (mPoS) for $H_{5}$, with $\theta_{i}∼U[0,\theta ]$ independently for i=1,2,3,4 and θ>0. Results are from $10^{5}$ simulation replicates.

Even larger gains in the mPoS (of up to 0.134) are seen in the scenario where θ=(0,0,0,0,θ), as seen in Figure C6 in the Appendix which shows the dPoS (which is the same as the mPoS for $H_{5}$ in this case). Finally, a more complex scenario is given by $\theta =(\theta_{1},\theta_{2},\theta_{3},\theta_{4},\theta )$, with $\theta_{i}∼U[0,\theta ]$ independently for i=1,2,3,4 and 0<θ<5. Figure C7 in the Appendix shows the dPoS as well as the mPoS for $H_{5}$. For the disjunctive PoS, the results are similar as for the previous scenarios with five treatments, with a maximum gain of 0.149. In terms of the mPoS, at least for the largest treatment effect (corresponding to $H_{5}$) we again see that there is a substantial gain in mPoS, up to 0.096.

### 4.3 Submission/Project Wise Error Rate

Thus far, we have focused on the PoS of the standard and weighted Bonferroni procedures. However, another important property in the clinical trial setting is the submission‐wise type I error rate (SWER), which is the probability of making a false claim of success for any endpoint (while taking into account that a significant result on this endpoint has to be obtained in both trials) [24]. This is also known as the project wise type I error rate in Rosenkranz [25], who argued that any procedure different from the two‐trials rule should have the same project wise type I error rate $\alpha^{2}$. To explore the SWER, we define some additional notation. Let ${ℋ}_{0}$ denote the set of null hypotheses with $m_{0}=|{ℋ}_{0}|$. For notational convenience, without loss of generality we re‐label the hypotheses so that (for $m_{0}>0$), $H_{1},\ldots ,H_{m_{0}}$ are the null hypotheses.

For the standard (unweighted) Bonferroni procedure, the SWER can be calculated analytically as follows

$$\begin{array}{ll} SWER & =P\left({⋃}_{i\in {ℋ}_{0}}\left\{p_{i}^{\left(1\right)}\leq \frac{\alpha }{m}\;,\;p_{i}^{\left(2\right)}\leq \frac{\alpha }{m}\right\}\right)\\ & =P\left({⋃}_{i=1}^{m_{0}}\left\{p_{i}^{\left(1\right)}\leq \frac{\alpha }{m}\;,\;p_{i}^{\left(2\right)}\leq \frac{\alpha }{m}\right\}\right)\\ & =1-{\left(1-\frac{\alpha^{2}}{m^{2}}\right)}^{m_{0}} \end{array}$$

where the equality on the last line follows using the inclusion‐exclusion principle and the fact that the events $\left\{p_{i}^{\left(1\right)}\leq \frac{\alpha }{m}\;,\;p_{i}^{\left(2\right)}\leq \frac{\alpha }{m}\right\}$ are independent with $P\left(\left\{p_{i}^{\left(1\right)}\leq \frac{\alpha }{m},\right.\right.\left.\left.p_{i}^{\left(2\right)}\leq \frac{\alpha }{m}\right\}\right)=\frac{\alpha^{2}}{m^{2}}$ for all $i\in {ℋ}_{0}$. In particular, we see that the SWER is maximized when $m_{0}=m$, that is, under the global null. Also note that we have the first order approximation $SWER\approx m_{0}\alpha^{2}/m^{2}$ and hence $SWER\approx \alpha^{2}/m$ under the global null.

For the weighted Bonferroni procedure, the SWER is given as follows

$$\begin{array}{ll} SWER & =P\left({⋃}_{i\in {ℋ}_{0}}\left\{p_{i}^{\left(1\right)}\leq \frac{\alpha }{m}\;,\;p_{i}^{\left(2\right)}\leq w_{i}\alpha \right\}\right)\\ & =P\left({⋃}_{i=1}^{m_{0}}\left\{p_{i}^{\left(1\right)}\leq \frac{\alpha }{m}\;,\;p_{i}^{\left(2\right)}\leq w_{i}\alpha \right\}\right)\\ & \approx \overset{m_{0}}{\underset{i=1}{\sum }}P\left(p_{i}^{\left(1\right)}\leq \frac{\alpha }{m}\;,\;p_{i}^{\left(2\right)}\leq w_{i}\alpha \right)\\ & =\overset{m_{0}}{\underset{i=1}{\sum }}P\left(p_{i}^{\left(2\right)}\leq w_{i}\alpha \;|\;p_{i}^{\left(1\right)}\leq \frac{\alpha }{m}\right)P\left(p_{i}^{\left(1\right)}\leq \frac{\alpha }{m}\right)\\ & =\frac{\alpha }{m}\overset{m_{0}}{\underset{i=1}{\sum }}P\left(p_{i}^{\left(2\right)}\leq w_{i}\alpha \;|\;p_{i}^{\left(1\right)}\leq \frac{\alpha }{m}\right) \end{array}$$

where the approximation follows using the inclusion‐exclusion principle and ignoring the higher‐order terms. Note that this implies that $m_{0}\alpha^{2}/m$ is an upper bound on the SWER for the weighted Bonferroni procedure. This follows since the approximation sign above can be replaced by ≤ using the Bonferroni inequality, and $P\left(p_{i}^{\left(2\right)}\leq w_{i}\alpha \;|\;p_{i}^{\left(1\right)}\leq \frac{\alpha }{m}\right)\leq P\left(p_{i}^{\left(2\right)}\leq w_{i}\alpha \right)\leq P\left(p_{i}^{\left(2\right)}\leq \alpha \right)=\alpha$ since $w_{i}\leq 1$ for all $i\in {ℋ}_{0}$.

Focusing on the setting of the global null, where the SWER of the usual Bonferroni procedure is maximized, heuristically we would expect that

$$P\left(p_{i}^{\left(2\right)}\leq w_{i}\alpha \;|\;p_{i}^{\left(1\right)}\leq \frac{\alpha }{m}\right)\approx P\left(p_{i}^{\left(2\right)}\leq \alpha \right)=\alpha$$

since if $p_{i}^{\left(1\right)}\leq \frac{\alpha }{m}$ then with high probability $w_{i}\approx 1$ (since $w_{i}\ll 1$ requires that $p_{j}^{\left(1\right)}<p_{i}^{\left(1\right)}\leq \frac{\alpha }{m}$ for at least one other $j\in {ℋ}_{0}$, which has low probability under the global null). Hence we have the following approximation under the global null:

$$SWER\approx \frac{\alpha }{m}\overset{m}{\underset{i=1}{\sum }}\alpha =\alpha^{2}$$

We verify this heuristic approximation empirically in Table 5 by simulation under the global null, using $10^{6}$ replicates (note that the Monte Carlo error is less than 0.00022). The SWER for the weighted Bonferroni is shown to be very close to $\alpha^{2}$. In contrast, the SWER for Bonferroni is very close to $\alpha^{2}/m$.

**TABLE 5:** Submission‐wise error rate (SWER) under the global null for m=2,3,5 hypotheses.

m	SWER under global null		
	Bonferroni (analytical)	Weighted Bonferroni (heuristic)	Weighted Bonferroni (empirical)
2	0.00125	0.0025	0.0024
3	0.00083	0.0025	0.0024
5	0.00050	0.0025	0.0024

*Note:* Results are from $10^{6}$ simulation replicates.

In summary, using the standard Bonferroni procedure leads to a maximum SWER of $\alpha^{2}/m$, which is much lower than the recommended $\alpha^{2}$ of Rosenkranz [25]. However, using the weighted Bonferroni procedure recovers a SWER of $\alpha^{2}$ under the global null, the same as the two‐trials rule.

### 4.4 Robustness

The assumption that experiments 1 and 2 have the same underlying true parameters $\theta_{1},\ldots ,\theta_{m}$ is a strong one, so in the following simulation studies we also consider what happens when this assumption is violated. That is, the setting where $T_{i}^{\left(1\right)}∼N(\theta_{i},1)$ but $T_{i}^{\left(2\right)}∼N(\theta_{i}^{\prime },1)$ where $\theta_{i}^{\prime }\neq \theta_{i}$. Such discrepancies could occur for a number of reasons, including due to systematic differences in prognostic variables between the populations assessed in experiments 1 and 2, and/or some form of temporal trend that causes the results in the later experiment 2 to be systematically different from the earlier experiment 1. In the context of replication studies, another potential feature is *publication bias*, so that the results from experiment 1 are in fact biased high (i.e., to give a ‘positive’ result that is published, when in fact the true effect is null).

**FIGURE 7:**
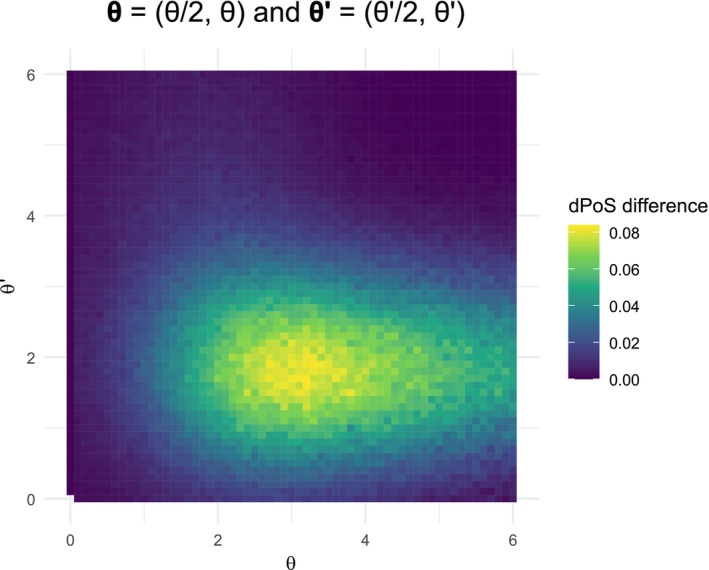
Heatmap showing difference in the disjunctive Probability of Success (dPoS) when trial 1 has treatment means given by θ=(θ/2,θ) while trial 2 has treatment means given by $\theta^{\prime }=(\theta^{\prime }/2,\theta^{\prime })$, where $0\leq \theta ,\theta^{\prime }\leq 3$. Results are from $10^{4}$ simulations for each pair of values of $\left(\theta ,\theta^{\prime }\right)$. Positive values indicate where the dPoS of the weighted Bonferroni approach is higher than the dPoS of the unweighted Bonferroni approach.

To assess robustness, we focus on the two treatment setting for simplicity and assume trial 1 has treatment means θ=(θ/2,θ) while trial 2 has treatment means $\theta^{\prime }=(\theta^{\prime }/2,\theta^{\prime })$, where $0\leq \theta ,\theta^{\prime }\leq 6$. Figure 7 shows a heatmap of the difference in the dPoS between the weighted and unweighted Bonferroni procedures. Despite the sometimes very large differences between θ and $\theta^{\prime }$, the weighted Bonferroni procedure still always has equal or higher dPoS compared with the unweighted Bonferroni procedure. Interestingly, this misspecified model with $\theta^{\prime }\neq \theta$ can lead to *larger* gains in dPoS compared with when $\theta^{\prime }=\theta$ for some regions of the parameter space.

**FIGURE 8:**
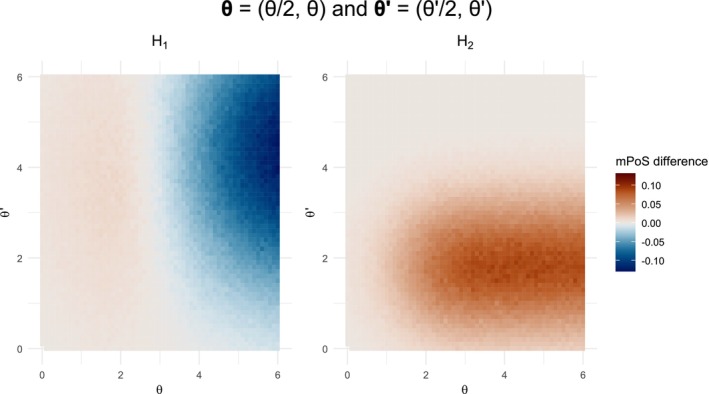
Heatmap showing difference in the marginal Probability of Success (mPoS) for $H_{1}$ and $H_{2}$ when trial 1 has treatment means given by θ=(θ/2,θ) while trial 2 has treatment means given by $\theta^{\prime }=(\theta^{\prime }/2,\theta^{\prime })$, where $0\leq \theta ,\theta^{\prime }\leq 6$. Results are from $10^{4}$ simulations for each pair of values of $\left(\theta ,\theta^{\prime }\right)$. Positive values indicate where the mPoS of the weighted Bonferroni approach is higher than the mPoS of the unweighted Bonferroni approach.

In terms of the difference in the mPoS, as seen in Figure 8, for $H_{2}$ the misspecified means still do not lead to a loss in mPoS compared with the unweighted Bonferroni procedure. For the region $\left\{\theta >1,\;0.5<\theta^{\prime }<3\right\}$ we in fact see an increase in the mPoS difference compared to when $\theta =\theta^{\prime }$. As for the mPoS for $H_{1}$, when θ<2.5 there is minimal difference between the mPoS. However for θ>2.5 then regardless of the value of $\theta^{\prime }$, there is a loss in mPoS, although this is worst in the region $\left\{\theta >4,\theta^{\prime }>2\right\}$.

**FIGURE 9:**
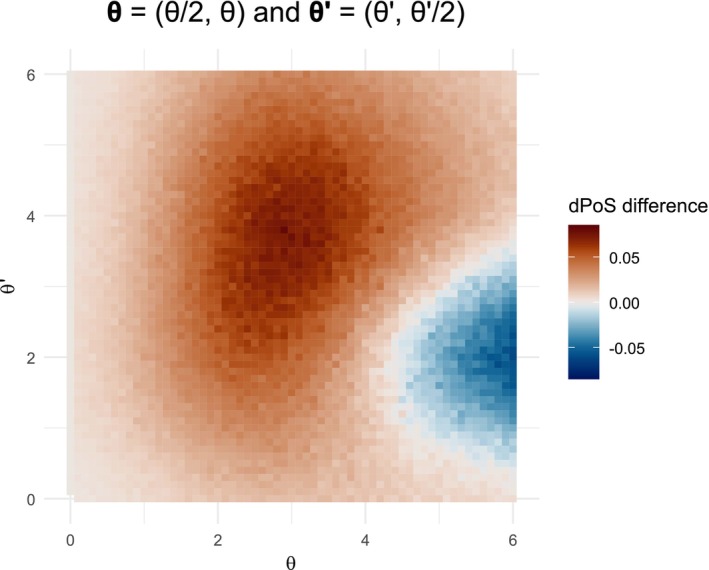
Heatmap showing difference in the disjunctive Probability of Success (dPoS) when trial 1 has treatment means given by θ=(θ/2,θ) but trial 2 has treatment means given by $\theta^{\prime }=(\theta^{\prime },\theta^{\prime }/2)$, where $0\leq \theta ,\theta^{\prime }\leq 6$. Results are from $10^{4}$ simulations for each pair of values of $\left(\theta ,\theta^{\prime }\right)$. Positive values indicate where the dPoS of the weighted Bonferroni approach is higher than the dPoS of the unweighted Bonferroni approach.

To find a scenario where the weighted Bonferroni procedure has a lower dPoS than the unweighted Bonferroni procedure, we need to be more extreme in the difference in the means between the two trials. To this end, we again assume trial 1 has treatment means θ=(θ/2,θ), but this time assume trial 2 has treatment means $\theta^{\prime }=(\theta^{\prime },\theta^{\prime }/2)$ where $0\leq \theta ,\theta^{\prime }\leq 6$. Figure 9 shows the resulting heatmap of the difference in dPoS. This time, in the region centered around $\theta^{\prime }=2$ and θ>4 there is a decrease in dPoS of up to 0.063, driven by a relatively large drop in the mPoS of $H_{1}$ due to the badly misspecified weights. Arguably though the magnitude of the discrepancy between the two trials where this happens is large enough to be unrealistic in many trial scenarios.

**FIGURE C8:**
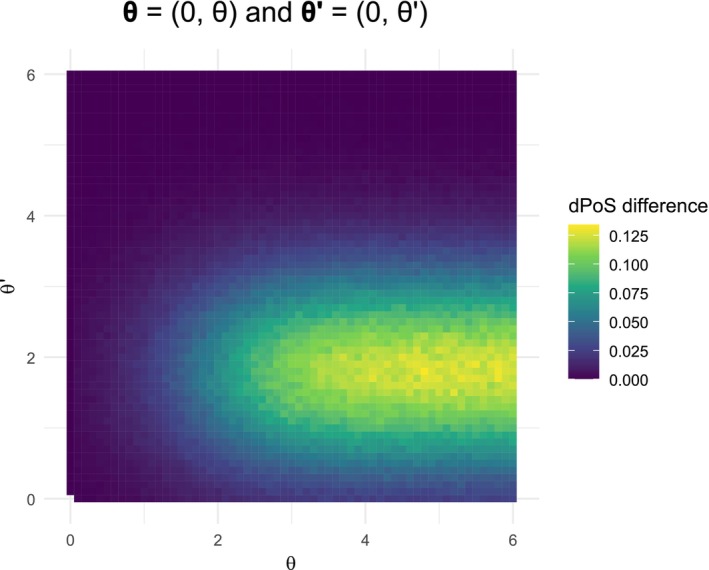
Heatmap showing difference in the disjunctive Probability of Success (dPoS) (which is the same as the marginal Probability of Success for $H_{2}$) when trial 1 has treatment means given by θ=(0,θ) while trial 2 has treatment means given by $\theta^{\prime }=(0,\theta^{\prime })$, where $0\leq \theta ,\theta^{\prime }\leq 3$. Results are from $10^{4}$ simulations for each pair of values of $\left(\theta ,\theta^{\prime }\right)$. Positive values indicate where the dPoS of the weighted Bonferroni approach is higher than the dPoS of the unweighted Bonferroni approach.

**FIGURE C9:**
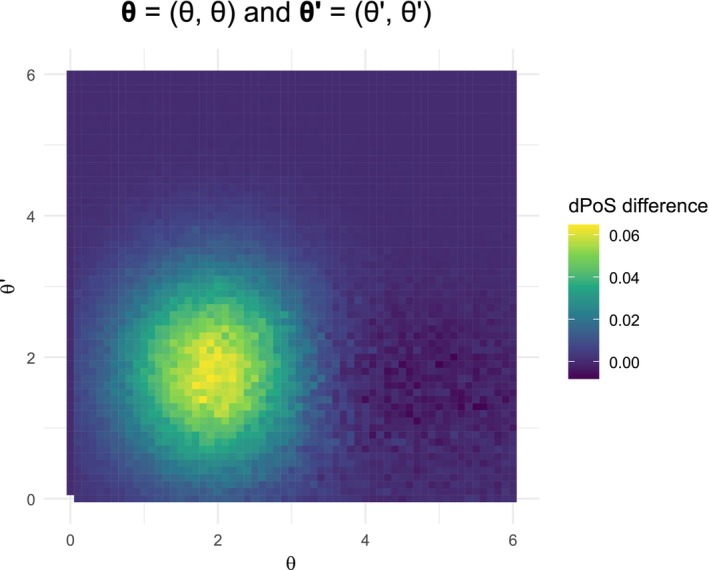
Heatmap showing difference in the disjunctive Probability of Success (dPoS) when trial 1 has treatment means given by θ=(θ,θ) while trial 2 has treatment means given by $\theta^{\prime }=(\theta^{\prime },\theta^{\prime })$, where $0\leq \theta ,\theta^{\prime }\leq 3$. Results are from $10^{4}$ simulations for each pair of values of $\left(\theta ,\theta^{\prime }\right)$. Positive values indicate where the dPoS of the weighted Bonferroni approach is higher than the dPoS of the unweighted Bonferroni approach.

Further results for robustness in the two‐treatment setting can be found in the Appendix. Figure C8 shows a heatmap of the difference in the dPoS (which is the same as the mPoS for $H_{2}$) between the weighted and unweighted Bonferroni procedures when trial 1 has treatment means θ=(0,θ) while trial 2 has treatment means $\theta^{\prime }=(0,\theta^{\prime })$. Again, we see that the dPoS of the weighted Bonferroni procedure is always equal to or higher than that of the unweighted Bonferroni procedure. Finally, we consider the setting where trial 1 has treatment means θ=(θ,θ) while trial 2 has treatment means $\theta^{\prime }=(\theta^{\prime },\theta^{\prime })$. The resulting heatmap of the difference in dPoS is given in Figure C9. Throughout the whole parameter space the dPoS of the weighted Bonferroni procedure is higher than that of the unweighted Bonferroni, except for the region $\left\{\theta >3,\theta^{\prime }<3\right\}$ where the difference can go very slightly negative (at most −0.005).

## 5 Discussion

The use of the proposed weighted Bonferroni procedure can lead to a substantial gain in the dPoS (compared with using the usual unweighted Bonferroni procedure) in the context of the “two‐trials” rule for confirmatory clinical trials, and also more generally in replication studies. Importantly, such gains can be realized in “interesting” regions of the parameter space where the dPoS is relative high when using the unweighted Bonferroni procedure, but not quite reaching conventional levels of 80% or 90%. The magnitude of these gains also increases with the number of tested hypotheses m (i.e., treatment options), since there is more scope to deviate from the usual equal weighting of (1/m,…,1/m). For example, the maximum gain in the dPoS increases from 0.069 to 0.101 to 0.134 for θ=(0,θ), θ=(0,0,θ) and θ=(0,0,0,0,θ), respectively. At the same time, the weighted Bonferroni procedure recovers the same SWER of $\alpha^{2}$ as the two‐trial rule.

When the true treatment means in trials 1 and 2 are the same, weighted Bonferroni has uniformly higher dPoS than unweighted Bonferroni, as would be expected from theory. However, even when the true treatment means in trials 1 and 2 differ, the weighted Bonferroni procedure is surprisingly robust in terms of still having higher dPoS, at least in the two‐treatment setting. We would expect this robustness to also be seen with a higher number of treatments, although to a lesser extent given the greater scope for the relative orderings of treatment effect to (dramatically) change between trials 1 and 2.

As seen in the simulation results, there is often a trade‐off between maximizing the dPoS (and hence the mPoS for treatments with the highest trial 1 means) and loss in mPoS for treatments with more “moderate” treatment effects in trial 1. This is somewhat inevitable given that the optimization criterion used is the disjunctive power of trial 2, meaning that the treatments with the highest trial 1 means are given the highest weights and hence treatments with lower trial 1 means must be given lower weights. If the interest is in the mPoS of more/all treatments in the trial, then other optimization criteria may be more appropriate to consider and used for the weighting of the procedure. As briefly mentioned in the Introduction, such optimization criteria include the mean power or conjunctive power of trial 2. However, these alternative optimality criteria will also have their own potential trade‐offs, and the choice depends on the trial context and aims.

As mentioned in the Introduction, the proposed procedure requires that the results of experiment 1 are available before the SAP for experiment 2 is finalized and the data are analysed. Particularly in the regulated setting of pharmaceutical drug development, in practice a master protocol would ideally be written prior to both studies starting, with the choice of method specified. Given that often it is the same sponsor who plans and runs both studies within a two‐trials framework, this is likely to be doable in practice, even for trials that are planned in parallel. From a regulatory point of view, there may still be concern about inducing dependency between the two studies. However, arguably this dependency is rather “weak” in that only prespecified weights for the hypotheses are used for the analysis of trial 2, and the FWER (and SWER) is still controlled.

As pointed out by an anonymous reviewer, another practical consideration is that the observed effect (with or without uncertainty) from experiment 1 could also be used for the sample size calculation for experiment 2. This would also affect the (conditional or predictive) power of the submission and would be an interesting area of future research. In addition, as shown throughout the article, often the optimal weight for a hypothesis will be zero. This reflects how the hypothesis can never be rejected overall (since it cannot be rejected from experiment 1). However, effect estimates etc. can still be presented for the effect corresponding to that hypothesis. The inability to reject the hypothesis in the experiment 2 bears some similarities to hierarchical testing, where (for an example with a single primary and secondary hypothesis) the hypothesis corresponding to a secondary endpoint may not be able to be rejected if the hypothesis corresponding to the primary endpoint is not rejected first. This also is closely related to the proposed methodology in Held (2024) [6], where for a single hypothesis per trial, a sequential analysis of trial results is proposed and a stop for futility after trial 1 is also possible.

As a simplifying assumption, in this article, we have considered the setting where the test statistics (or equivalently, the p‐values) in trial 2 are independent. In practice, depending on the trial context, these test statistics may be correlated (for example, if each hypothesis corresponds to a different endpoint measured on the same group of patients). It is important to note that even if the test statistics have some arbitrary correlation structure (even negative correlations), the proposed weighted Bonferroni procedure will still guarantee FWER control for trial 2 by definition. It is in the disjunctive power of trial 2 that ignoring the correlation structure will make an impact. Conceivably, using the weighted Bonferroni procedure assuming independence could result in a lower disjunctive power in trial 2 (and hence lower dPoS) than using the unweighted Bonferroni procedure. However, the results in [16] suggest that it is only with very high correlation values (i.e., >0.7) that the optimal weights change (at least in the setting with equal correlations and equal treatment means).

In the Supporting Information, we present simulation results showing the performance of the weighted and unweighted Bonferroni under different correlation structures. We assume that $\left(T_{1}^{\left(j\right)},\cdots \;,T_{m}^{\left(j\right)}\right)$ follow a multivariate normal distribution for j=1,2, with mean vector $\left(\theta_{1},\ldots ,\theta_{m}\right)$ and covariance matrix ∑ where

$$\sum_{i,k}=\left\{\begin{array}{l} 1\;\text{if}\;i=k\\ \rho \;\text{if}\;i\neq k \end{array}\right.$$

We vary ρ∈{0.25,0.5,0.75}. Reassuringly, even for ρ=0.75, there is little difference in the empirical disjunctive and marginal powers compared with the independent setting. In particular, the weighted Bonferroni procedure assuming independence still always has higher disjunctive power than the unweighted Bonferroni. If (highly) correlated test statistics are a concern, then the methods proposed in [16] for finding optimal weights for the disjunctive power accounting for correlation could be used.

Finally, we have focused on weighted Bonferroni procedures in this article. There are a number of reasons for this, including simplicity, ease of interpretability in terms of adjusted p‐values and confidence intervals, and robustness to deviations from independence in terms of still guaranteeing FWER control. However, an alternative would be to consider weighted Holm procedures (also known as Bonferroni‐Holm), which is a stepdown procedure that also guarantees FWER control for arbitrary correlation between the test statistics. This would require a reformulation of the optimization problem to take into account the more complex procedure used in trial 2. Similarly, if it is known that the test statistics are positively correlated then one could consider using a weighted Hochberg procedure. Finally, if the full correlation structure was known in terms of a multivariate normal distribution (which would be the case if we are comparing multiple independent normally‐distributed treatment arms against a common control, for example), then a weighted Dunnett test could be considered instead.

## Funding

The authors have nothing to report.

## Conflicts of Interest

The authors declare no conflicts of interest.

## Supporting information




**Data S1: Figure S1:** PoS for $H_{2}$ for θ=(0,θ) and ρ=0. Results are from $10^{5}$ simulation replicates. **Figure S2:** PoS for $H_{2}$ for θ=(0,θ) and ρ=0.25. Results are from $10^{5}$ simulation replicates. **Figure S3:** PoS for $H_{2}$ for θ=(0,θ) and ρ=0.5. Results are from $10^{5}$ simulation replicates. **Figure S4:** PoS for $H_{2}$ for θ=(0,θ) and ρ=0.75. Results are from $10^{5}$ simulation replicates. **Figure S5:** Disjunctive Probability of Success (dPoS) for and marginal Probability of Success (mPoS) for $H_{1}$ and $H_{2}$, for θ=(θ/2,θ) and ρ=0. Results are from $10^{5}$ simulation replicates. **Figure S6:** Disjunctive Probability of Success (dPoS) for and marginal Probability of Success (mPoS) for $H_{1}$ and $H_{2}$, for θ=(θ/2,θ) and ρ=0.25. Results are from $10^{5}$ simulation replicates. **Figure S7:** Disjunctive Probability of Success (dPoS) for and marginal Probability of Success (mPoS) for $H_{1}$ and $H_{2}$, for θ=(θ/2,θ) and ρ=0.5. Results are from $10^{5}$ simulation replicates. **Figure S8:** Disjunctive Probability of Success (dPoS) for and marginal Probability of Success (mPoS) for $H_{1}$ and $H_{2}$, for θ=(θ/2,θ) and ρ=0.75. Results are from $10^{5}$ simulation replicates. **Figure S9:** Disjunctive Probability of Success (dPoS) and marginal Probability of Success (mPoS) for $H_{1}$ and $H_{2}$, for θ=(θ,θ) and ρ=0. Results are from $10^{5}$ simulation replicates. **Figure S10:** Disjunctive Probability of Success (dPoS) and marginal Probability of Success (mPoS) for $H_{1}$ and $H_{2}$, for θ=(θ,θ) and ρ=0.25. Results are from $10^{5}$ simulation replicates. **Figure S11:** Disjunctive Probability of Success (dPoS) and marginal Probability of Success (mPoS) for $H_{1}$ and $H_{2}$, for θ=(θ,θ) and ρ=0.5. Results are from $10^{5}$ simulation replicates. **Figure S12:** Disjunctive Probability of Success (dPoS) and marginal Probability of Success (mPoS) for $H_{1}$ and $H_{2}$, for θ=(θ,θ) and ρ=0.75. Results are from $10^{5}$ simulation replicates. **Figure S13:** Disjunctive Probability of Success (dPoS) for θ=(0,0,θ) and ρ=0. Results are from $10^{5}$ simulation replicates. **Figure S14:** Disjunctive Probability of Success (dPoS) for θ=(0,0,θ) and ρ=0.25. Results are from $10^{5}$ simulation replicates. **Figure S15:** Disjunctive Probability of Success (dPoS) for θ=(0,0,θ) and ρ=0.5. Results are from $10^{5}$ simulation replicates. **Figure S16:** Disjunctive Probability of Success (dPoS) for θ=(0,0,θ) and ρ=0.75. Results are from $10^{5}$ simulation replicates. **Figure S17:** Disjunctive Probability of Success (dPoS) for θ=(θ/2,θ,2θ) and ρ=0. Results are from $10^{5}$ simulation replicates. **Figure S18:** Disjunctive Probability of Success (dPoS) for θ=(θ/2,θ,2θ) and ρ=0.25. Results are from $10^{5}$ simulation replicates. **Figure S19:** Disjunctive Probability of Success (dPoS) for θ=(θ/2,θ,2θ) and ρ=0.5. Results are from $10^{5}$ simulation replicates. **Figure S20:** Disjunctive Probability of Success (dPoS) for θ=(θ/2,θ,2θ) and ρ=0.75. Results are from $10^{5}$ simulation replicates. **Figure S21:** Marginal Probability of Success (mPoS) and mean empirical weights for $H_{1},H_{2},H_{3}$ for θ=(θ/2,θ,2θ) and ρ=0. Results are from $10^{5}$ simulation replicates. **Figure S22:** Marginal Probability of Success (mPoS) and mean empirical weights for $H_{1},H_{2},H_{3}$ for θ=(θ/2,θ,2θ) and ρ=0.25. Results are from $10^{5}$ simulation replicates. **Figure S23:** Marginal Probability of Success (mPoS) and mean empirical weights for $H_{1},H_{2},H_{3}$ for θ=(θ/2,θ,2θ) and ρ=0.5. Results are from $10^{5}$ simulation replicates. **Figure S24:** Marginal Probability of Success (mPoS) and mean empirical weights for $H_{1},H_{2},H_{3}$ for θ=(θ/2,θ,2θ) and ρ=0.75. Results are from $10^{5}$ simulation replicates. **Figure S25:** Probability of Success (PoS) for $H_{5}$ for θ=(0,0,0,0,θ) and ρ=0.25. Results are from $10^{5}$ simulation replicates. **Figure S26:** Probability of Success (PoS) for $H_{5}$ for θ=(0,0,0,0,θ) and ρ=0.25. Results are from $10^{5}$ simulation replicates. **Figure S27:** Probability of Success (PoS) for $H_{5}$ for θ=(0,0,0,0,θ) and ρ=0.5. Results are from $10^{5}$ simulation replicates. **Figure S28:** Probability of Success (PoS) for $H_{5}$ for θ=(0,0,0,0,θ) and ρ=0.75. Results are from $10^{5}$ simulation replicates. **Figure S29:** Disjunctive Probability of Success (dPoS) and marginal probability of success (mPoS) for $H_{5}$, for θ=(0,0,θ,θ,θ) and ρ=. Results are from $10^{5}$ simulation replicates. **Figure S30:** Disjunctive Probability of Success (dPoS) and marginal probability of success (mPoS) for $H_{5}$, for θ=(0,0,θ,θ,θ) and ρ=0.25. Results are from $10^{5}$ simulation replicates. **Figure S31:** Disjunctive Probability of Success (dPoS) and marginal probability of success (mPoS) for $H_{5}$, for θ=(0,0,θ,θ,θ) and ρ=0.5. Results are from $10^{5}$ simulation replicates. **Figure S32:** Disjunctive Probability of Success (dPoS) and marginal probability of success (mPoS) for $H_{5}$, for θ=(0,0,θ,θ,θ) and ρ=0.75. Results are from $10^{5}$ simulation replicates. **Figure S33:** Disjunctive Probability of Success (dPoS) for $\theta =(\theta_{1},\theta_{2},\theta_{3},\theta_{4},\theta )$ and marginal Probability of Success (mPoS) for $H_{5}$, with $\theta_{i}∼U[0,\theta ]$ independently for i=1,2,3,4 and ρ=0. Results are from $10^{5}$ simulation replicates. **Figure S34:** Disjunctive Probability of Success (dPoS) for $\theta =(\theta_{1},\theta_{2},\theta_{3},\theta_{4},\theta )$ and marginal Probability of Success (mPoS) for $H_{5}$, with $\theta_{i}∼U[0,\theta ]$ independently for i=1,2,3,4 and ρ=0.25. Results are from $10^{5}$ simulation replicates. **Figure S35:** Disjunctive Probability of Success (dPoS) for $\theta =(\theta_{1},\theta_{2},\theta_{3},\theta_{4},\theta )$ and marginal Probability of Success (mPoS) for $H_{5}$, with $\theta_{i}∼U[0,\theta ]$ independently for i=1,2,3,4 and ρ=0.5. Results are from $10^{5}$ simulation replicates. **Figure S36:** Disjunctive Probability of Success (dPoS) for $\theta =(\theta_{1},\theta_{2},\theta_{3},\theta_{4},\theta )$ and marginal Probability of Success (mPoS) for $H_{5}$, with $\theta_{i}∼U[0,\theta ]$ independently for i=1,2,3,4 and ρ=0.75. Results are from $10^{5}$ simulation replicates.

## Figures and Tables

**FIGURE C4:**
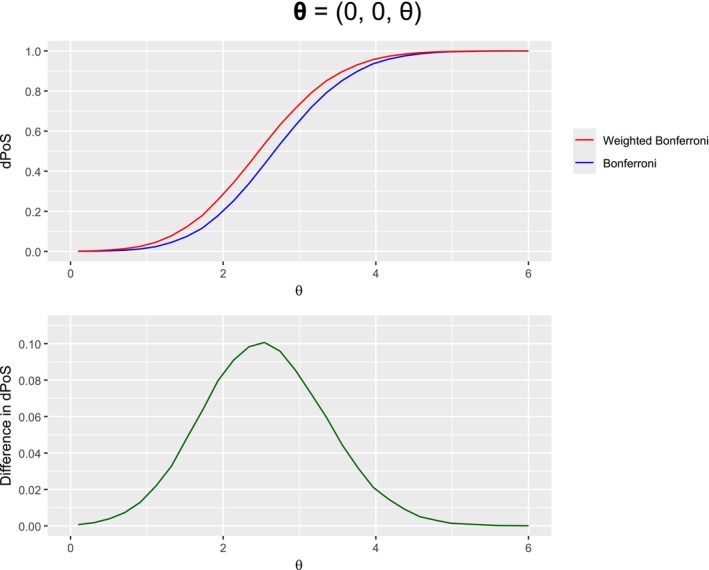
Disjunctive Probability of Success (dPoS) for θ=(0,0,θ). Results are from $10^{5}$ simulation replicates. Positive values of the difference in dPoS indicate where the dPoS of the weighted Bonferroni approach is higher than the unweighted Bonferroni approach.

## Data Availability

Code to reproduce the results given in Sections 3 and 4 can be found at https://github.com/dsrobertson/optimal_weighted_tests.

## Appendix A Proof of Form of Optimal Weights

To solve the optimization problem given in (1), we form the Lagrangian ℒ as follows:

$$\begin{array}{cc} ℒ & =1-\overset{m}{\underset{i=1}{\prod }}\left[\Phi \;\left({\bar{\Phi }}^{-1}(w_{i}\alpha )-\theta_{i}\right)\;1(\theta_{i}>0)\right]\\ & \;-\lambda \;\left(\;m-\overset{m}{\underset{i=1}{\sum }}w_{i}1(\theta_{i}>0)\;\right). \end{array}$$

Let ϕ denote the standard normal PDF (probability density function). Taking the (partial) derivative of ℒ with respect to $w_{j}$, where $\theta_{j}>0$ (we simply set $w_{j}=0$ otherwise) and equating this to zero gives

$$\begin{array}{ll} \frac{\partial ℒ}{\partial w_{j}} & =\lambda -\underset{i\in 𝒜,\;i\neq j}{\prod }\alpha \Phi \left({\bar{\Phi }}^{-1}(w_{i}\alpha )-\theta_{i}\right)1(\theta_{i}>0)\left[\frac{\phi \left({\bar{\Phi }}^{-1}(w_{j}\alpha )-\theta_{j}\right)}{\phi \left({\bar{\Phi }}^{-1}(w_{j}\alpha )\right)}\right]=0\\ & \;\Rightarrow \;\frac{\phi \left({\bar{\Phi }}^{-1}(w_{j}\alpha )-\theta_{j}\right)}{\phi \left({\bar{\Phi }}^{-1}(w_{j}\alpha )\right)}\\ & =\frac{\lambda }{\alpha }{\left[\underset{i\in 𝒜,\;i\neq j}{\prod }1(\theta_{i}>0)\;\Phi \left({\bar{\Phi }}^{-1}(w_{i}\alpha )-\theta_{i}\right)\right]}^{-1}\\ & \;\Rightarrow \;\exp\left(-\frac{\theta_{j}^{2}}{2}+\theta_{j}{\bar{\Phi }}^{-1}(w_{j}\alpha )\right)\\ & =\frac{\lambda }{\alpha }{\left[\underset{i\in 𝒜,\;i\neq j}{\prod }1(\theta_{i}>0)\;\Phi \left({\bar{\Phi }}^{-1}(w_{i}\alpha )-\theta_{i}\right)\right]}^{-1}\\ & \;\Rightarrow \;{\bar{\Phi }}^{-1}(w_{j}\alpha )=\frac{\theta_{j}}{2}+\frac{1}{\theta_{j}}\\ & \;\times \log\left[\frac{\lambda }{\alpha }{\left(\underset{i\in 𝒜,\;i\neq j}{\prod }1(\theta_{i}>0)\;\Phi \left({\bar{\Phi }}^{-1}(w_{i}\alpha )-\theta_{i}\right)\right)}^{-1}\;\right]\\ & \;\Rightarrow \;w_{j}=\frac{1}{\alpha }\;\bar{\Phi }\left(\frac{\theta_{j}}{2}+\frac{1}{\theta_{j}}\left[\log(c)-\underset{i\in 𝒜,\;i\neq j}{\sum }1(\theta_{i}>0)\right.\right.\\ & \;\times \left.\left.\log\left(\Phi ({\bar{\Phi }}^{-1}(w_{i}\alpha )-\theta_{i})\right)\right]\right) \end{array}$$

where $c=\frac{\lambda }{\alpha }$.

## Appendix B Comparison of Computational Methods

In this Section, we compare the following numerical methods for finding optimal weights:

1. nleq = use of nonlinear simultaneous equations solver (nleqslv package in R), as proposed and used in this article.
2. nlopt = use of nonlinear optimization algorithm with constraints (nloptr package in R) and a single starting point, as proposed by [16]
3. nlopt multi = use of nonlinear optimization algorithm with constraints (nloptr package in R) and multiple starting points, as proposed by [16]
4. grid search

We use the above numerical methods to find optimal weights for m treatments, m∈{2,3,4,5}, where the true treatment means are given by $\theta =(\theta_{1},\ldots ,\theta_{m})$ with $\theta_{i}∼U[0,b]$ independently for i=1,…,m. Here the upper bound b=6 for m=2 and b=3 otherwise. We generate 1000 such vectors of treatment means for each value of m.

Table B1 shows the time taken for each method to calculate the optimal weights. The nlopt method is the fastest, but as we shall see comes at the cost of sub‐optimal weights. The nleq method is about two times slower than nlopt. Finally, the nlopt multi method is substantially slower than either nleq or nlopt, particularly when m>2, which is to be expected given that $2^{m}-1$ starting points need to be considered for this method.

**TABLE B1:** Timings in seconds to calculate optimal weights for 1000 sets of uniform random treatment effect vectors.

Method	Two treatments	Three treatments	Four treatments	Five treatments
nleq	2.2	4.7	8.9	15.8
nlopt	1.5	2.5	4.0	6.3
nlopt multi	4.6	28.4	119.9	472.5
grid search	1.3	338.8	—	—

*Note:* For grid search, the grid size was set equal to 0.005.

Table B2 compares the optimal weights and resulting disjunctive power found using nleq, nlopt and nlopt multi with those found using grid search for m=2,3. The nleq method always finds the same optimal weights as those found using grid search (rounding to the grid size) and always has the greater or equal disjunctive power. In contrast, nlopt and nlopt multi find suboptimal weights (compared with those found using grid search) for a noticeable proportion of treatment effect vectors, particularly for m=2.

**TABLE B2:** Comparison of different methods compared with grid search, in terms of percentage of optimal weights which are within rounding error of the grid search weights (“≈ equal” column), sum of Euclidean distance with the grid search weights, and percentage of realized disjunctive power that is equal or greater than the disjunctive power using the grid search weights (“≥ column”).

Method	Two treatments			Three treatments		
	≈ weights	Euclidean distance	≥ power	≈ weights	Euclidean distance	≥ power
nleq	100%	1.3	100%	100%	2.0	100%
nlopt	86.8%	20.1	84.3%	93.5%	7.9	90.3%
nlopt multi	87.3%	11.9	85.3%	99.9%	2.1	98.3%

*Note:* Results are based on 1000 sets of uniform random treatment effect vectors. For grid search, the grid size was set equal to 0.005.

The absolute magnitude of the power differences is small, however. The maximum power loss compared to using grid search for m=2 was 0.0039% and 0.0011% (in absolute terms) for nlopt and nlopt multi, respectively. For m=3, the maximum power loss was 0.51% and 0.0019% (in absolute terms) for nlopt and nlopt multi, respectively.

Finally, we compare the realized disjunctive power using nlopt and nlopt multi with that found using nleq in Table B3. Note that nleq always had greater or equal disjunctive power compared with nlopt and nlopt multi. Table B3 shows that for a substantial proportion of treatment effect vectors, both nlopt and nlopt multi lead to suboptimal weights and a lower disjunctive power compared with nleq. This is particularly the case for nlopt and for smaller values of m.

**TABLE B3:** Percentage of realized disjunctive powers that are equal to the disjunctive power using the nleq optimal weights.

Method	Two treatments	Three treatments	Four treatments	Five treatments
nlopt	74.3%	74.6%	66.4%	59.9%
nlopt multi	76.2%	85.4%	85.9%	90.7%

*Note:* There were no cases were using the nleq resulted in a lower disjunctive power. Results are based on 1000 sets of uniform random treatment effect vectors.

Again, the absolute magnitude of the power differences is small. The maximum power loss for nlopt compared to using nleq was 0.0040%, 0.51%, 0.35% and 0.28% (in absolute terms) for m=2,3,4,5, respectively. The maximum power loss for nlopt multi compared to using nleq was 0.0012%, 0.0019%, 0.0099% and 0.0013% (in absolute terms) for m=2,3,4,5, respectively.

## Appendix C Additional Simulation Results

### Consistent Treatment Effects

#### Two Treatments

Figure C1 shows the dPoS (which is the same as the mPoS for $H_{2}$) under the scenario where θ=(0,θ).

Figure C2 shows the dPoS for θ=(θ/2,θ), as well as the mPoS for $H_{1}$ and $H_{2}$.

Table C1 shows the detailed results for rejection and non‐rejection for the two hypothesis for θ=(0,3) based on 1000 simulations.

**TABLE C1:** Overall rejections of $H_{1}$ and $H_{2}$ when using the weighted and unweighted Bonferroni procedures for θ=(0,3).

Test		Weighted	
		Rejection	No rejection
*H*1			
Unweighted	Rejection	0	1
	No rejection	0	999
*H*2			
Unweighted	Rejection	715	0
	No rejection	50	235

*Note:* Results are from 1000 simulation replicates.

Now assume that the true standardized treatment means are given by θ=(θ,θ), where θ>0. Figure C3 shows the dPoS and mPoS for $H_{1}$ and $H_{2}$.

Table C2 shows the detailed results for rejection and non‐rejection for the two hypothesis for θ=(3,3) based on 1000 simulations.

**TABLE C2:** Overall rejections of $H_{1}$ and $H_{2}$ when using the weighted and unweighted Bonferroni procedures for θ=(3,3).

Test		Weighted	
		Rejection	No rejection
*H*1			
Unweighted	Rejection	741	0
	No rejection	35	224
*H*2			
Unweighted	Rejection	713	2
	No rejection	44	241

*Note:* Results are from $10^{3}$ simulation replicates.

#### m>2 Treatments

Figure C4 shows the dPoS (which is the same as the mPoS for $H_{3}$) when θ=(0,0,θ).

Figure C5 shows the dPoS for θ=(θ/2,θ,2θ).

Figure C6 shows the (marginal = disjunctive) PoS for θ=(0,0,0,0,θ).

Figure C7 shows the dPoS for $\theta =(\theta_{1},\theta_{2},\theta_{3},\theta_{4},\theta )$, with $\theta_{i}∼U[0,\theta ]$ independently for i=1,2,3,4 and θ>0, as well as the mPoS for $H_{5}$.
