# Supplementary material for: Optimal Weighted Tests for Replication Studies and the ‘Two‐Trials Rule’ With Multiple Hypotheses
Source: Stat Med. 2026 Jul 1;45(15-17):e70638. doi: 10.1002/sim.70638 (PMC13323599; doi:10.1002/sim.70638)
Supplement: Supplementary file 1 — Data S1: Figure S1: PoS for H2 for θ=(0,θ) and ρ=0. Results are from 105 simulation replicates. Figure S2: PoS for H2 for θ=(0,θ) and ρ=0.25. Results are from 105 simulation replicates. Figure S3: PoS for H2 for θ=(0,θ) and ρ=0.5. Results are from 105 simulation replicates. Figure S4: PoS for H2 for θ=(0,θ) and ρ=0.75. Results are from 105 simulation replicates. Figure S5: Disjunctive Probability of Success (dPoS) for and marginal Probability of Success (mPoS) for H1 and H2, for θ=(θ/2,θ) and ρ=0. Results are from 105 simulation replicates. Figure S6: Disjunctive Probability of Success (dPoS) for and marginal Probability of Success (mPoS) for H1 and H2, for θ=(θ/2,θ) and ρ=0.25. Results are from 105 simulation replicates. Figure S7: Disjunctive Probability of Success (dPoS) for and marginal Probability of Success (mPoS) for H1 and H2, for θ=(θ/2,θ) and ρ=0.5. Results are from 105 simulation replicates. Figure S8: Disjunctive Probability of Success (dPoS) for and marginal Probability of Success (mPoS) for H1 and H2, for θ=(θ/2,θ) and ρ=0.75. Results are from 105 simulation replicates. Figure S9: Disjunctive Probability of Success (dPoS) and marginal Probability of Success (mPoS) for H1 and H2, for θ=(θ,θ) and ρ=0. Results are from 105 simulation replicates. Figure S10: Disjunctive Probability of Success (dPoS) and marginal Probability of Success (mPoS) for H1 and H2, for θ=(θ,θ) and ρ=0.25. Results are from 105 simulation replicates. Figure S11: Disjunctive Probability of Success (dPoS) and marginal Probability of Success (mPoS) for H1 and H2, for θ=(θ,θ) and ρ=0.5. Results are from 105 simulation replicates. Figure S12: Disjunctive Probability of Success (dPoS) and marginal Probability of Success (mPoS) for H1 and H2, for θ=(θ,θ) and ρ=0.75. Results are from 105 simulation replicates. Figure S13: Disjunctive Probability of Success (dPoS) for θ=(0,0,θ) and ρ=0. Results are from 105 simulation replicates. Figure S14: Disjunctive Probability of Success (dPoS) for θ=(0, [file SIM-45-0-s001.pdf]

Supplementary Material for "Optimal weighted tests for replication studies and the 'two-trials rule' with multiple hypotheses"

David S. Robertson*[a] and Thomas Jaki[a,b]

[a]MRC Biostatistics Unit, University of Cambridge, UK
[b]University of Regensburg, Germany

1 Robustness to the independence assumption

We present simulation results showing the performance of the weighted and unweighted Bonferroni under different correlation structures. We assume that $(T_1^{(j)}, \cdots, T_m^{(j)})$ follow a multivariate normal distribution for $j = 1, 2$, with mean vector $(\theta_1, \ldots, \theta_m)$ and covariance matrix Σ where

$$\Sigma_{i,k} = \begin{cases} 1 & \text{if } i = k \\ \rho & \text{if } i \neq k \end{cases}$$

We vary $\rho \in \{0, 0.25, 0.5, 0.75\}$.

1.1 Results for $\boldsymbol{\theta} = (0, \theta)$

Figures 1, 2, 3 and 4 show the disjunctive PoS (which is the same as the marginal PoS for H_2) when $\rho = 0, 0.25, 0.5, 0.75$, respectively.

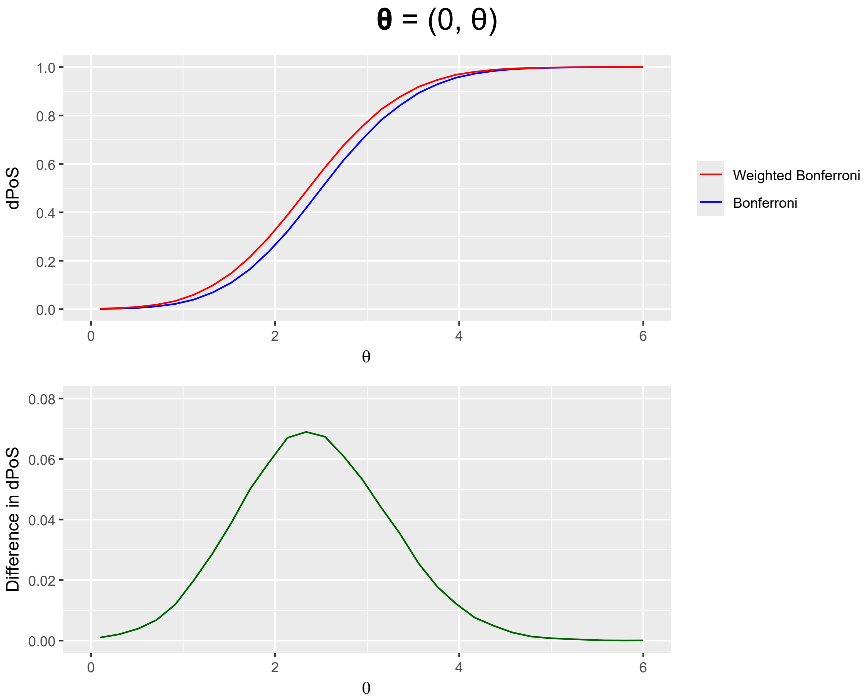

Figure 1: PoS for H_2 for $\boldsymbol{\theta} = (0, \theta)$ and $\rho = 0$. Results are from 10^5 simulation replicates.

*Corresponding author david.robertson@mrc-bsu.cam.ac.uk

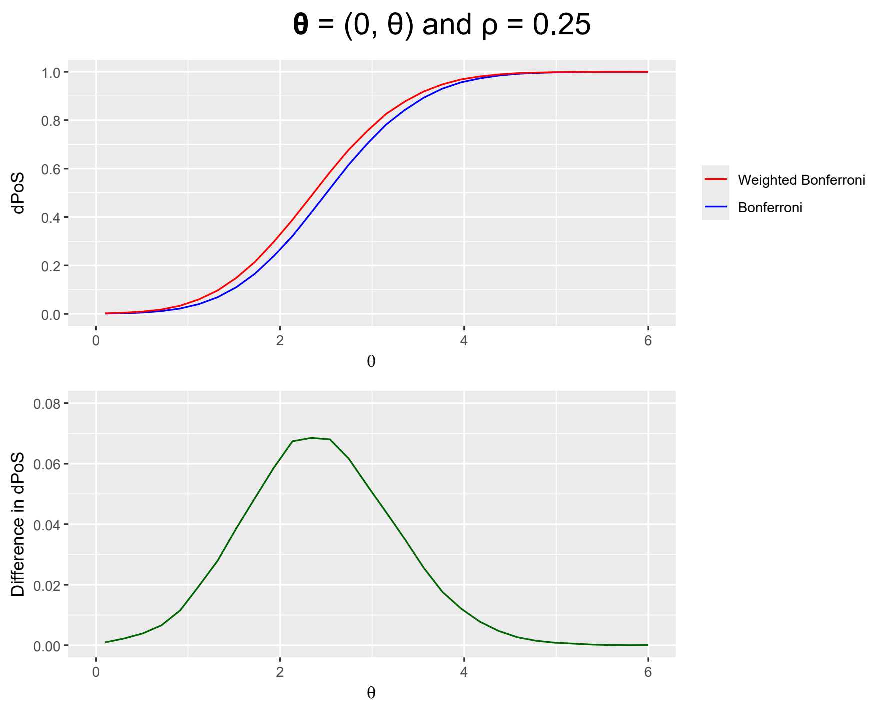

Figure 2: PoS for H_2 for $\boldsymbol{\theta} = (0, \theta)$ and $\rho = 0.25$. Results are from 10^5 simulation replicates.

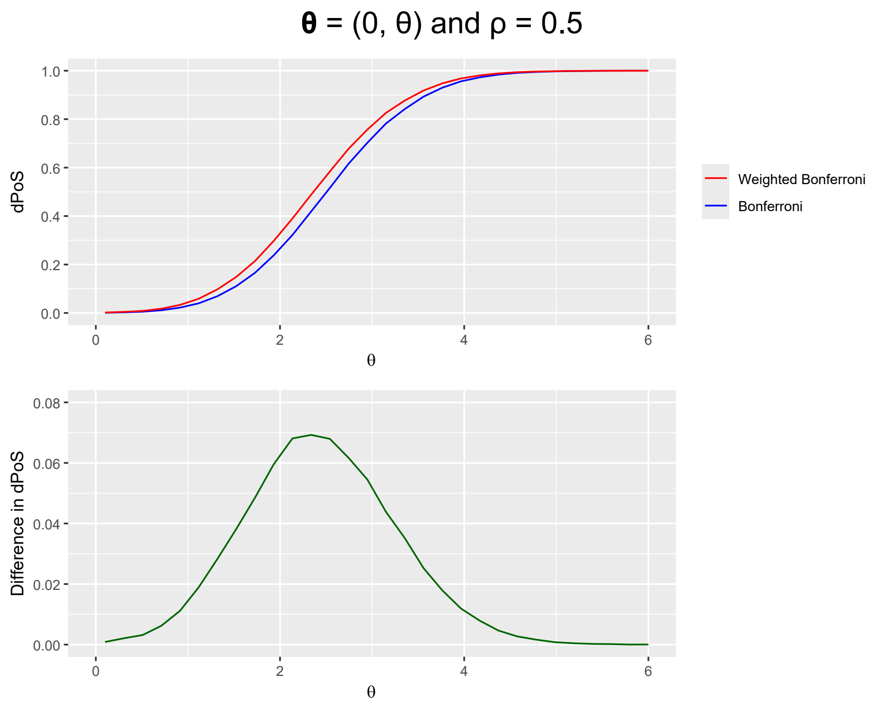

Figure 3: PoS for H_2 for $\boldsymbol{\theta} = (0, \theta)$ and $\rho = 0.5$. Results are from 10^5 simulation replicates.

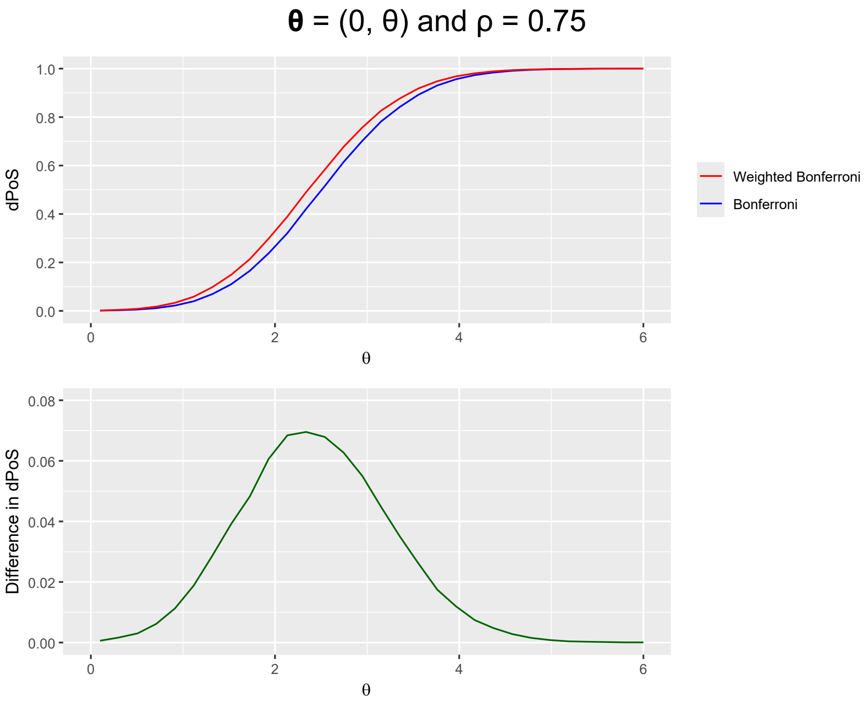

Figure 4: PoS for H_2 for $\boldsymbol{\theta} = (0, \theta)$ and $\rho = 0.75$. Results are from 10^5 simulation replicates.

1.2 Results for $\boldsymbol{\theta} = (\theta/2, \theta)$

Figures 5, 6, 7 and 8 show the dPoS as well as the mPoS for H_1 and H_2, for $\rho = 0, 0.25, 0.5, 0.75$, respectively.

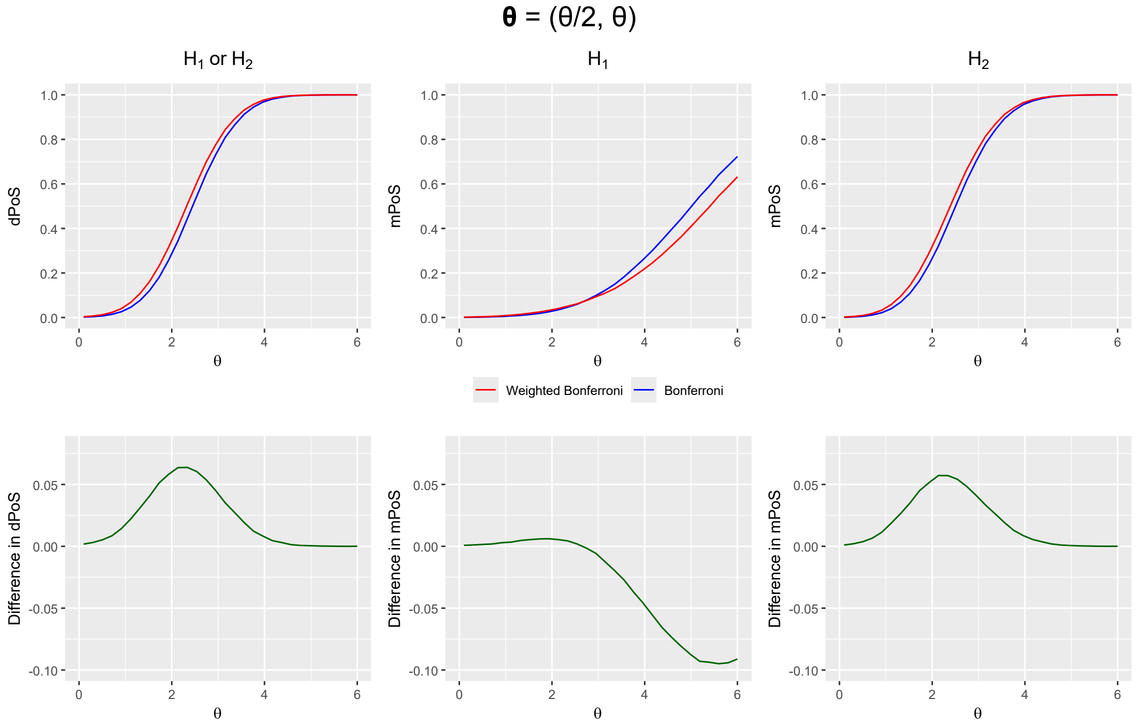

Figure 5: Disjunctive Probability of Success (dPoS) for and marginal Probability of Success (mPoS) for H_1 and H_2, for $\boldsymbol{\theta} = (\theta/2, \theta)$ and $\rho = 0$. Results are from 10^5 simulation replicates.

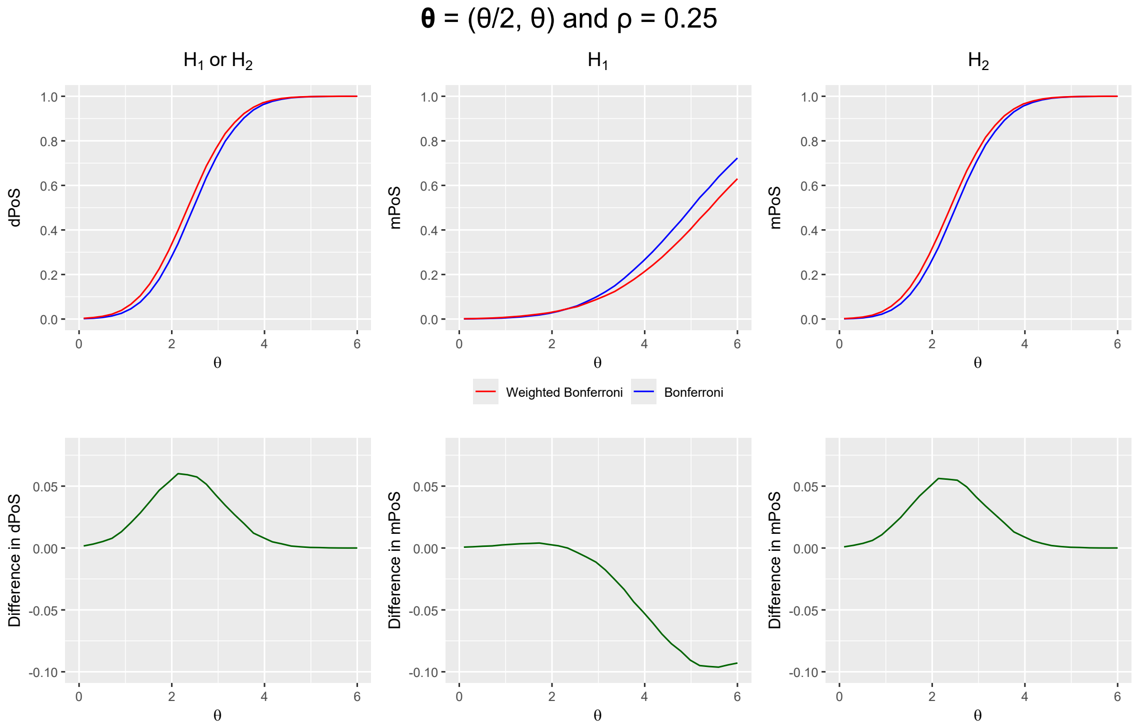

Figure 6: Disjunctive Probability of Success (dPoS) for and marginal Probability of Success (mPoS) for H_1 and H_2, for $\boldsymbol{\theta} = (\theta/2, \theta)$ and $\rho = 0.25$. Results are from 10^5 simulation replicates.

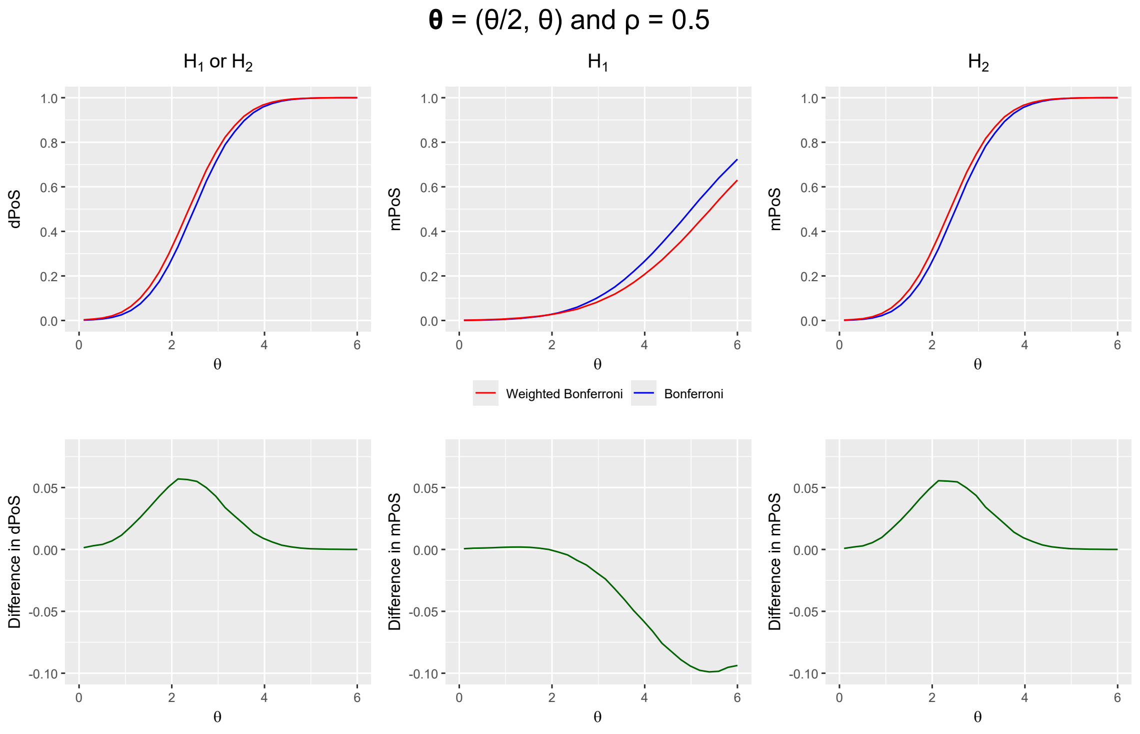

Figure 7: Disjunctive Probability of Success (dPoS) for and marginal Probability of Success (mPoS) for H_1 and H_2, for $\boldsymbol{\theta} = (\theta/2, \theta)$ and $\rho = 0.5$. Results are from 10^5 simulation replicates.

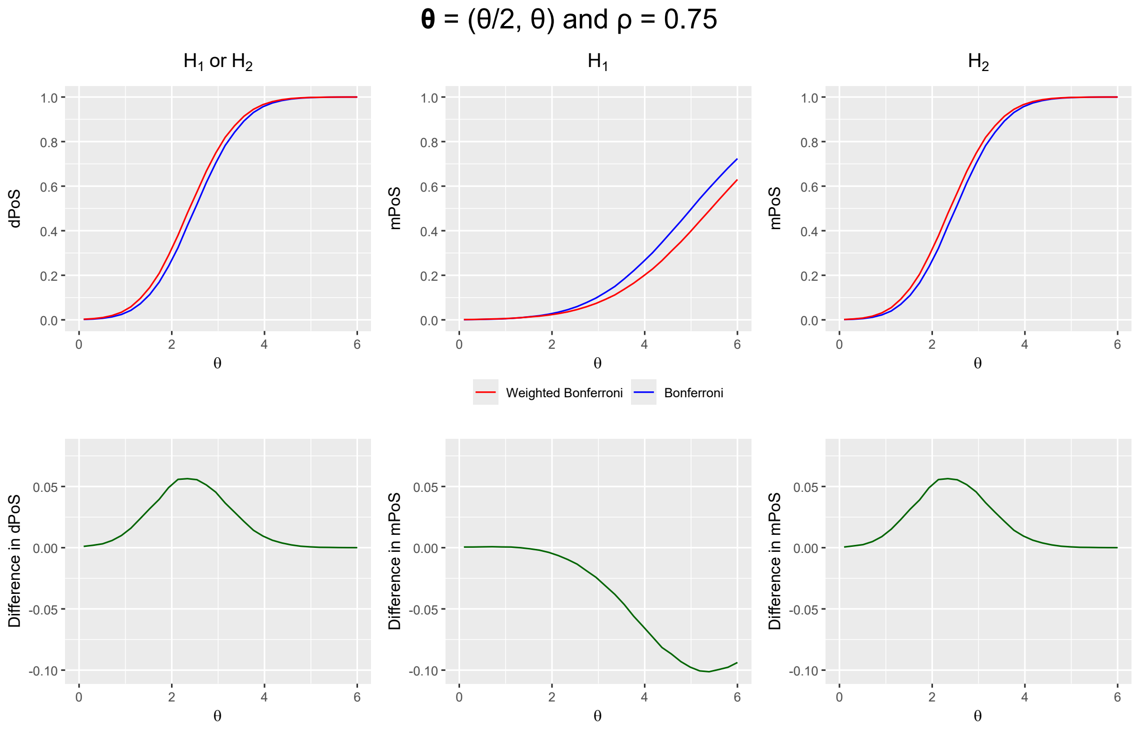

Figure 8: Disjunctive Probability of Success (dPoS) for and marginal Probability of Success (mPoS) for H_1 and H_2, for $\boldsymbol{\theta} = (\theta/2, \theta)$ and $\rho = 0.75$. Results are from 10^5 simulation replicates.

1.3 Results for $\boldsymbol{\theta} = (\theta, \theta)$

Figures 9, 10, 11 and 12 show the dPoS, as well as the mPoS for H_1 and H_2 when $\boldsymbol{\theta} = (\theta, \theta)$, for $\rho = 0, 0.25, 0.5, 0.75$, respectively.

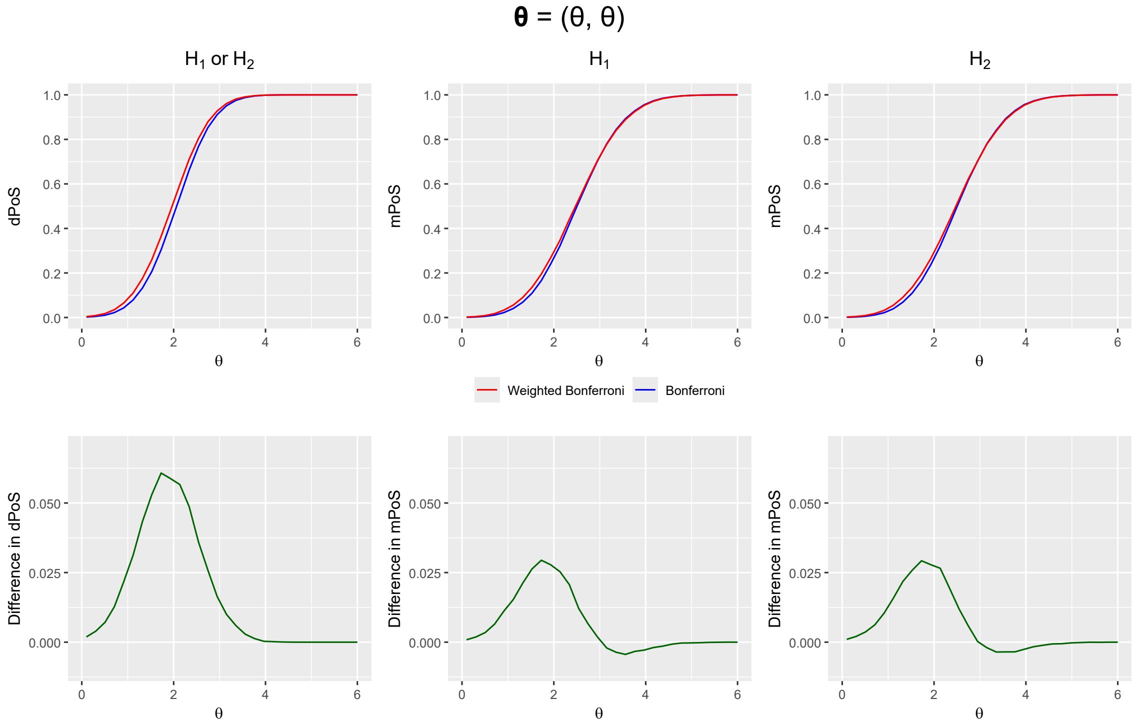

Figure 9: Disjunctive Probability of Success (dPoS) and marginal Probability of Success (mPoS) for H_1 and H_2, for $\boldsymbol{\theta} = (\theta, \theta)$ and $\rho = 0$. Results are from 10^5 simulation replicates.

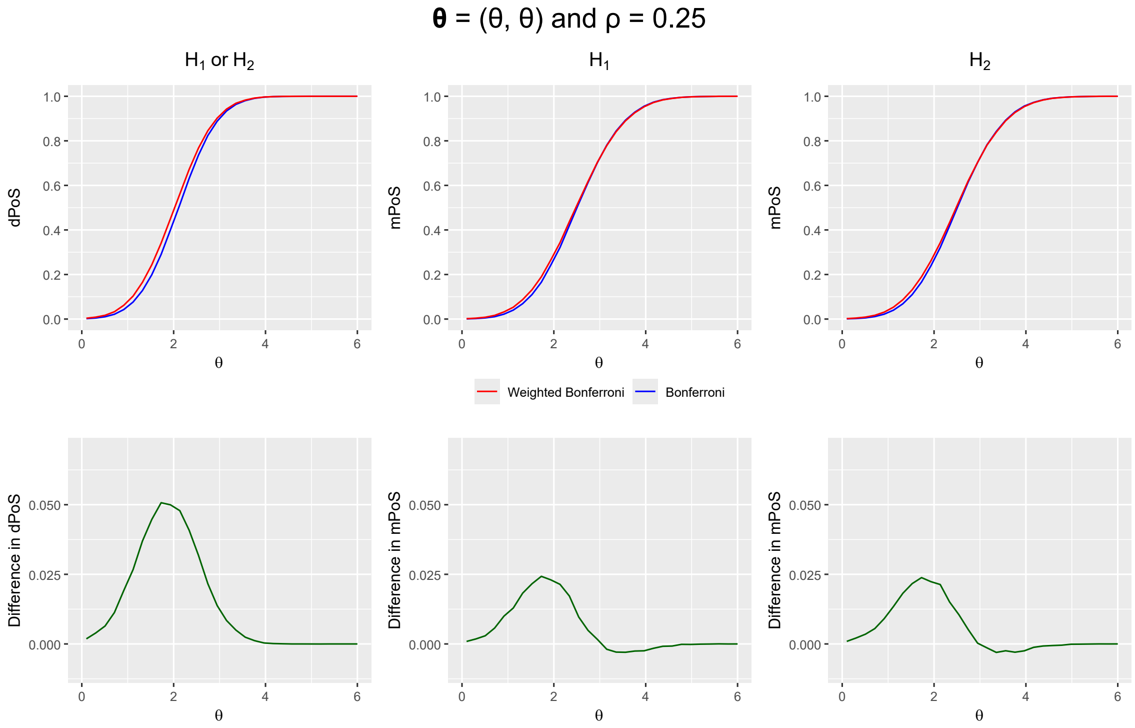

Figure 10: Disjunctive Probability of Success (dPoS) and marginal Probability of Success (mPoS) for H_1 and H_2, for $\boldsymbol{\theta} = (\theta, \theta)$ and $\rho = 0.25$. Results are from 10^5 simulation replicates.

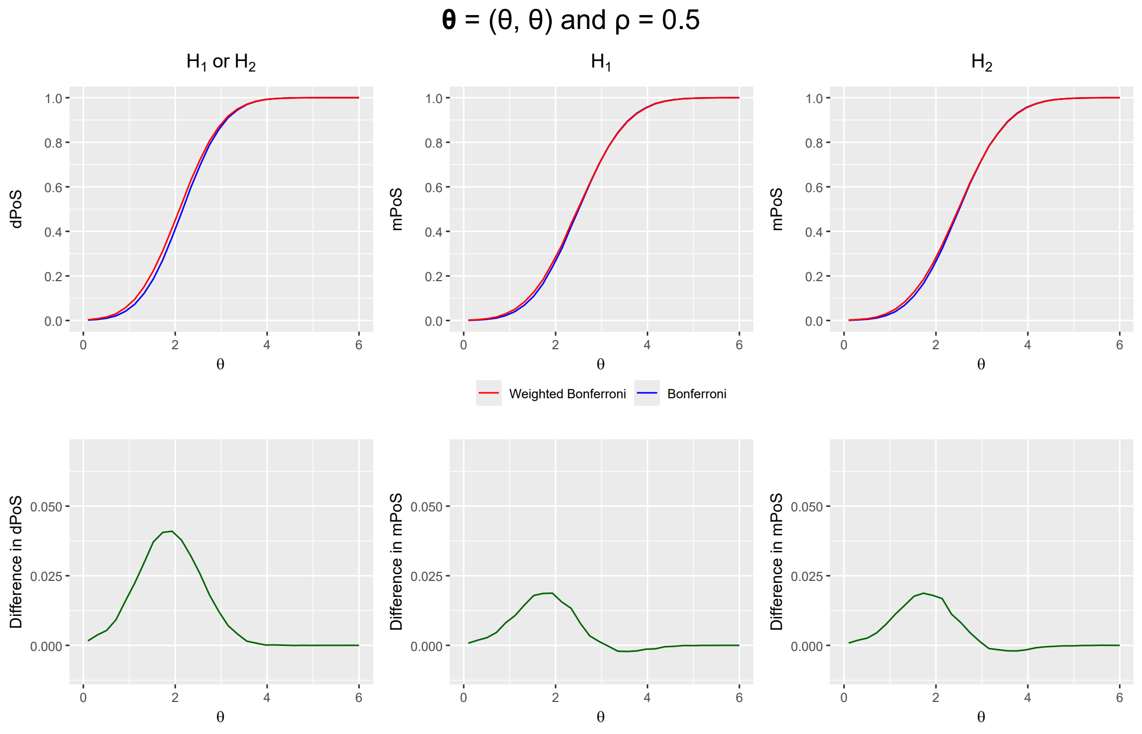

Figure 11: Disjunctive Probability of Success (dPoS) and marginal Probability of Success (mPoS) for H_1 and H_2, for $\boldsymbol{\theta} = (\theta, \theta)$ and $\rho = 0.5$. Results are from 10^5 simulation replicates.

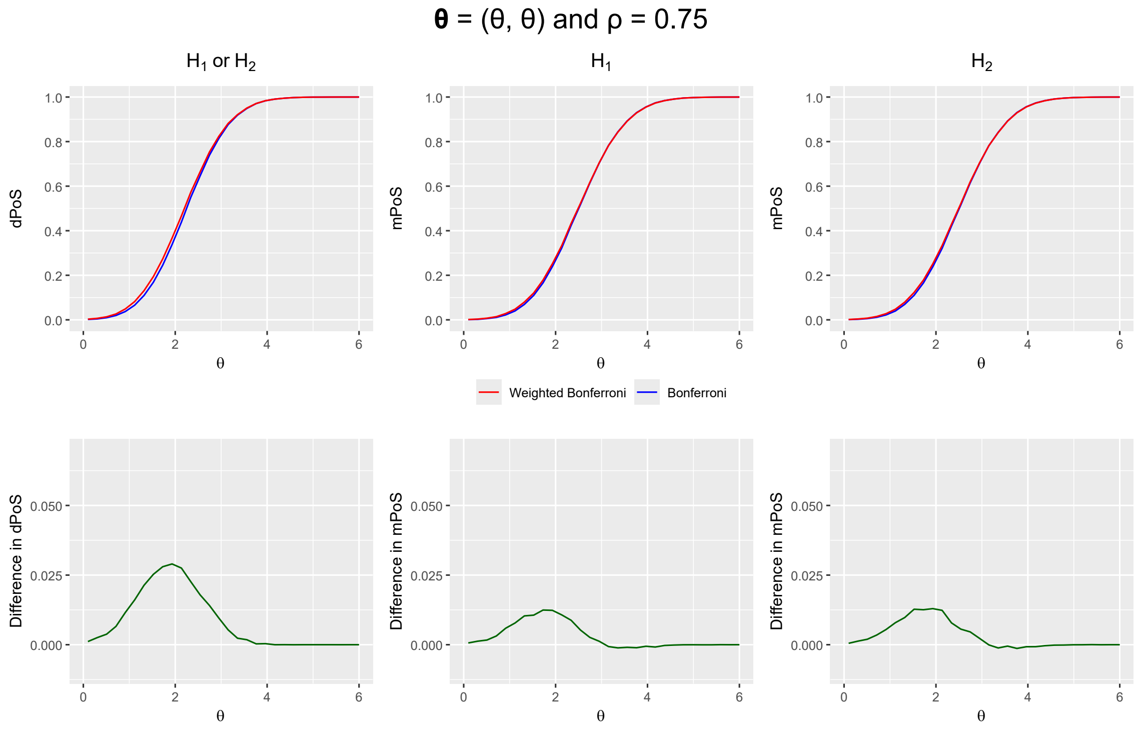

Figure 12: Disjunctive Probability of Success (dPoS) and marginal Probability of Success (mPoS) for H_1 and H_2, for $\boldsymbol{\theta} = (\theta, \theta)$ and $\rho = 0.75$. Results are from 10^5 simulation replicates.

1.4 Results for $\boldsymbol{\theta} = (0, 0, \theta)$

Figures 13, 14 shows the dPoS (which is the same as the mPoS for H_3) when $\boldsymbol{\theta} = (0, 0, \theta)$, for $\rho = 0, 0.25, 0.5, 0.75$, respectively.

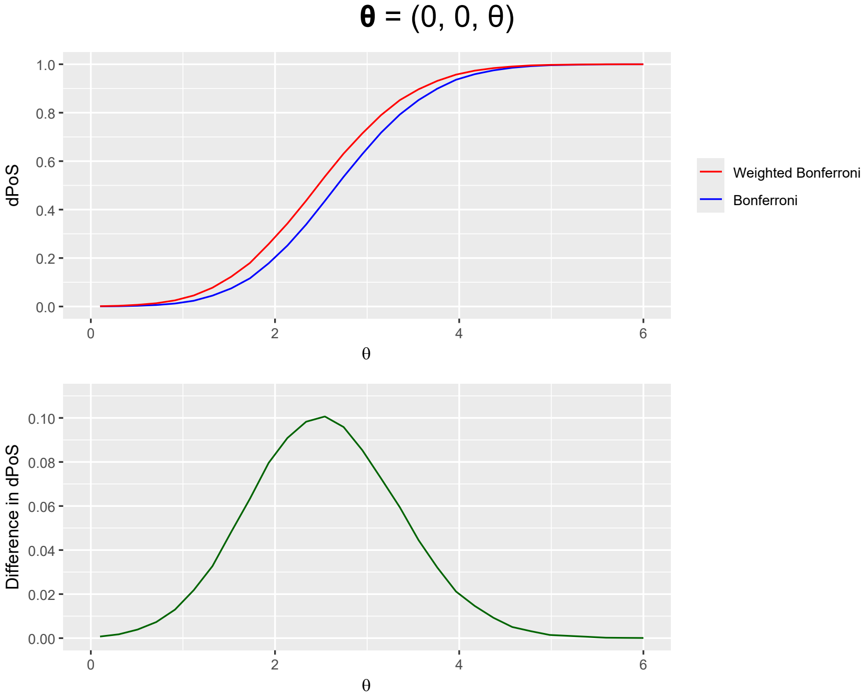

Figure 13: Disjunctive Probability of Success (dPoS) for $\boldsymbol{\theta} = (0, 0, \theta)$ and $\rho = 0$. Results are from 10^5 simulation replicates.

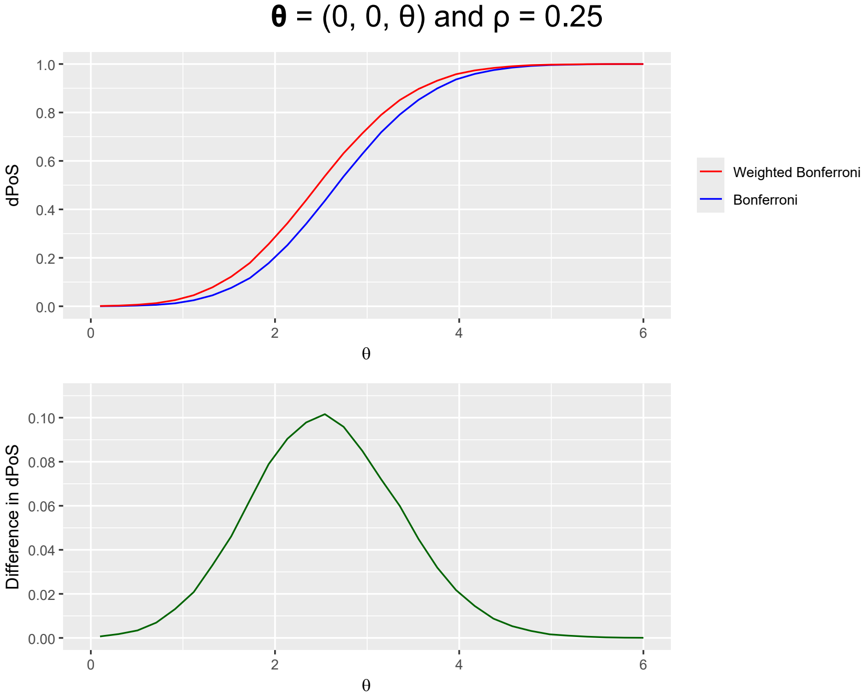

Figure 14: Disjunctive Probability of Success (dPoS) for $\boldsymbol{\theta} = (0, 0, \theta)$ and $\rho = 0.25$. Results are from 10^5 simulation replicates.

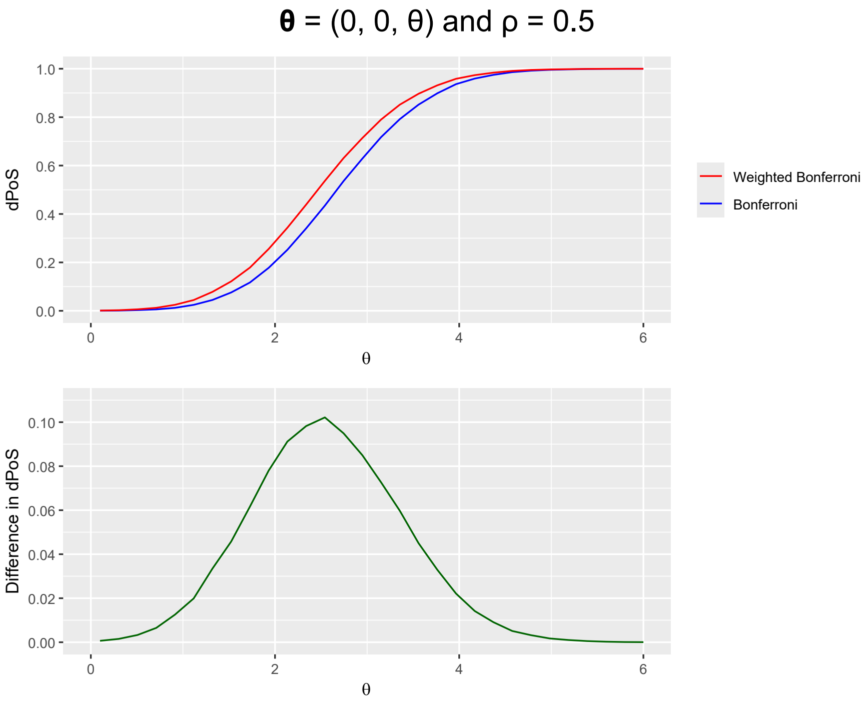

Figure 15: Disjunctive Probability of Success (dPoS) for $\boldsymbol{\theta} = (0, 0, \theta)$ and $\rho = 0.5$. Results are from 10^5 simulation replicates.

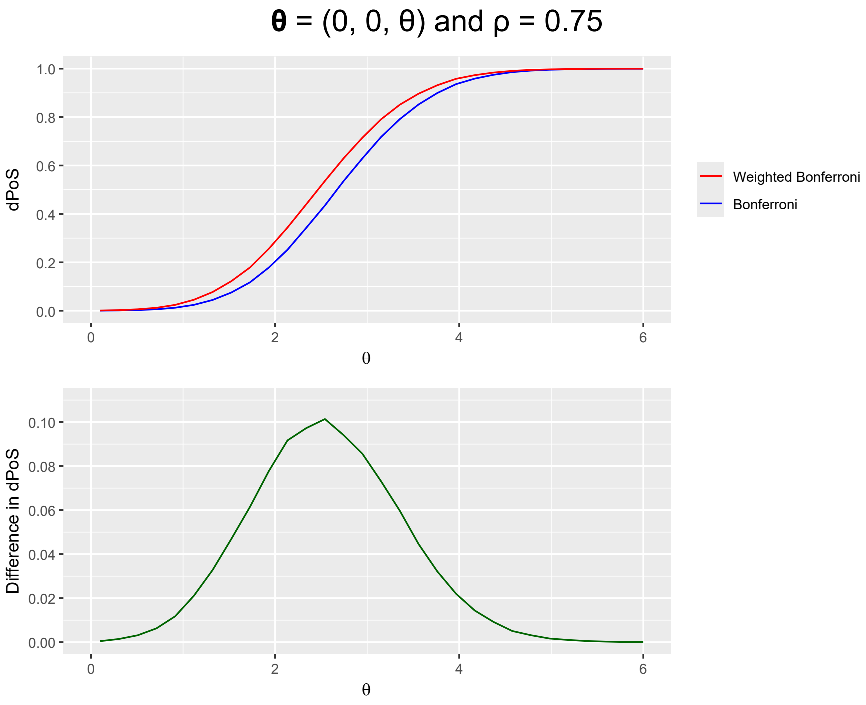

Figure 16: Disjunctive Probability of Success (dPoS) for $\boldsymbol{\theta} = (0, 0, \theta)$ and $\rho = 0.75$. Results are from 10^5 simulation replicates.

1.5 Results for $\boldsymbol{\theta} = (\theta/2, \theta, 2\theta)$

Figures 17, 18, 19 and 20 show the dPoS when $\boldsymbol{\theta} = (\theta/2, \theta, 2\theta)$, for $\rho = 0, 0.25, 0.5, 0.75$, respectively.

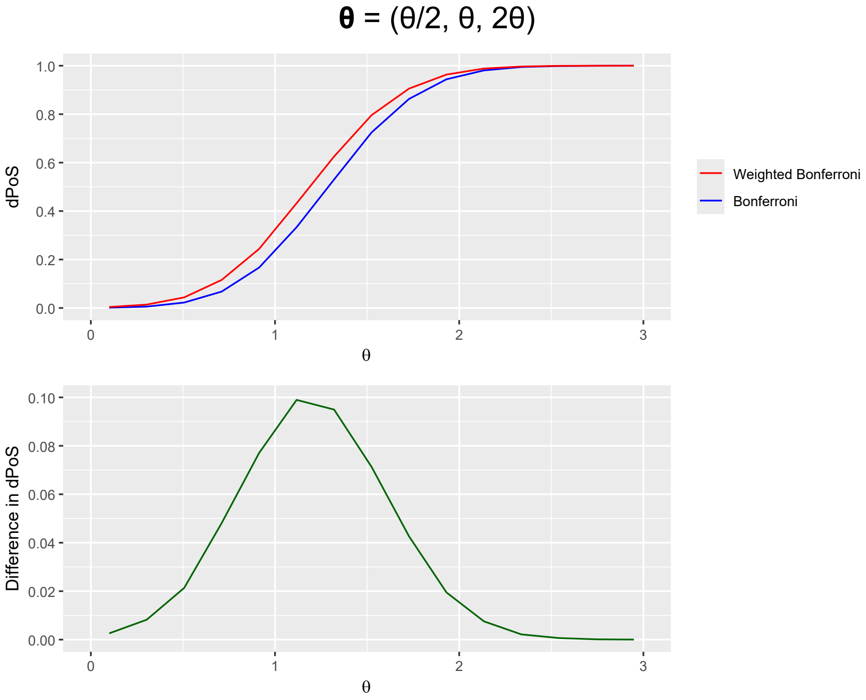

Figure 17: Disjunctive Probability of Success (dPoS) for $\boldsymbol{\theta} = (\theta/2, \theta, 2\theta)$ and $\rho = 0$. Results are from 10^5 simulation replicates.

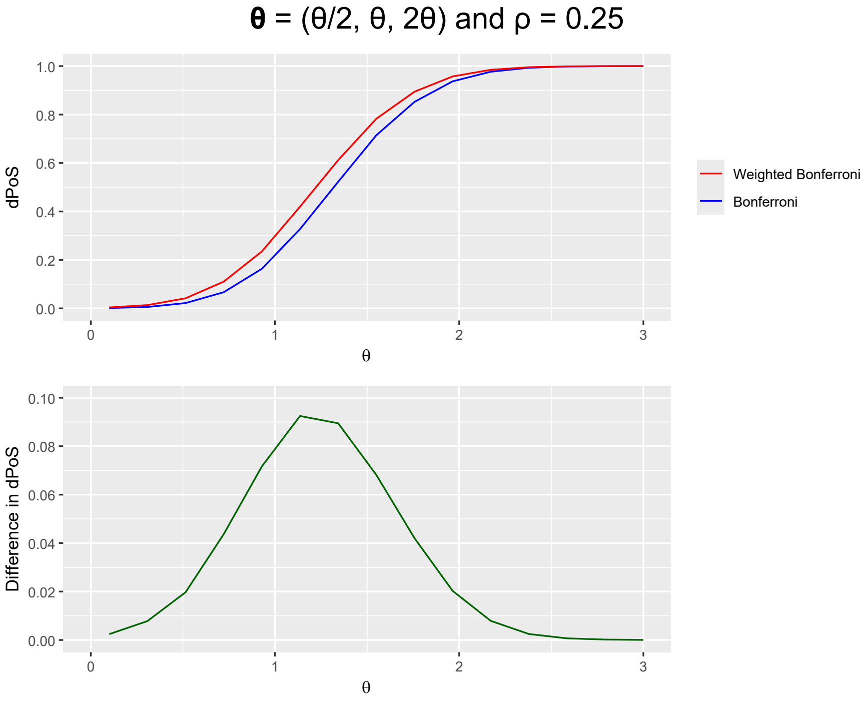

Figure 18: Disjunctive Probability of Success (dPoS) for $\boldsymbol{\theta} = (\theta/2, \theta, 2\theta)$ and $\rho = 0.25$. Results are from 10^5 simulation replicates.

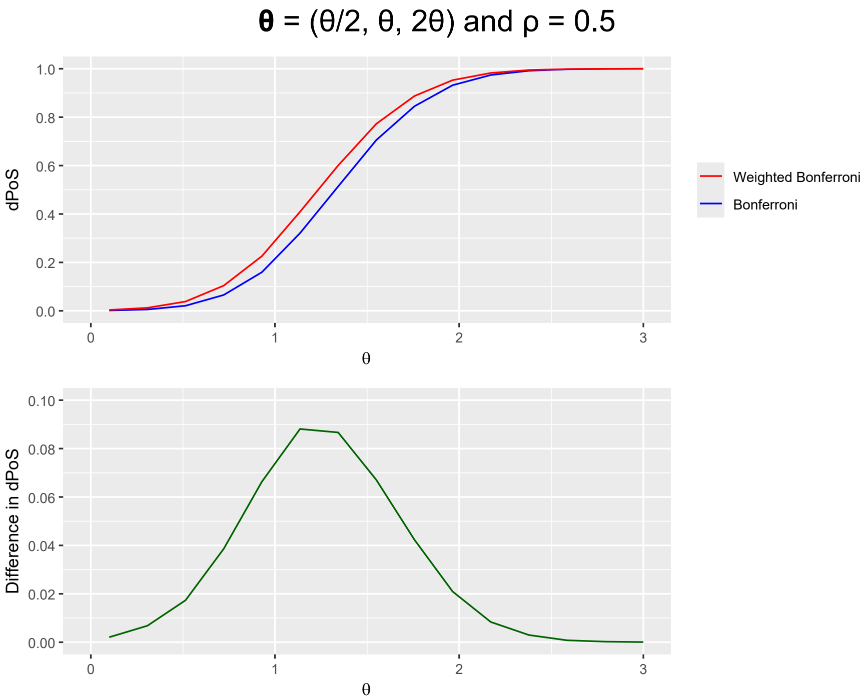

Figure 19: Disjunctive Probability of Success (dPoS) for $\boldsymbol{\theta} = (\theta/2, \theta, 2\theta)$ and $\rho = 0.5$. Results are from 10^5 simulation replicates.

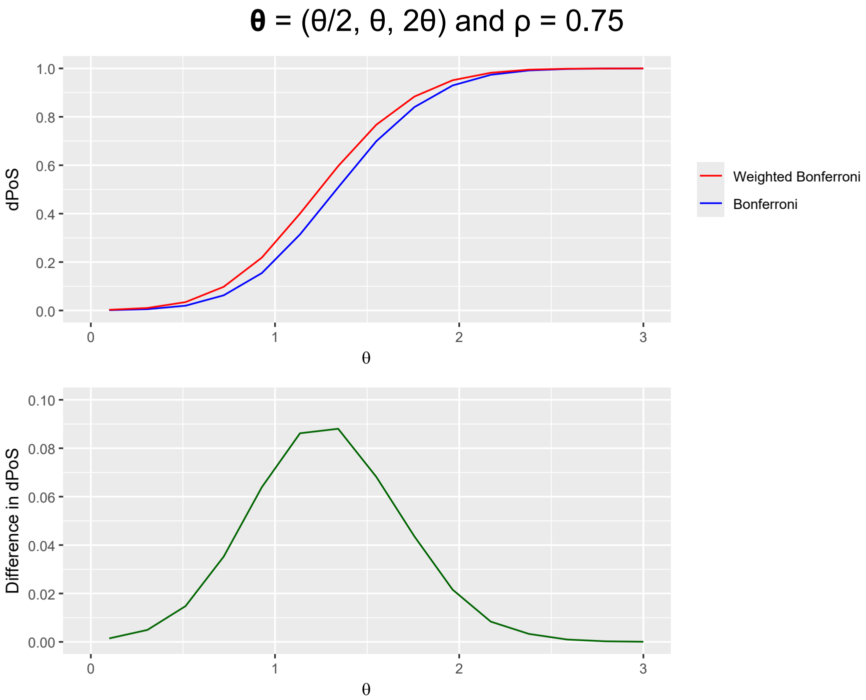

Figure 20: Disjunctive Probability of Success (dPoS) for $\boldsymbol{\theta} = (\theta/2, \theta, 2\theta)$ and $\rho = 0.75$. Results are from 10^5 simulation replicates.

Figures 21, 22, 23 and 24 show the mPoS for H_1, H_2 and H_3 as well as the mean (empirical) weights $\hat{w}_1, \hat{w}_2, \hat{w}_3$ when $\boldsymbol{\theta} = (\theta/2, \theta, 2\theta)$, for $\rho = 0, 0.25, 0.5, 0.75$, respectively.

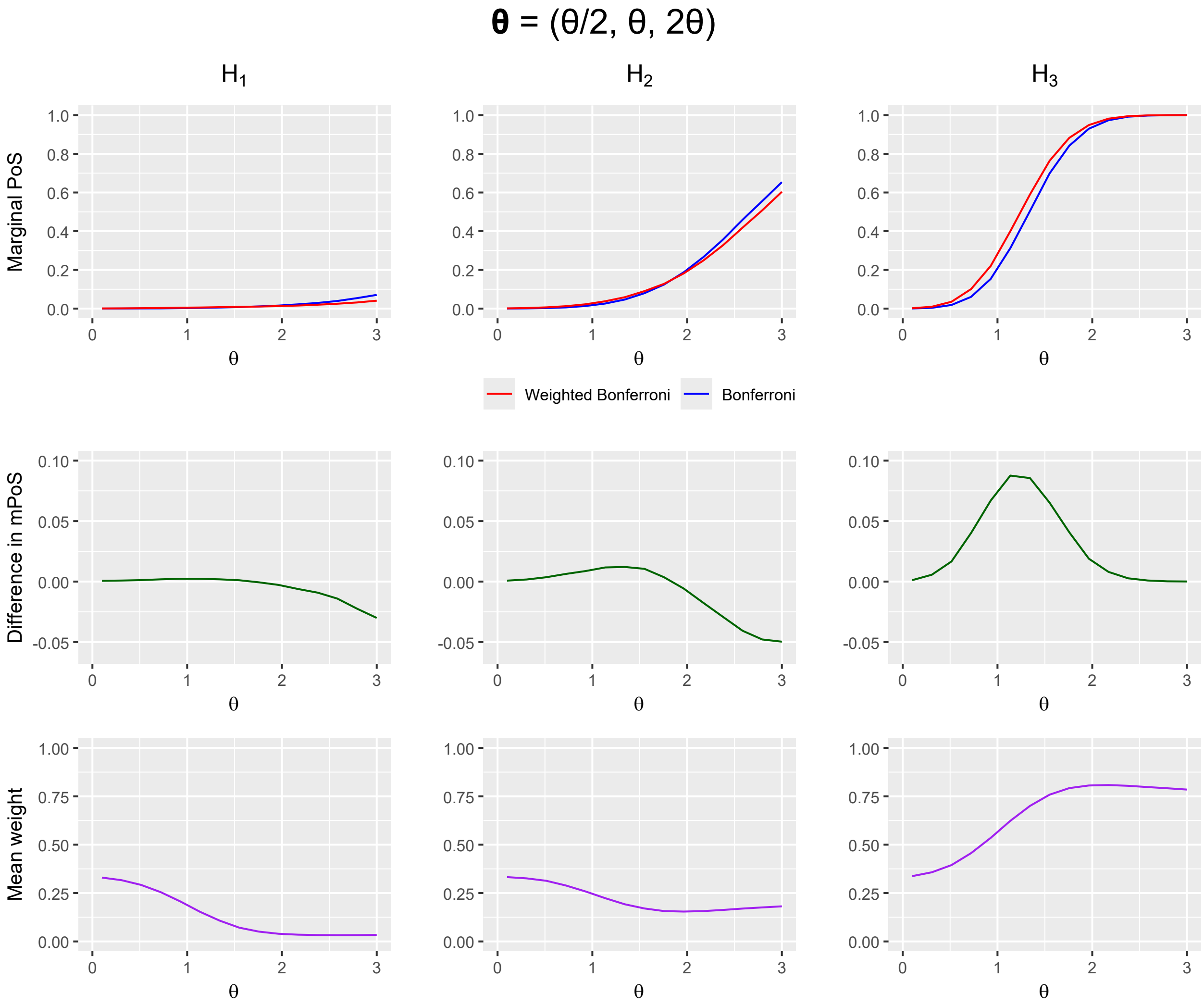

Figure 21: Marginal Probability of Success (mPoS) and mean empirical weights for H_1, H_2, H_3 for $\boldsymbol{\theta} = (\theta/2, \theta, 2\theta)$ and $\rho = 0$. Results are from 10^5 simulation replicates.

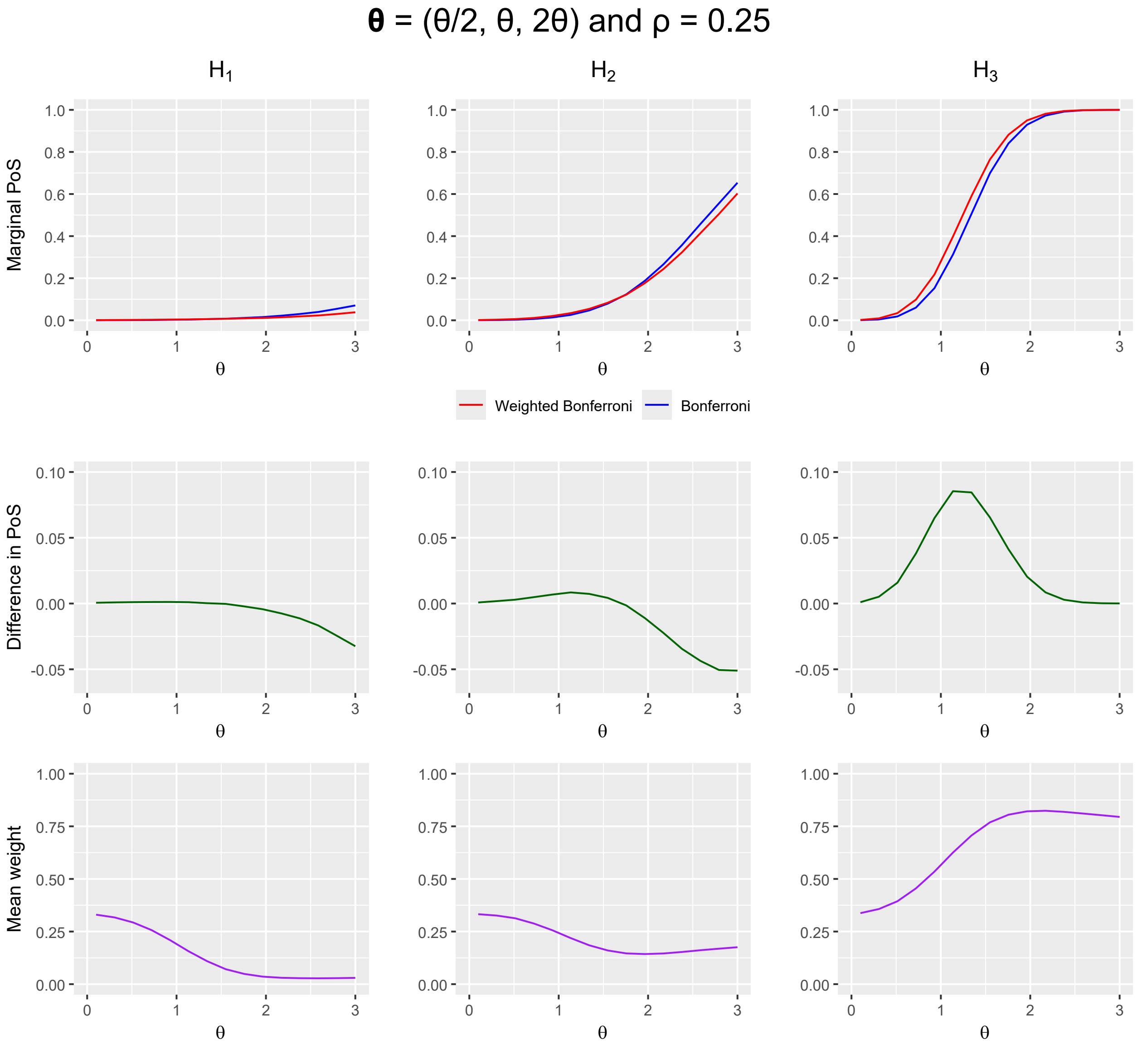

Figure 22: Marginal Probability of Success (mPoS) and mean empirical weights for H_1, H_2, H_3 for $\boldsymbol{\theta} = (\theta/2, \theta, 2\theta)$ and $\rho = 0.25$. Results are from 10^5 simulation replicates.

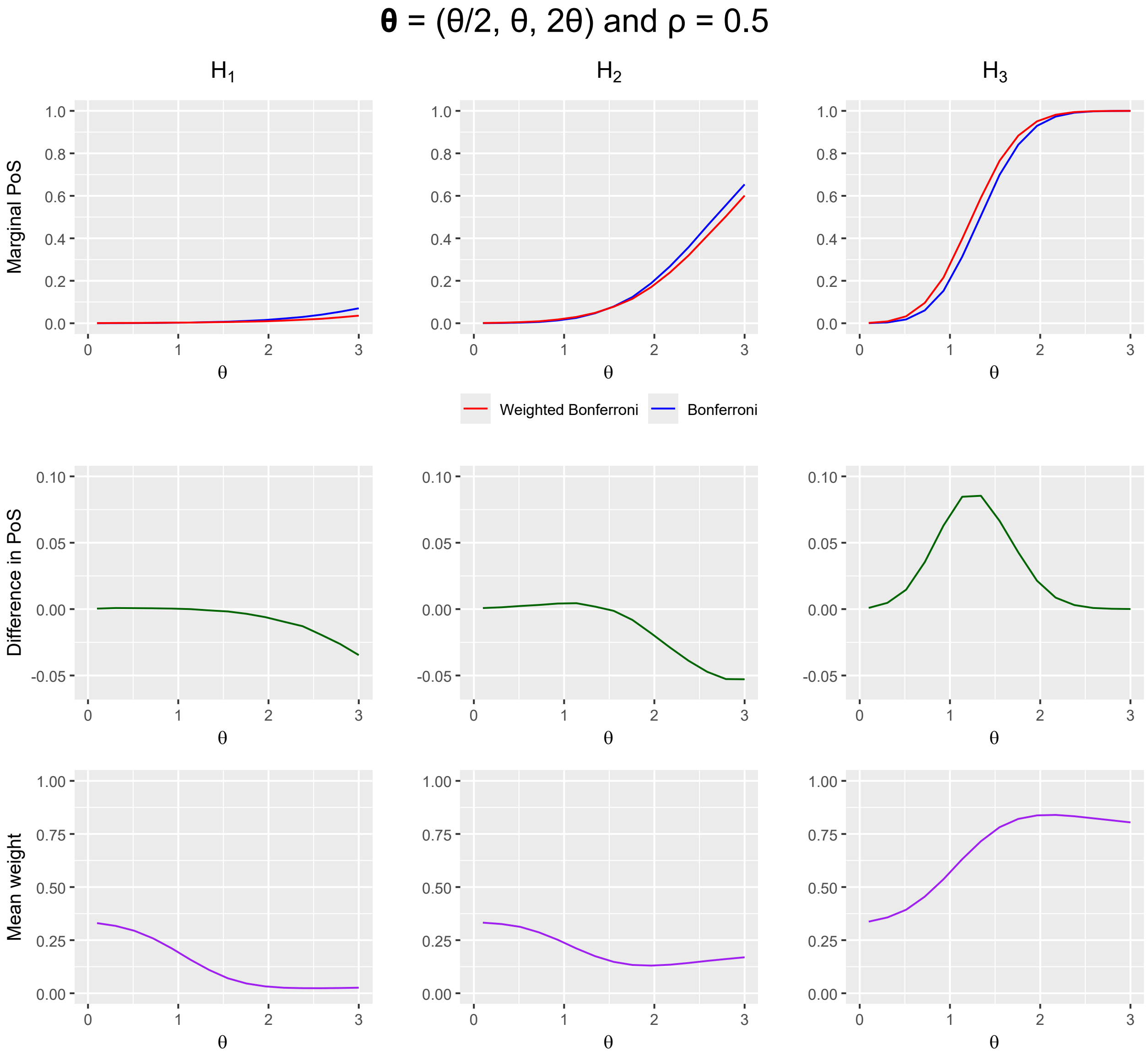

Figure 23: Marginal Probability of Success (mPoS) and mean empirical weights for H_1, H_2, H_3 for $\boldsymbol{\theta} = (\theta/2, \theta, 2\theta)$ and $\rho = 0.5$. Results are from 10^5 simulation replicates.

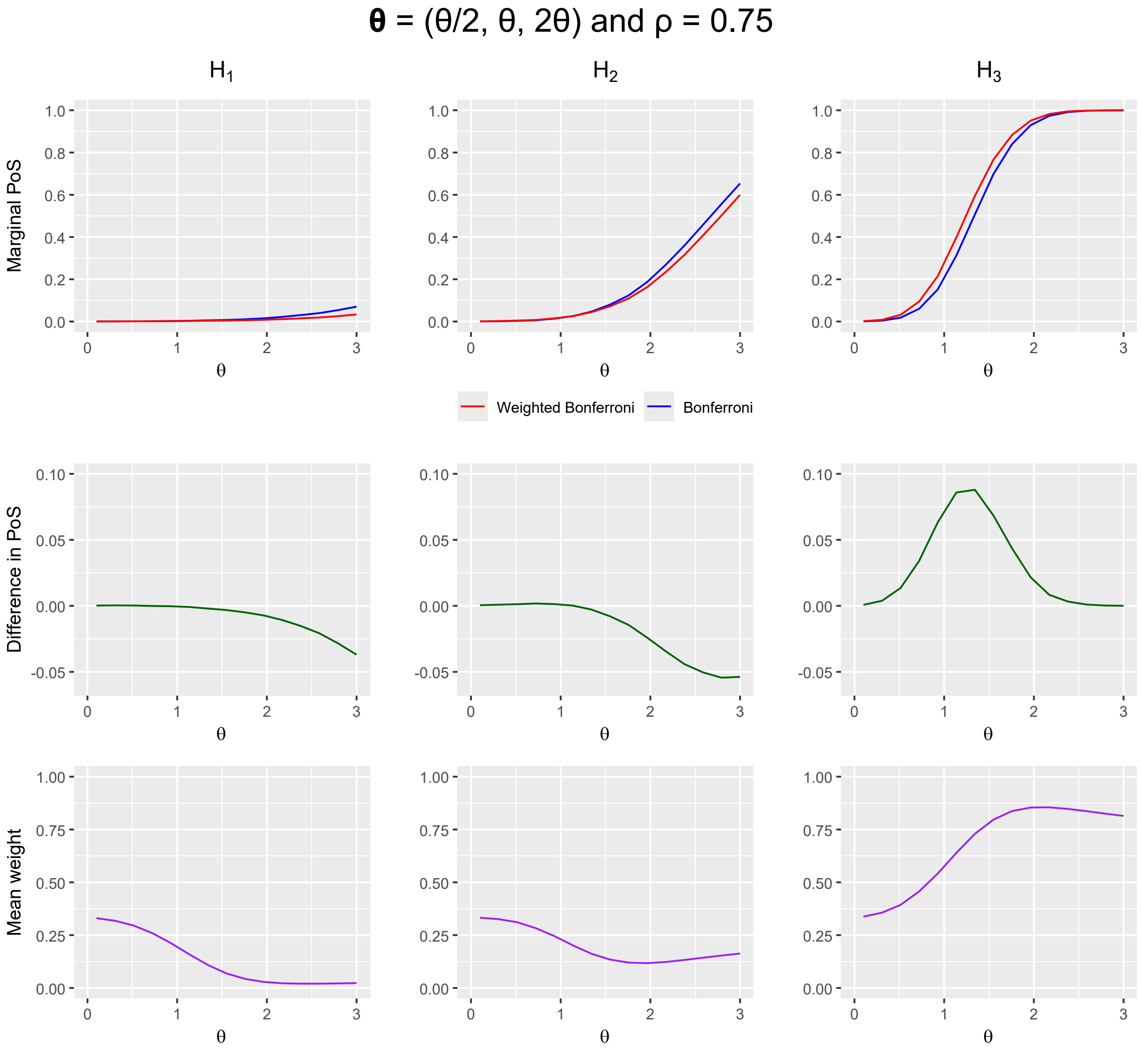

Figure 24: Marginal Probability of Success (mPoS) and mean empirical weights for H_1, H_2, H_3 for $\boldsymbol{\theta} = (\theta/2, \theta, 2\theta)$ and $\rho = 0.75$. Results are from 10^5 simulation replicates.

1.6 Results for $\boldsymbol{\theta} = (0, 0, 0, 0, \theta)$

Figures 25, 26, 27 and 28 show the dPoS (which is the same as the mPoS for H_5) when $\boldsymbol{\theta} = (0, 0, 0, 0, \theta)$, for $\rho = 0, 0.25, 0.5, 0.75$, respectively.

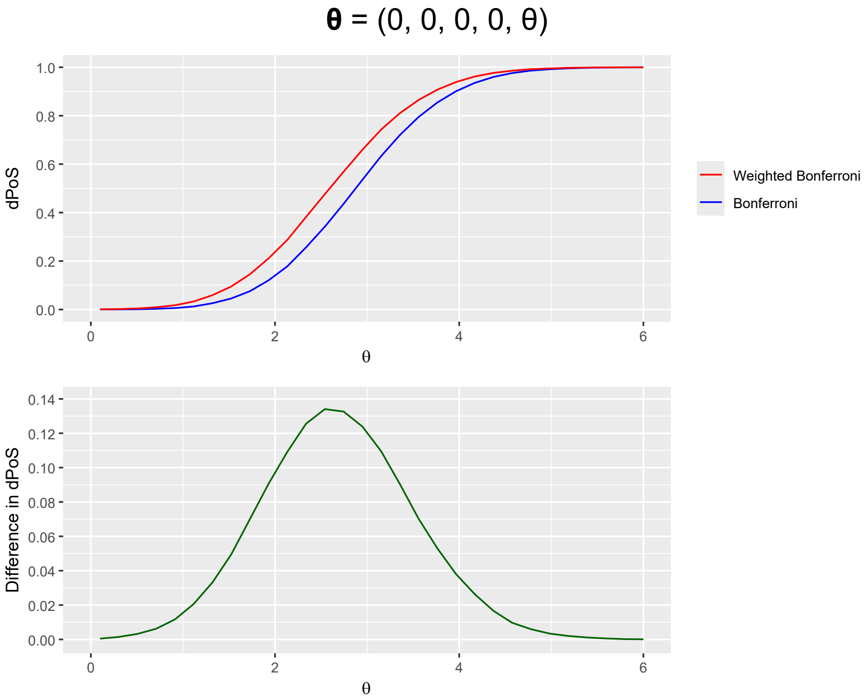

Figure 25: Probability of Success (PoS) for H_5 for $\boldsymbol{\theta} = (0, 0, 0, 0, \theta)$ and $\rho = 0.25$. Results are from 10^5 simulation replicates.

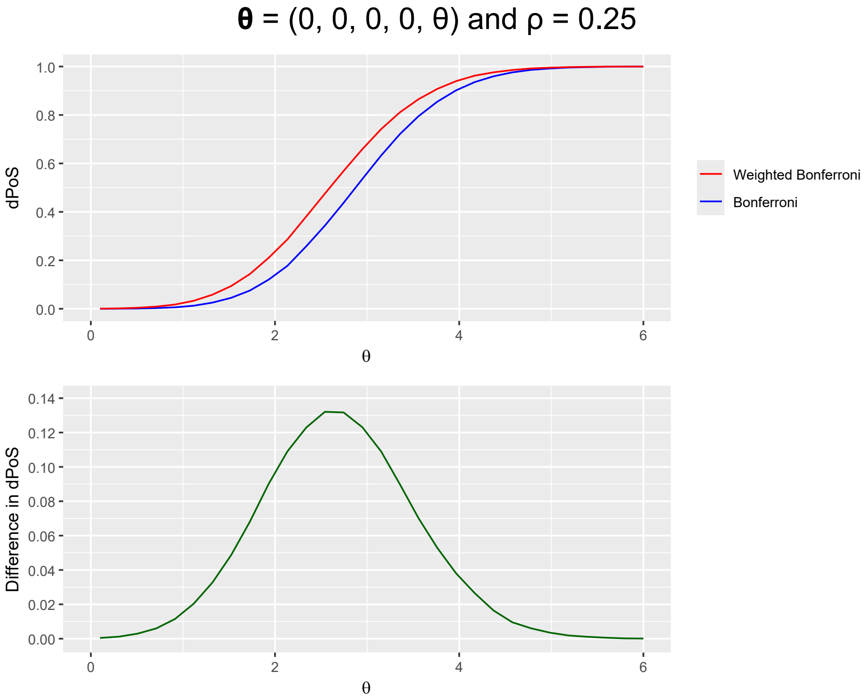

Figure 26: Probability of Success (PoS) for H_5 for $\boldsymbol{\theta} = (0, 0, 0, 0, \theta)$ and $\rho = 0.25$. Results are from 10^5 simulation replicates.

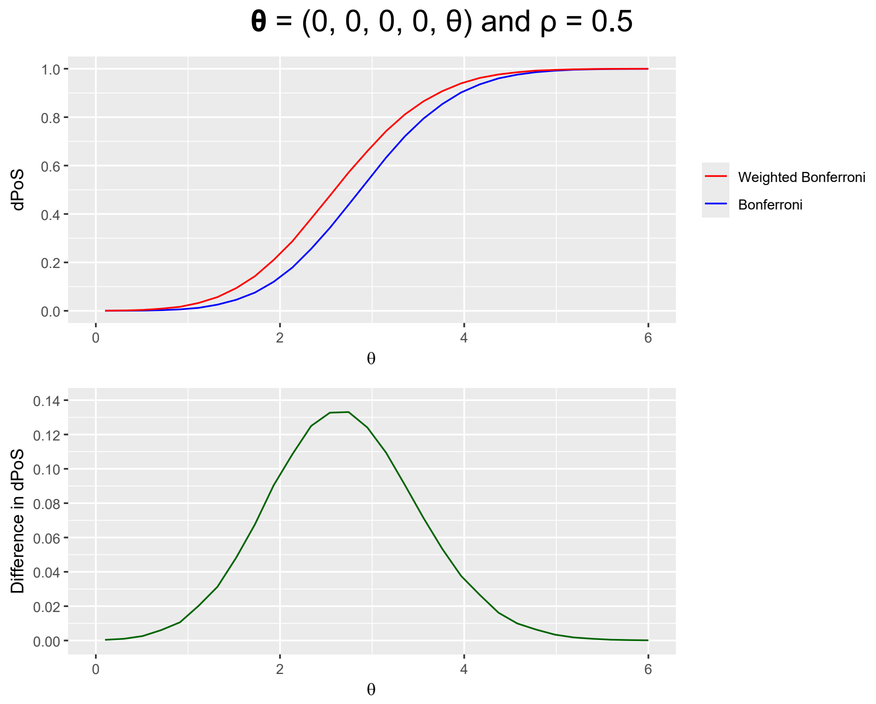

Figure 27: Probability of Success (PoS) for H_5 for $\boldsymbol{\theta} = (0, 0, 0, 0, \theta)$ and $\rho = 0.5$. Results are from 10^5 simulation replicates.

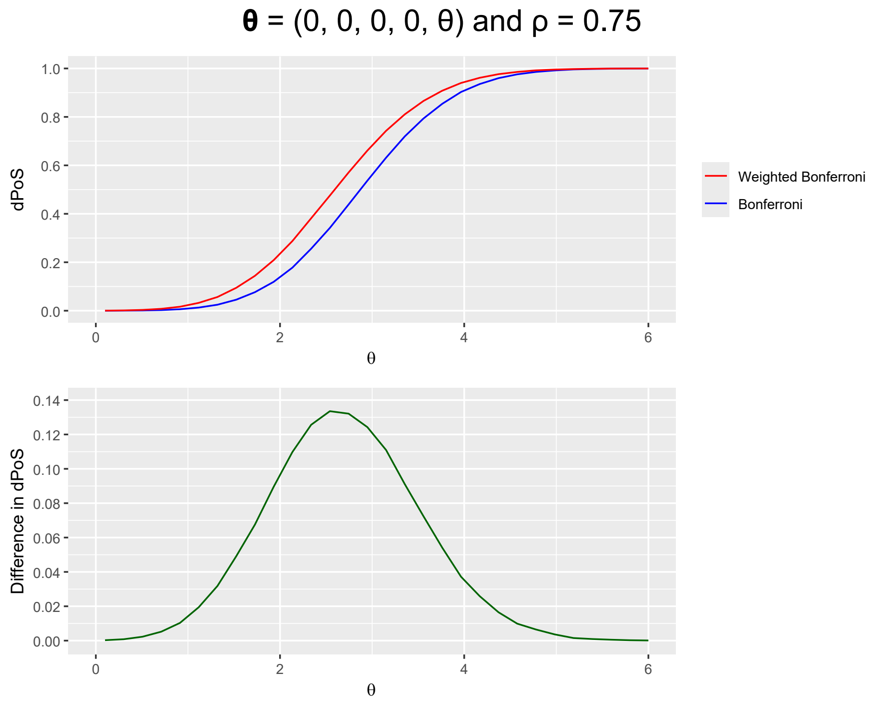

Figure 28: Probability of Success (PoS) for H_5 for $\boldsymbol{\theta} = (0, 0, 0, 0, \theta)$ and $\rho = 0.75$. Results are from 10^5 simulation replicates.

1.7 Results for $\boldsymbol{\theta} = (0, 0, \theta, \theta, \theta)$

Figures 29, 30, 31 and 32 show the dPoS as well as the mPoS for H_5 when $\boldsymbol{\theta} = (0, 0, \theta, \theta, \theta)$, for $\rho = 0, 0.25, 0.5, 0.75$, respectively.

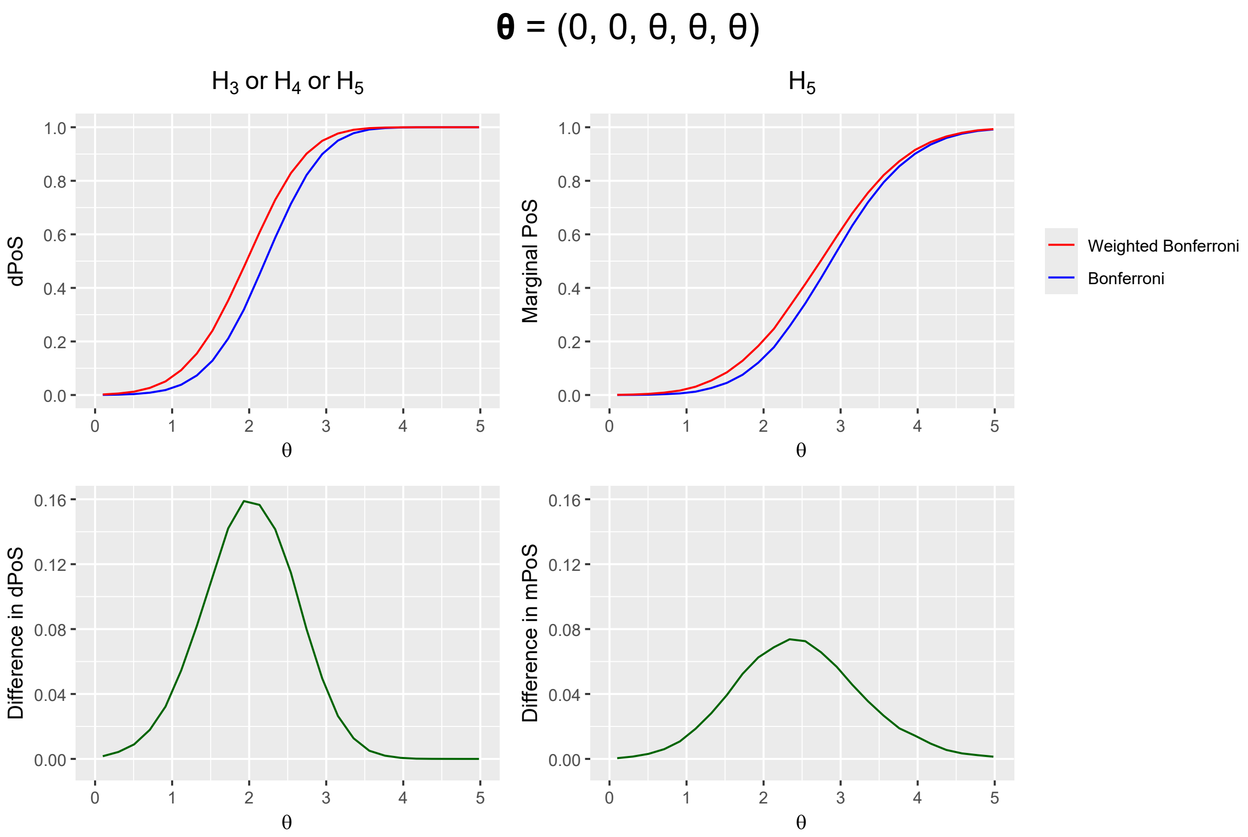

Figure 29: Disjunctive Probability of Success (dPoS) and marginal probability of success (mPoS) for H_5, for $\boldsymbol{\theta} = (0, 0, \theta, \theta, \theta)$ and $\rho = 0$. Results are from 10^5 simulation replicates.

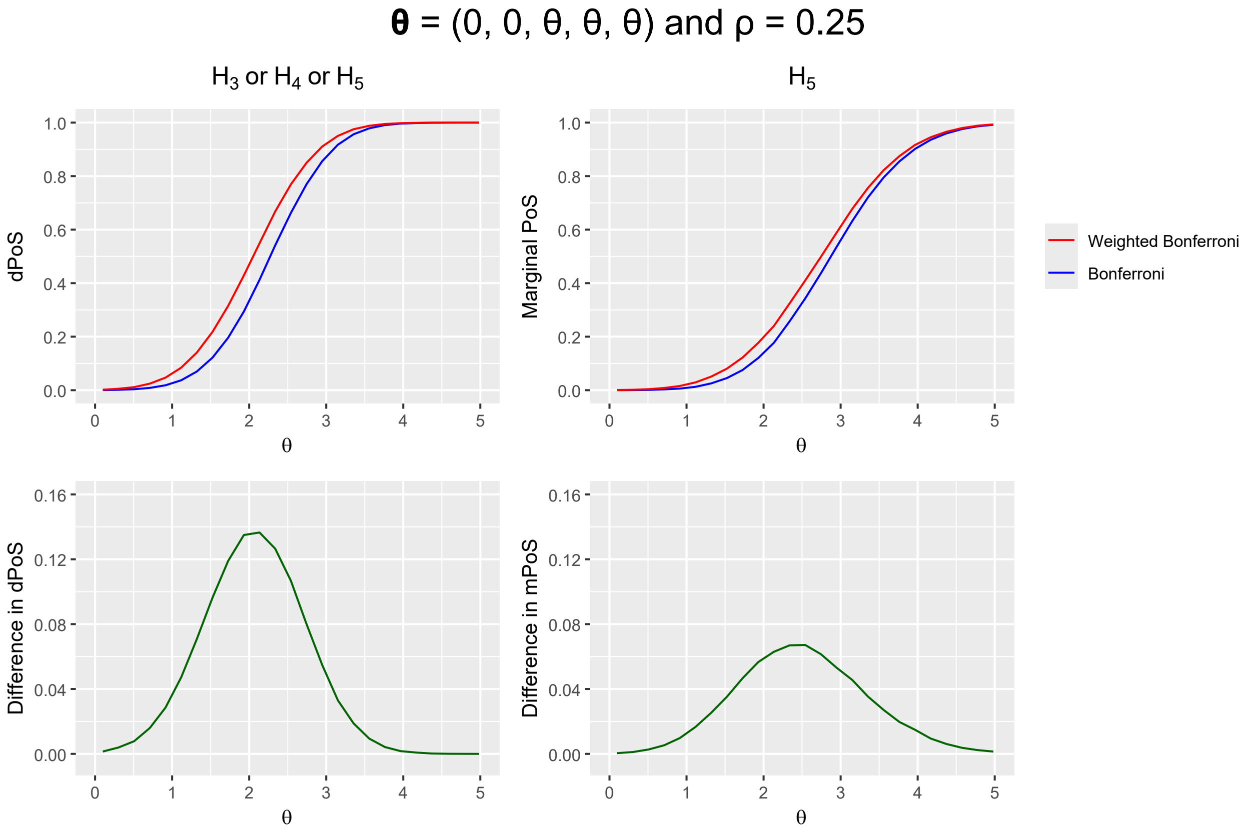

Figure 30: Disjunctive Probability of Success (dPoS) and marginal probability of success (mPoS) for H_5, for $\boldsymbol{\theta} = (0, 0, \theta, \theta, \theta)$ and $\rho = 0.25$. Results are from 10^5 simulation replicates.

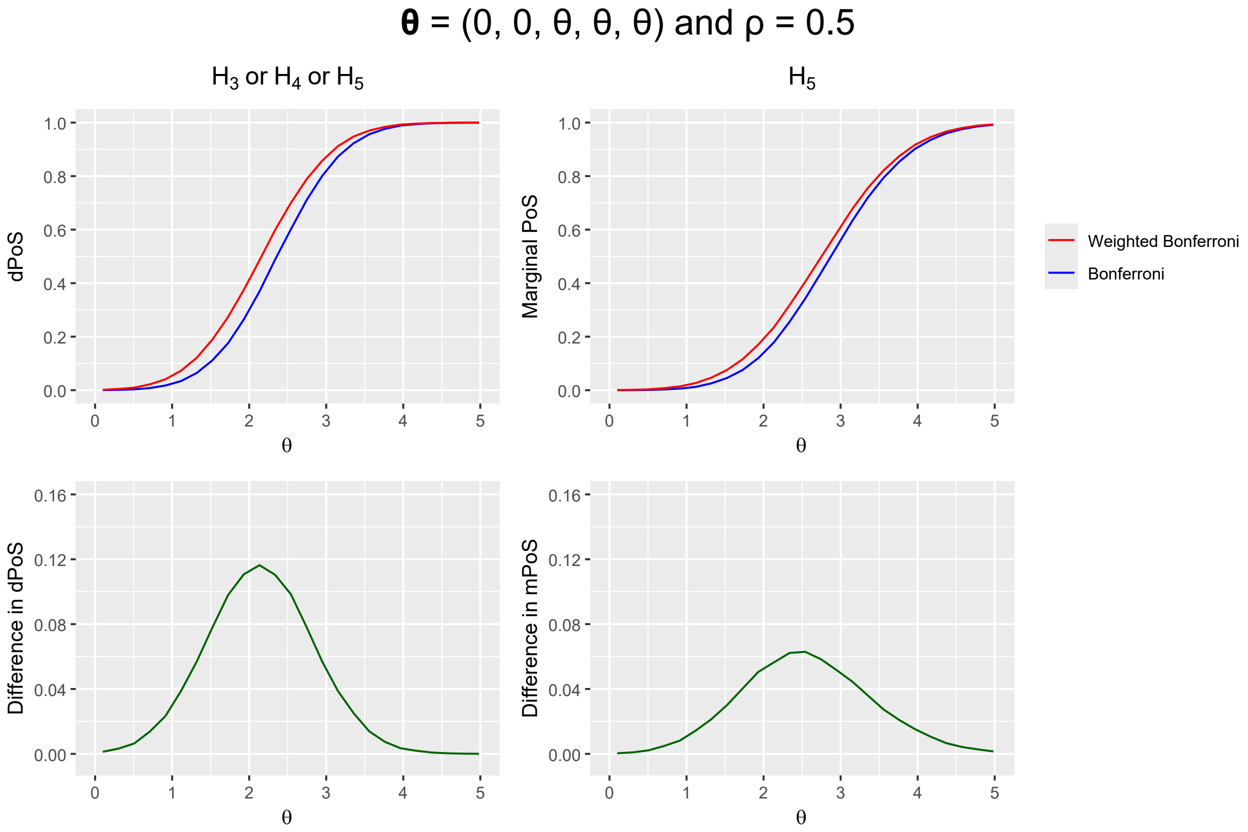

Figure 31: Disjunctive Probability of Success (dPoS) and marginal probability of success (mPoS) for H_5, for $\boldsymbol{\theta} = (0, 0, \theta, \theta, \theta)$ and $\rho = 0.5$. Results are from 10^5 simulation replicates.

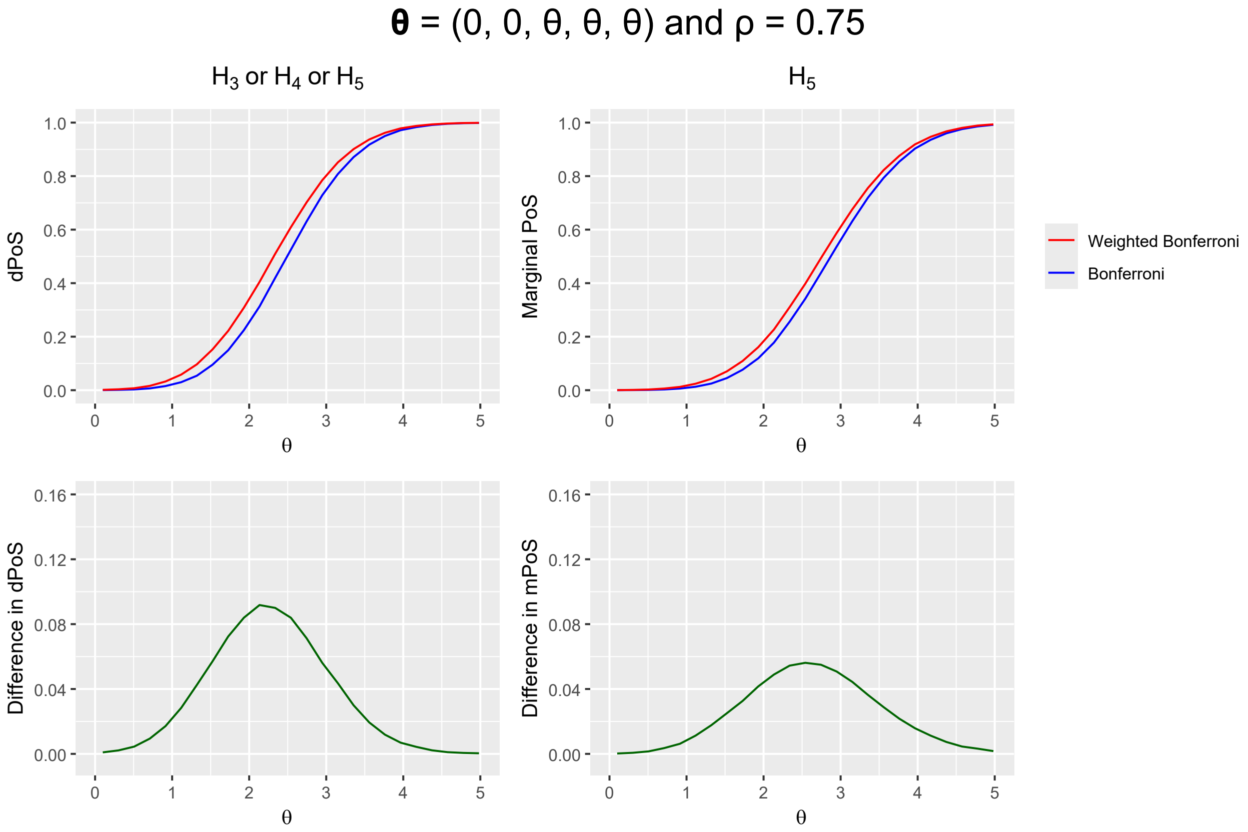

Figure 32: Disjunctive Probability of Success (dPoS) and marginal probability of success (mPoS) for H_5, for $\boldsymbol{\theta} = (0, 0, \theta, \theta, \theta)$ and $\rho = 0.75$. Results are from 10^5 simulation replicates.

1.8 Results for $\boldsymbol{\theta} = (\theta_1, \theta_2, \theta_3, \theta_4, \theta)$ with $\theta_i \sim U[0, \theta]$

Figures 33, 34, 35 and 36 shows the dPoS for $\boldsymbol{\theta} = (\theta_1, \theta_2, \theta_3, \theta_4, \theta)$, with $\theta_i \sim U[0, \theta]$ independently for $i = 1, 2, 3, 4$ and $\theta > 0$, as well as the mPoS for H_5, for $\rho = 0, 0.25, 0.5, 0.75$, respectively.

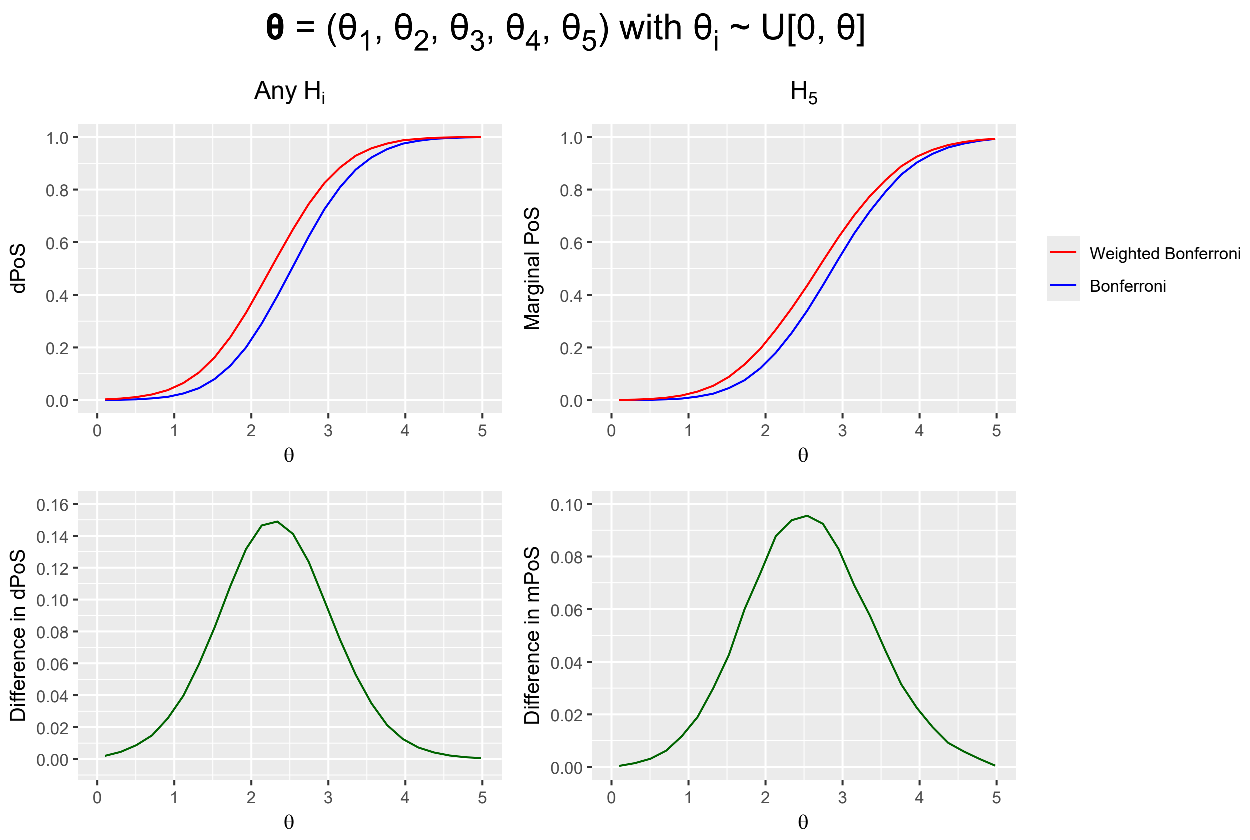

Figure 33: Disjunctive Probability of Success (dPoS) for $\boldsymbol{\theta} = (\theta_1, \theta_2, \theta_3, \theta_4, \theta)$ and marginal Probability of Success (mPoS) for H_5, with $\theta_i \sim U[0, \theta]$ independently for $i = 1, 2, 3, 4$ and $\rho = 0$. Results are from 10^5 simulation replicates.

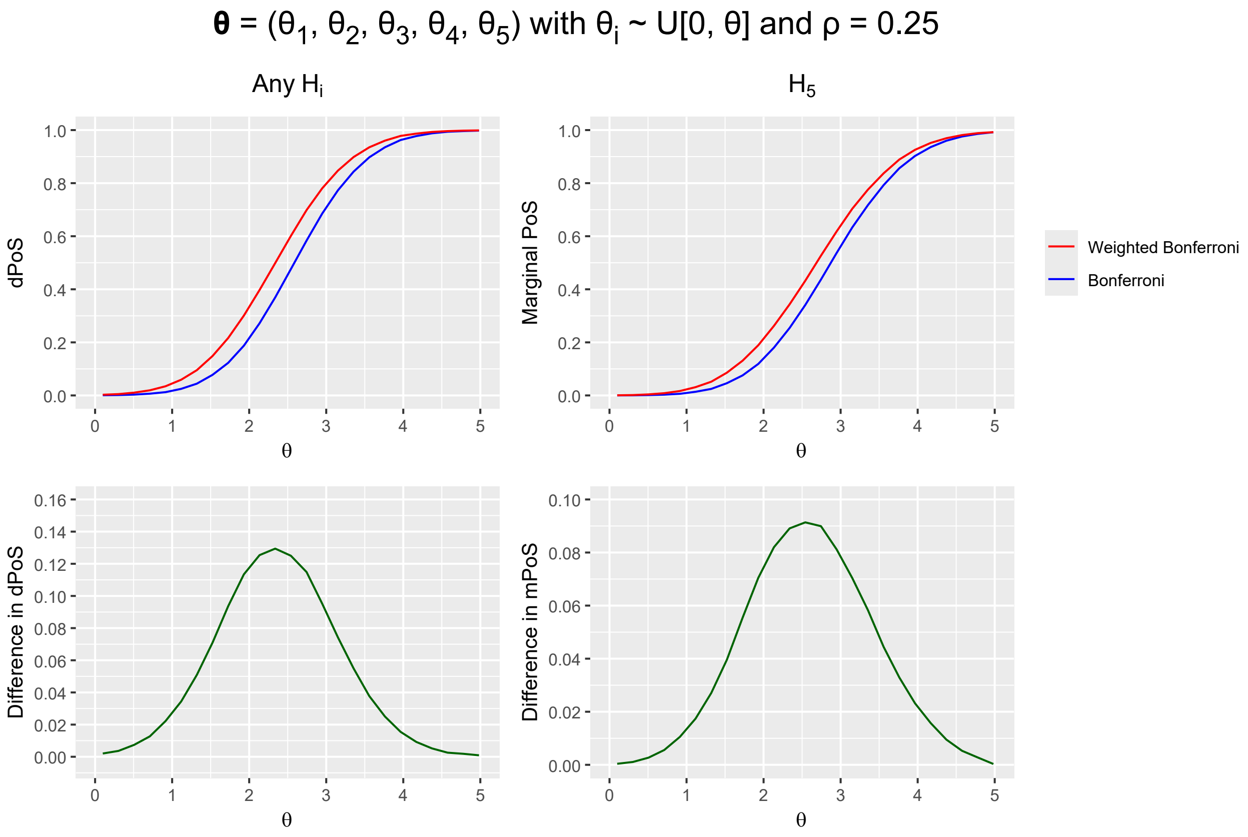

Figure 34: Disjunctive Probability of Success (dPoS) for $\boldsymbol{\theta} = (\theta_1, \theta_2, \theta_3, \theta_4, \theta)$ and marginal Probability of Success (mPoS) for H_5, with $\theta_i \sim U[0, \theta]$ independently for $i = 1, 2, 3, 4$ and $\rho = 0.25$. Results are from 10^5 simulation replicates.

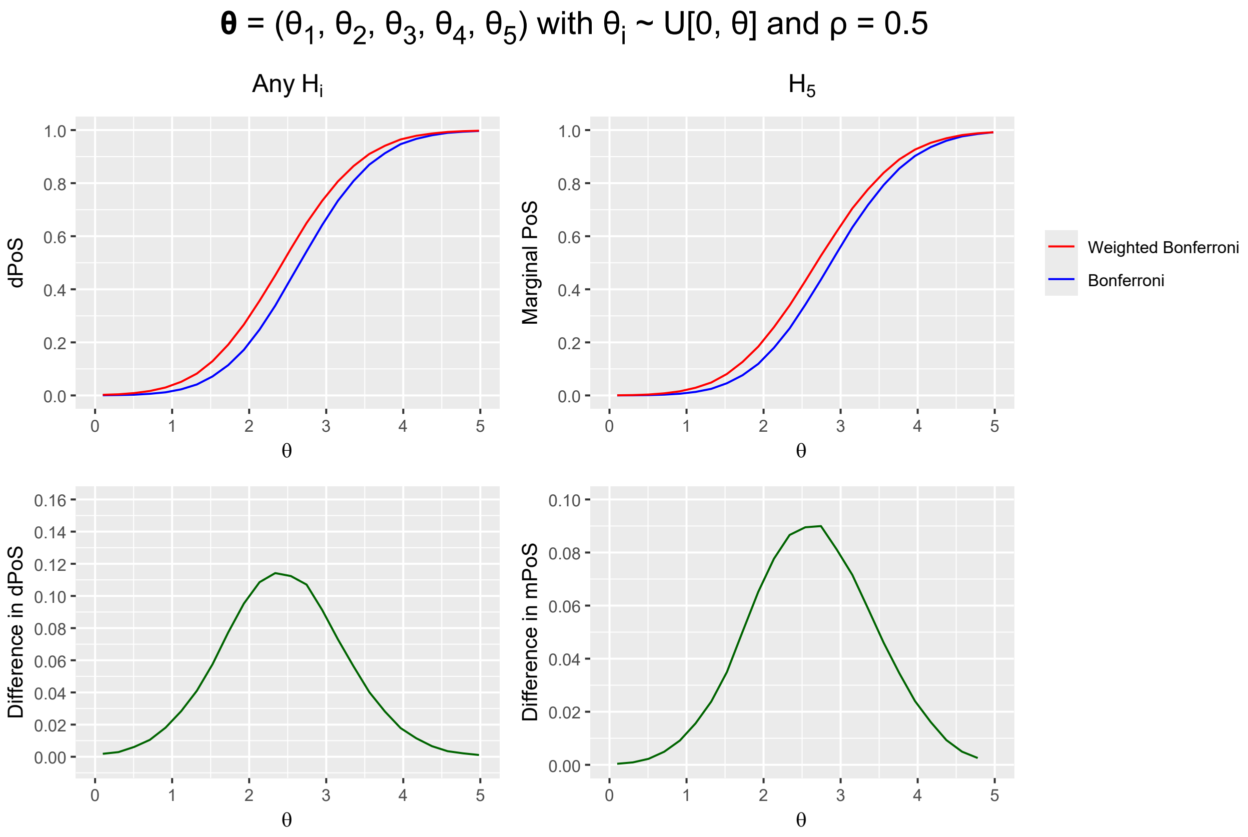

Figure 35: Disjunctive Probability of Success (dPoS) for $\boldsymbol{\theta} = (\theta_1, \theta_2, \theta_3, \theta_4, \theta)$ and marginal Probability of Success (mPoS) for H_5, with $\theta_i \sim U[0, \theta]$ independently for $i = 1, 2, 3, 4$ and $\rho = 0.5$. Results are from 10^5 simulation replicates.

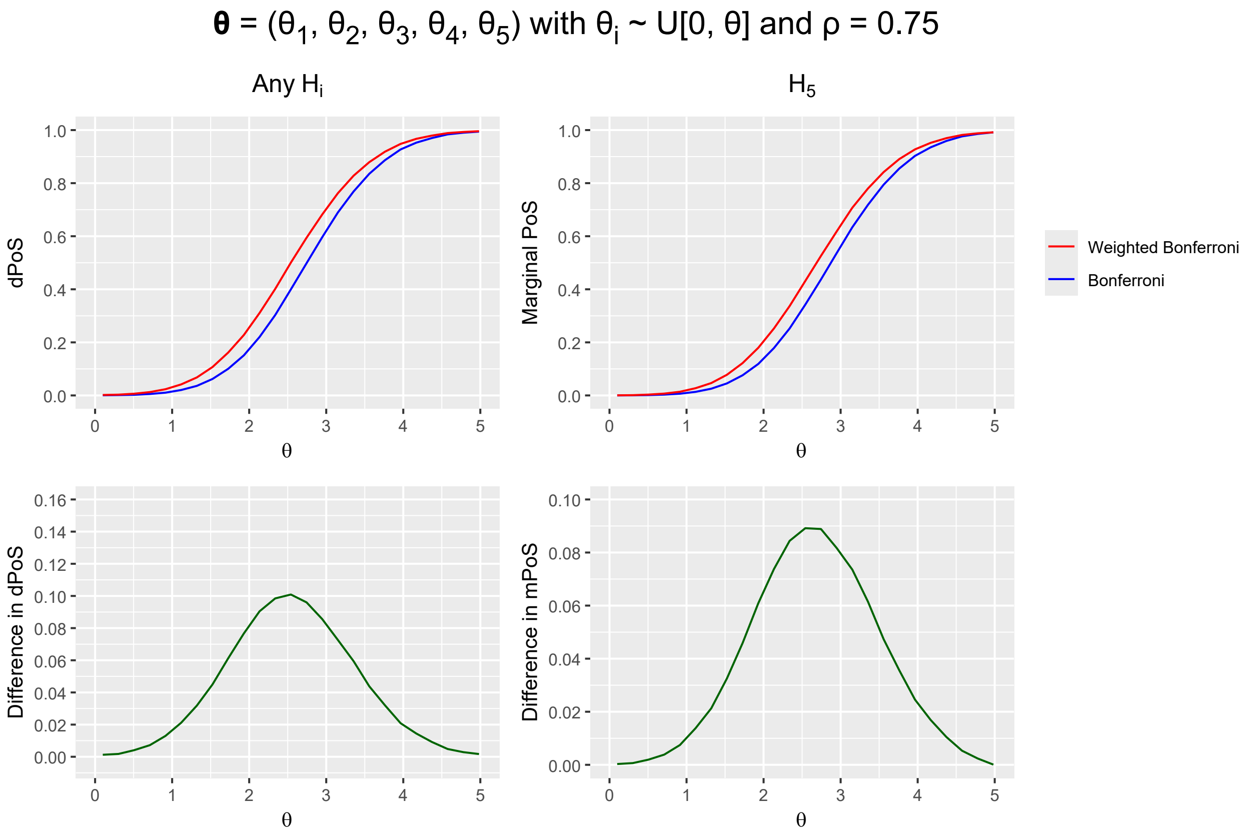

Figure 36: Disjunctive Probability of Success (dPoS) for $\boldsymbol{\theta} = (\theta_1, \theta_2, \theta_3, \theta_4, \theta)$ and marginal Probability of Success (mPoS) for H_5, with $\theta_i \sim U[0, \theta]$ independently for $i = 1, 2, 3, 4$ and $\rho = 0.75$. Results are from 10^5 simulation replicates.
